# Design of Biphenyl-Substituted Diarylpyrimidines with a Cyanomethyl Linker as HIV-1 NNRTIs via a Molecular Hybridization Strategy

**DOI:** 10.3390/molecules25051050

**Published:** 2020-02-26

**Authors:** Yuan Lei, Sheng Han, Yang Yang, Christophe Pannecouque, Erik De Clercq, Chunlin Zhuang, Fen-Er Chen

**Affiliations:** 1 Sichuan Research Center for Drug Precision Industrial Technology, West China School of Pharmacy, Sichuan University, Chengdu 610041, China; smile_leiyuan@163.com (Y.L.); y25571765@163.com (Y.Y.); 2 Engineering Center of Catalysis and Synthesis for Chiral Molecules, Department of Chemistry, Fudan University, Shanghai 200433, China; hansheng8114593@163.com; 3 Shanghai Engineering Center of Industrial Asymmetric Catalysis for Chiral Drugs, Shanghai 200433, China; 4 Rega Institute for Medical Research, KU Leuven, Herestraat 49, B-3000 Leuven, Belgium; christophe.pannecouque@kuleuven.be (C.P.); erik.declercq@kuleuven.be (E.D.C.)

**Keywords:** molecular hybridization, biphenyl, cyanomethyl linker, diarylpyrimidine, HIV-1, reverse transcriptase, nonnucleoside reverse transcriptase inhibitor (NNRTI)

## Abstract

The key problems of human immunodeficiency virus (HIV) therapy are the rapid emergence of drug-resistant mutant strains and significant cumulative drug toxicities. Therefore, there is an urgent demand for new anti-HIV agents with low toxicity and broad-spectrum antiviral potency. A series of biphenyl-substituted diarylpyrimidines with a cyanomethyl linker were designed using a molecular hybridization strategy. The cell-based anti-HIV assay showed that most of the compounds exhibited moderate to good activities against wild-type HIV-1 and clinically relevant mutant strains with a more favorable toxicity, and the enzymatic assay showed they had nanomolar activity against reverse transcriptase (RT). Compound **10p** exhibited the best activity against wild-type HIV-1 with an EC_50_ (50% HIV-1 replication inhibitory concentration) value of 0.027 µM, an acceptable CC_50_ (50% cytotoxic concentration^)^ value of 36.4 µM, and selectivity index of 1361, with moderate activities against the single mutants (EC_50_: E138K, 0.17 µM; Y181C, 0.87 µM; K103N, 0.9 µM; L100I, 1.21 µM, respectively), and an IC_50_ value of 0.059 µM against the RT enzyme, which was six-fold higher than nevirapine (NVP). The preliminary structure–activity relationship (SAR) of these new compounds was concluded. The molecular modeling predicted the binding modes of the new compounds with RT, providing molecular insight for further drug design.

## 1. Introduction

Acquired immune deficiency syndrome (AIDS) is a serious public health problem worldwide caused by the human immunodeficiency virus (HIV) [1,2]. By the end of 2018, there were approximately 37.9 million people living with HIV, which poses a growing threat to the development of mankind [3]. Currently, there are over 30 drugs approved by the U.S. Food and Drug Administration (USA FDA) for AIDS treatment, targeting different steps of the HIV life cycle [4,5]. With the disclosure of highly active antiretroviral therapy (HAART) as the recommended treatment standard for HIV infection, the morbidity and mortality of HIV-infected patients were significantly reduced [6,7]. 

**Figure 1:**
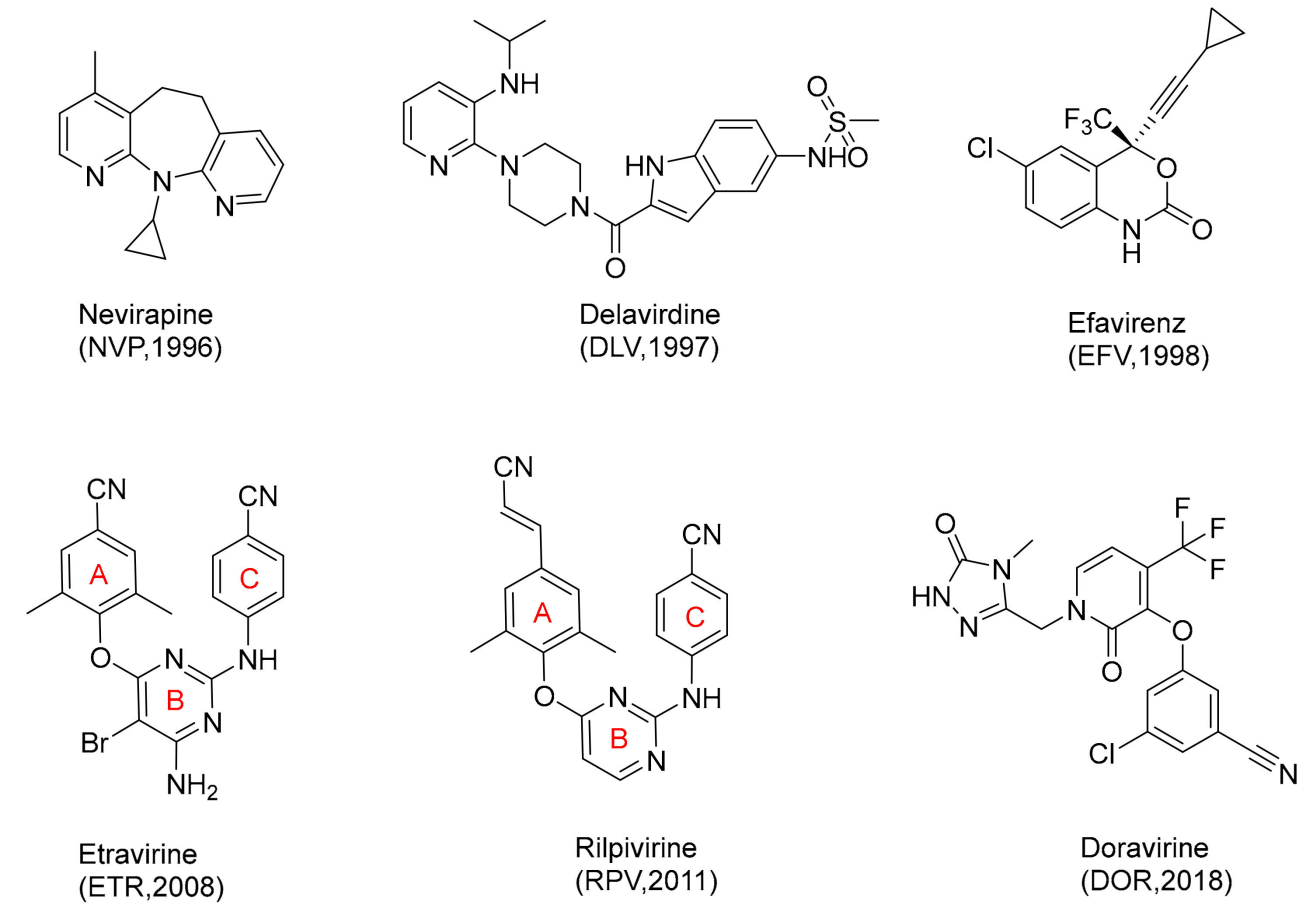
Chemical structures of the FDA-approved nonnucleoside reverse transcriptase inhibitors (NNRTIs).

The nonnucleoside reverse transcriptase inhibitor (NNRTI), as the vital component of the HAART regimen, inhibits the transformation of the single-stranded viral RNA to double-stranded DNA (dsDNA). It binds to an allosteric hydrophobic pocket, also named the NNRTI-binding pocket (NNIBP), located 10 Å from the DNA catalytic active site of reverse transcriptase (RT). With the efforts of the past decades, six NNRTIs were approved by the FDA (Figure 1) [8]. Nevirapine (NVP), delavirdine (DLV), and efavirenz (EFV), the first-generation NNRTIs, bind to NNIBP in a rigid “butterfly-like” binding mode, commonly less active toward the mutant strains [9]. The second-generation NNRTIs, etravirine (ETR) and rilpivirine (RPV), bearing a diarylpyrimidine (DAPY) skeleton, typically have a U or horseshoe shape with torsional (“wiggling”) and repositioning flexibility (“jiggling”), leading to their higher potency against wild-type (WT) and drug-resistant HIV infections [10].

However, adverse events, such as rash and central nervous system (CNS) disturbances and hypersensitivity reactions, were reported in the clinical utility of ETR [11,12]. For, RPV it was reported that a number of drug-resistant mutants lead to virologic failure, and the failure rate is even higher than ETR [13,14,15]. In addition, both ETR and RPV show high cytotoxicity (CC_50_ ~6 µM), which limits long-term therapy of AIDS patients. For these reasons, the efficacy of DAPYs was compromised and the clinic therapeutic options were limited. Therefore, there is an urgent demand for new DAPYs with low toxicity and broad-spectrum antiviral potency. 

**Figure 2:**
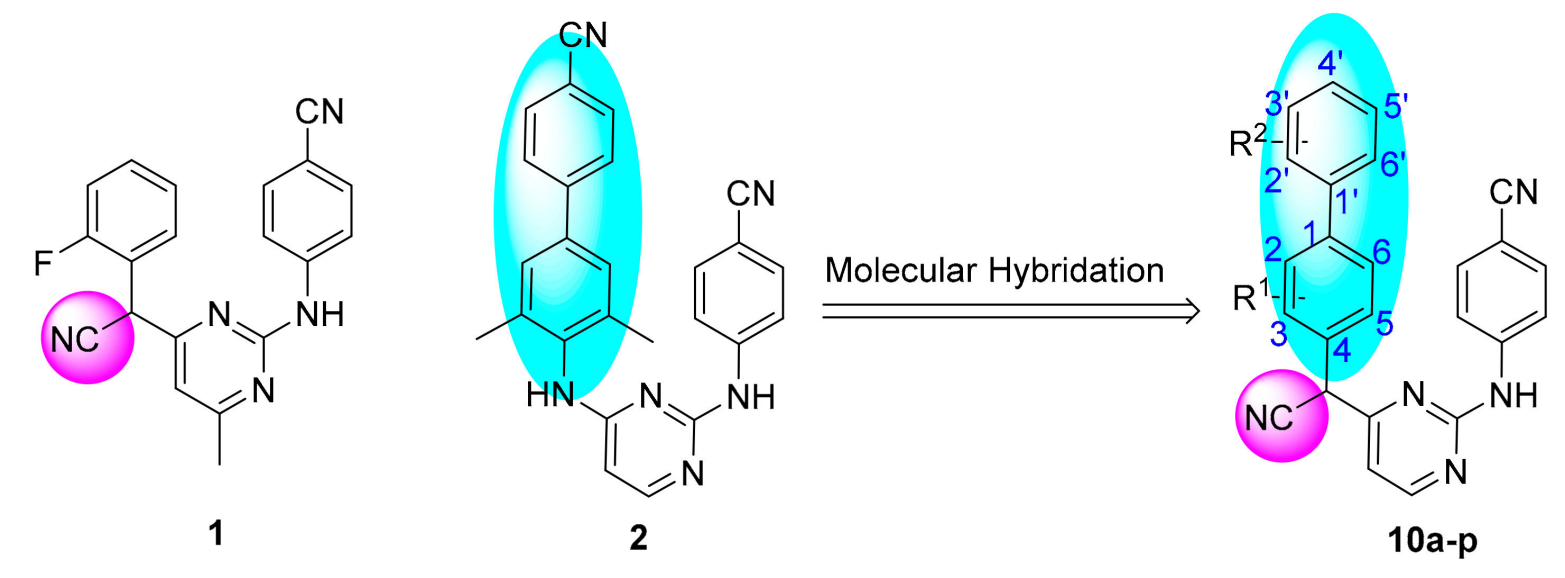
The optimization strategy of CN-biphenyl-diarylpyrimidines (DAPYs).

The CN unit, a bioisostere of various functional groups including carbonyl, halogen, and hydroxyl groups, is widely used in drug discovery, especially in anti-HIV drugs, such as ETR, RPV, DOR, lersivine, dapivirine, and MV-150 [16]. The CN group with a short polarized linear triple bond can stretch into narrow clefts of drug targets, forming hydrogen bonding interactions, polar interactions, covalent interactions, or hydrophobic interactions with amino acids [16,17,18]. Over the past 20 years, our research group made considerable efforts to develop a variety of new DAPY analogues [19,20,21,22]. Incorporating a CN linkage between ring A and the central pyrimidine ring B was determined to be an effective strategy for developing novel DAPY analogues with promising antiviral potency. For instance, compound **1** was a new CN-DAPY with promising anti-HIV potency and selectivity (EC_50_ = 1.8 nM, SI > 118595) (Figure 2) [23]. The biphenyl-DAPYs were developed by our group with low nanomolar EC_50_ values against the WT HIV-1 strain and several clinically resistant strains [24,25,26,27,28]. Compound **2** [29], a representative of biphenyl-DAPYs, showed excellent activities against WT HIV-1 (EC_50_ = 1.0 ± 0.6 nM) and nanomolar EC_50_ values toward several clinically important resistant mutant strains [27]. In the binding with RT, the biphenyl group was attributed to enhancing the π–π interactions with the hydrophobic cavity consisting of Y181, Y188, W229, and F227, and to stabilizing the “U” conformation with the RT enzyme. Inspired by these promising lead compounds, a molecular hybridization strategy was utilized to combine the cyanomethyl linker and biphenyl-DAPY derivatives for developing a new series of scaffolds as NNRTIs (Figure 2).

## 2. Results and Discussion

### 2.1. Chemistry

**Scheme 1:**
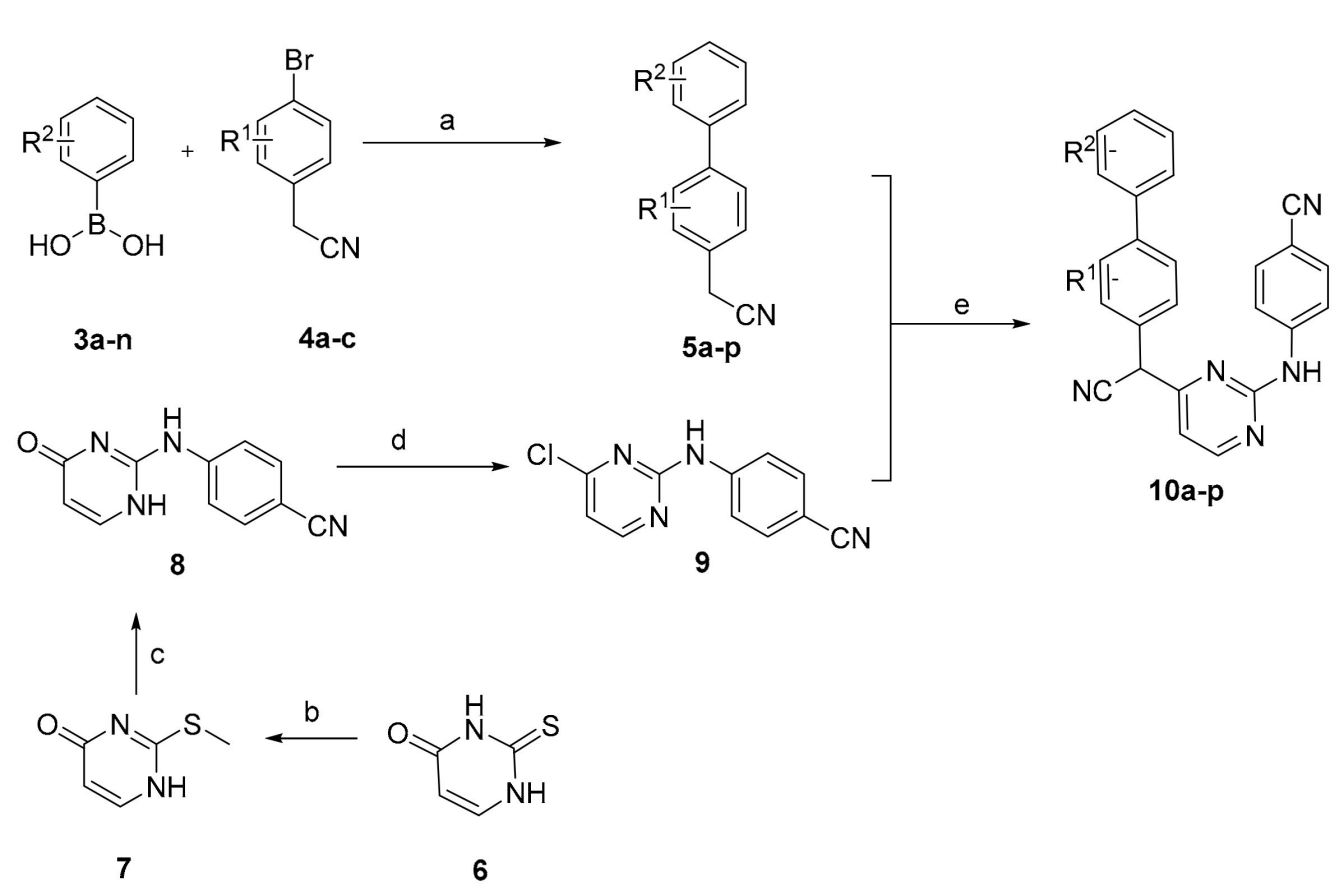
Synthetic route of compounds **10a**–**p**. Reagents and conditions: (a) PdCl_2_, K_2_CO_3_, PEG_400_, H_2_O, 25 °C, 1–4 h; (b) MeI, NaOH, H_2_O, room temperature, 24 h; (c) 4-cyanoaniline, 180–190 °C, 10 h; (d) POCl_3_, reflux, 0.5 h; (e) KOH, DMSO, N_2_, 50 °C, 6 h.

The synthesis of target molecules is depicted in Scheme 1, leading to a mixture of enantiomers. The arylboronic acids **3a**–**n** with different substituted 2-(4-bromophenyl)acetonitriles **4a**–**c** via Suzuki–Miyaura cross-coupling reaction catalyzed by PdCl_2_ provided the key intermediate biphenyl acetonitriles **5a**–**p** in a mixed solvent of PEG_400_ and water at room temperature [30]. The intermediate 4-((4-chloropyrimidin-2-yl)amino)benzonitrile **9** was generated from the commercially available thiouracil **6** through a three-step synthetic strategy according to our previously reported procedures [31,32]. Treatment of **9** with the corresponding biphenyl acetonitriles **5a**–**p** in the presence of KOH in anhydrous DMSO at 50 °C for 4–8 h under a nitrogen atmosphere gave the title compounds **10a**–**p** [33,34].

### 2.2. Biological Activity

The newly synthesized compounds were evaluated for their potency and cytotoxicity against the WT HIV-1 (strain III_B_) in MT-4 cells using the MTT method. The biological evaluation results are represented as EC_50_ values (anti-HIV-1 activity), CC_50_ values (cytotoxicity), and SI values (selectivity index, CC_50_/EC_50_ ratio). Four marketed drugs for HIV-1 infection, including lamivudine (3TC), NVP, ETR, and RPV, were selected as reference drugs (Table 1). 

Firstly, three compounds (**10a**–**c**) with 2-F, 3-F, or H at R^1^ were synthesized. Compound **10c** with 3-F displayed an EC_50_ value of 0.33 µM against the WT HIV-1 viral strain, better than that with H (**10a**) and 2-F (**10b**) substitutions. The compound **10b** with 2-F showed somewhat lower activity than compound **10a** without any substitution, indicating that 2-F substitution had a negative effect on the activity. Their cytotoxicities were decreased compared with the lead compound **2** (CC_50_ = 2.1 µM) [27] and were better than the marketed drugs NVP, ETR, and RPV. Next, the 3-F group at R^1^ was fixed and the effect of R^2^ on the antiviral activity was explored. The electron-donating methyl or methoxy groups were introduced to afford compounds **10d**–**i**. Unfortunately, this series showed decreased activity compared to the corresponding compound **10c** with nonsubstitution at R^2^. The methyl substituted compounds (**10d**–**f**) displayed slightly higher activity than those of methoxy compounds (**10g**–**i**). For **10d**–**f**, the attachment position of the methyl group exerted no apparent effect on HIV potency. For **10g**–**i**, the activity followed the sequence 4’-methoxy > 2’-methoxy > 3’-methoxy. Their cytotoxicities were similar to **10c** with the exception of compound **10i**, which possessed a CC_50_ value of 183 µM. Then, we replaced the electron-donating groups with the electron-withdrawing fluorine and chlorine groups. They exhibited better activity than those of the electron-donating methyl and methoxy groups. Similarly, the activity of the 4’-substituted compound (**10l** and **10o**) was better than those of the 2’- and 3’-substituted analogues (**10l** > **10j** > **10k**, **10o** > **10m** > **10n**). Finally, encouraged by the results, the electron-withdrawing cyano group was introduced to the 4’-position to obtain compound **10p**. To our delight, compound **10p** (EC_50_ = 0.027 µM) exhibited the best activity against WT HIV-1, consistent with our previous research on biphenyl-DAPYs [27]. The activity of **10p** was significantly improved by 12-fold compared with **10c**. The cytotoxicity was also acceptable with a CC_50_ value of 36.4 µM. The selectivity index (SI = 1361) was best in this series, which was better than the marketed drug ETR. Collectively, the above results indicated that the substitution of biphenyl could significantly influence their antiviral potency; 3-F at R^1^ was beneficial to the activity; the electronic effect and position of the R^2^ substitution were key factors for the antiviral activity and satisfied the following orders: CN > F ≈ Cl > CH_3_ > OCH_3_, and 4’-position > 2’-positon > 3’-position, respectively.

These compounds were also tested in the MT-4 cells for their activities against the single mutant (L100I, E138K, Y181C, K103N, Y188L) and double RT mutant (K103N + Y181C, F227L + V106A) HIV-1 strains (Table 2). Most compounds showed submicromolar activities against E138K, Y181C, K103N, and L100I variants. They were more sensitive toward E138K and less active against Y188L. Compounds with 3-F (**10c**–**p**) displayed better activity than the marketed drug 3TC on mutant E138K except for **10h** and **10i**. Notably, compound **10p** exhibited the best EC_50_ values of 0.17, 0.87, 0.90, and 1.21 µM against the single mutant strains E138K, Y181C, K103N, and L100I, especially for the mutant strains of E138K, Y181C, and K103N, which were more active than the reference drugs NVP and 3TC. Unfortunately, these compounds were devoid of satisfactory activity against HIV-1 double mutants (K103N + Y181C, F227L + V106A). 

**Figure 3:**
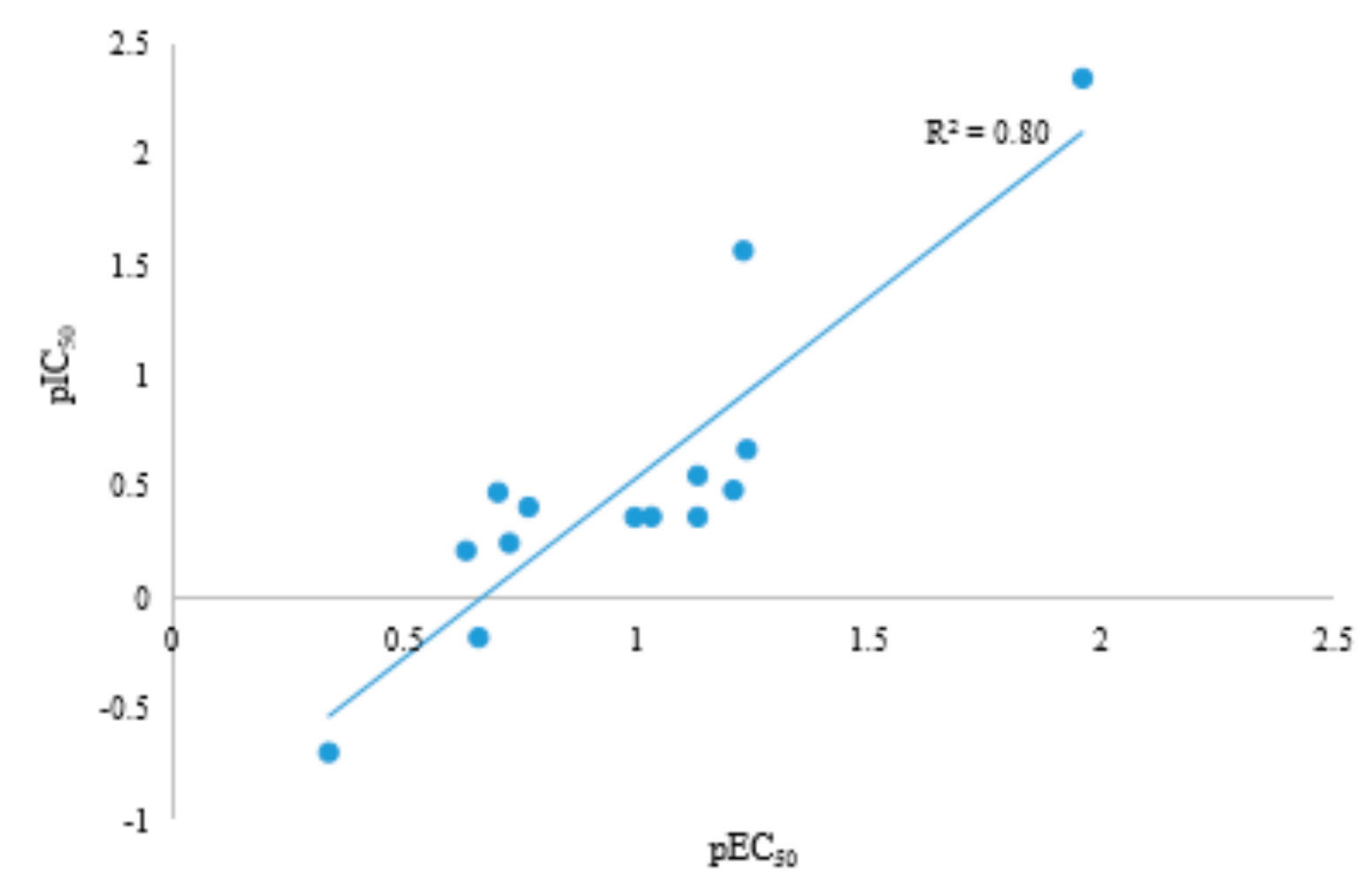
Regression analysis of pIC_50_ vs. pEC_50_ values for CN-biphenyl DAPYs and the reference compounds NVP and ETR.

The enzymatic assay against WT HIV-1 RT was conducted for most compounds (Table 2). All of the target compounds displayed low micromolar RT inhibitory activity (IC_50_ = 0.058–0.460 µM) superior or comparable to that of NVP (IC_50_ = 0.332 µM) except **10b**. Compound **10p** (IC_50_ = 0.059 µM) and **10j** (IC_50_ = 0.058 µM) showed great inhibitory activity toward HIV-1 RT, with values six-fold higher than that of NVP. Moreover, a good linear correlation (*R^2^* = 0.80) between the enzymatic RT inhibitory activities and antiviral activities in MT-4 cells was observed (Figure 3). These results suggested that these compounds could exhibit their anti-HIV-1 activity by targeting RT.

**Figure 4:**
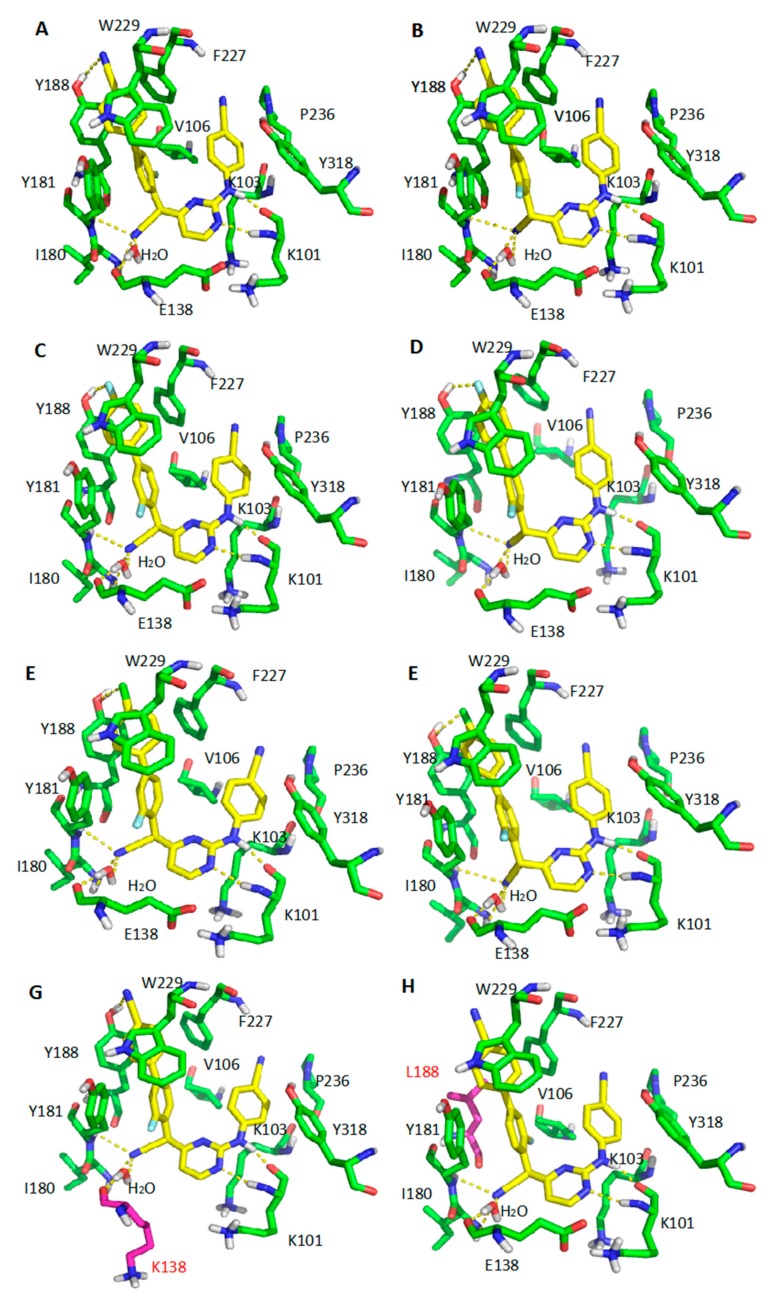
Predicted binding modes of **10l**, **10o**, and **10p** (carbons in yellow) with the HIV-1 WT, E138K, and Y188L mutant RT (PDB: 2ZD1). (**A**) RT with **10p**-*S*; (**B**) RT with **10p**-*R*; (**C**) RT with **10l-***S*; (**D**) RT with **10l-***R*; (**E**) RT with **10o**-*S*; (**F**) RT with **10o**-*R*; (**G**) E138K mutant RT with **10p**-*S*; (**H**) Y188L mutant RT with **10p**-*S*. Residues involved in interactions are shown as green sticks. Mutated residues are depicted as purple sticks. Hydrogen bonds are depicted as yellow dashed lines.

Molecular modeling was performed to provide a deep understanding of the binding modes for explaining the SAR. Compounds **10l**, **10o**, and **10p** with better antiviral activities were selected for docking into NNIBP. Considering the CH(CN) chiral center, we performed the docking study using the two enantiomers. As shown in Figure 4, all compounds could bind to the NNIBP in classical “U” conformation similar to other DAPYs. In general, both enantiomers could form the classic hydrogen-bonding interactions between the backbone of K101 and the N atom of pyrimidine and the NH linker. The biphenyl group could deeply insert into the hydrophobic pocket surrounded by the aromatic residues Y181, Y188, F227, and W229, forming the face-to-face π–π stacking interactions. R/S enantiomers displayed similar binding conformations with NNIBP. The cyanomethyl linker in both enantiomers could form three hydrogen bonds with amino-acid residues I180 and Y181 and a water-mediated hydrogen-bonding interaction with E138 (distance < 3.5 Å). For compound **10p**, the 4’-CN was predicted to form an additional hydrogen bond with Y188 (Figure 4A,B). For compound **10l**, the 4’-F also formed a hydrogen bond with Y188 (Figure 4C,D) while 4’-Cl of compound **10o** formed a halogen bond (Figure 4E,F). In the E138K RT (Figure 4G), the binding mode was similar to the WT RT. However, in the Y188L RT (Figure 4H), the compound **10p** lost the hydrogen bond between 4’-CN and Y188 and decreased π–π stacking interactions. That might explain the higher antiviral activity against the E138K mutant than against the Y188L mutant. The binding modes of **10p** with double mutant (F227L + V106A, K103N + Y181C) RT were also predicted (see Figure S1, Supplementary Materials). As previously mentioned, the “U” conformations of compounds located in the NNIBP were critical for the antiviral activity. However, in the double mutated enzymes generated by Bioluminate, the “U” conformations of compound **10p** (both isomers) were lost probably due to the high steric hindrance caused by the site mutations. The 4-caynoaniline inserted into the entrance channel instead of the solvent-exposed region. The hydrogen-bonding interaction between the NH linker and K101 was missing. The π–π stacking interactions were reduced by Y181C and F227L mutations with decreased aromatic rings. This might explain the decrease in biological activity of compound **10p** against the HIV-1 double mutants.

## 3. Materials and Methods

### 3.1. Apparatus, Materials, and Analytical Reagents

Chemical reagents and solvents were obtained commercially and were used without further purification. All the reactions were monitored by thin-layer chromatography (TLC) on the pre-coated silica gel G plates at 254 nm under an ultraviolet (UV) lamp using ethyl acetate/*n*-hexane as the eluent. Column chromatography was performed on glass column packed with silica gel (200–300 mesh) using ethyl acetate/*n*-hexane as the eluent. Melting points were measured on an SGW X-1 microscopic melting point apparatus. Proton nuclear magnetic resonance (^1^H-NMR) and carbon nuclear magnetic resonance (^13^C-NMR) spectra were recorded in acetone-*d*_6_ at ambient temperature on a Bruker AV-400 MHz spectrometer, using tetramethylsilane (TMS) as the internal standard. High-resolution mass spectrometry (HRMS) was performed on a Bruker Compact instrument using electrospray ionization (ESI). The purities of the compounds were analyzed by HPLC (Agilent 1260) using a C_18_ column (Eslipse XDB, 4.6 × 150 mm, 5 μm) with methanol/water as the mobile phase at a flow rate of 0.8 mL/min: (a) 0–12 min, 50%–95% MeOH; (b) 12–20 min, 95% MeOH; (c) 15–20 min, 60%–95% MeOH; (d) 20–25 min, 95%-50% MeOH; (e) 25–30 min, 50% MeOH. All final compounds exhibited purities greater than 95%. ^1^H-NMR, ^13^C-NMR, and HRMS spectra of the target compounds **10a–p** are presented in Supplementary Materials.

### 3.2. Chemistry 

#### 3.2.1. General Procedure for Synthesis of Compounds **5a**–**p**

A mixture of arylboronic acids **3a**–**n** (5.5 mmol, 1.1 equiv.), different substituted 2-(4-bromophenyl) acetonitriles **4a**–**c** (5.0 mmol, 1.0 equiv.), PdCl_2_ (0.025 mmol, 0.005 equiv.), K_2_CO_3_ (17.5 mmol, 3.5 equiv.), PEG_400_ 15 mL, and H_2_O 15 mL were added to a 100-mL round-bottom flask, and stirred at room temperature for the desired time until complete consumption of starting material as judged by TLC. Then, the reaction mixture was poured into water (100 mL) and extracted with ethyl acetate (30 mL × 3). The combined organic layers were washed with brine, dried over anhydrous Na_2_SO_4_, filtered, and removed by evaporation under reduced pressure to afford the crude products, which were further purified by column chromatography on silica gel eluting with EtOAc/petroleum ether to **5a**–**p** as white solid.

#### 3.2.2. General Procedure for Synthesis of Target Compounds **10a**–**p**

Different substituted biphenylacetonitriles **5a**–**p** (3.0 mmol, 1.0 equiv.) were dissolved in DMSO (10 mL) in the presence of KOH (15.0 mmol, 5.0 equiv.), followed by addition of the compound **9** (3.0 mmol, 1.0 equiv.). The reaction mixture was stirred at 50 °C for 4–8 h (monitored by TLC) under nitrogen atmosphere. Then the reaction was cooled to ambient temperature and poured into H_2_O (200 mL) yielding a precipitate. The precipitate was collected by filtration, and the residue was then purified via flash chromatography on silica gel, eluting with EtOAc/petroleum ether (1/3, *v*/*v*) to obtain final compounds **10a–p** of 33%–84% yield.

(±)-4-((4-([1,1′-biphenyl]-4-yl(cyano)methyl)pyrimidin-2-yl)amino)benzonitrile (**10a**). Yield 65%; white solid; melting point (mp) 207.3–208.8 °C; ^1^H-NMR (400 MHz, acetone-*d*_6_) δ 9.44 (s, 1H, N*H*), 8.59 (t, *J* = 4.8 Hz, 1H, Ar*H*), 8.14–8.06 (m, 2H, Ar*H*), 7.79–7.73 (m, 2H, Ar*H*), 7.71–7.61 (m, 6H, Ar*H*), 7.51–7.42 (m, 2H, Ar*H*), 7.41–7.31 (m, 1H, Ar*H*), 7.10 (t, *J* = 4.8 Hz, 1H, Ar*H*), 5.74 (s, 1H, C*H*); ^13^C-NMR (101 MHz, acetone-*d*_6_) δ 165.01, 159.87, 159.83, 144.48, 141.42, 139.93, 133.60, 132.89, 128.95, 128.76, 127.77, 126.92, 118.99, 118.86, 118.28, 111.39, 104.23, 43.72; HRMS calculated for C_25_H_17_N_5_ [M + H]^+^: 388.1557, found: 388.1572. HPLC analysis: t_R_ = 13.5 min; peak area, 97.6%.

(±)-4-((4-(cyano(2-fluoro-[1,1′-biphenyl]-4-yl)methyl)pyrimidin-2-yl)amino)benzonitrile (**10b**). Yield 33%; white solid; mp 189.6–189.9 °C; ^1^H-NMR (400 MHz, acetone-*d*_6_) δ 9.46 (s, 1H, N*H*), 8.62 (d, *J* = 4.8 Hz, 1H, Ar*H*), 8.11 (d, *J* = 8.5 Hz, 2H, Ar*H*), 7.70 (d, *J* = 8.6 Hz, 2H, Ar*H*), 7.64 (t, *J* = 8.0 Hz, 1H, Ar*H*), 7.58 (d, *J* = 7.1 Hz, 2H, Ar*H*), 7.50 (m, 4H, Ar*H*), 7.42 (d, *J* = 6.4 Hz, 1H, Ar*H*), 7.15 (d, *J* = 4.8 Hz, 1H, Ar*H*), 5.79 (s, 1H, C*H*); ^13^C-NMR (101 MHz, acetone-*d*_6_) δ164.39, 160.04, 159.86, 159.62(d, *J* = 249.3 Hz), 144.44, 135.82 (d, *J* = 8.0 Hz), 134.79, 132.90, 131.77 (d, *J* = 3.9 Hz), 129.34 (d, *J* = 13.6 Hz), 128.92 (d, *J* = 2.9 Hz), 128.63, 128.15, 124.56 (d, *J* = 3.5 Hz), 118.89, 118.82, 117.90, 116.06 (d, *J* = 25.0 Hz), 111.47, 104.31, 43.38; HRMS calculated for C_25_H_16_FN_5_ [M − H]^−^: 404.1317, found: 404.1311. HPLC analysis: t_R_ = 13.7 min; peak area, 96.6%.

(±)-4-((4-(cyano(3-fluoro-[1,1′-biphenyl]-4-yl)methyl)pyrimidin-2-yl)amino)benzonitrile (**10c**). Yield 45%; white solid; mp 178.4–179.6 °C; ^1^H-NMR (400 MHz, acetone-*d*_6_) δ 9.41 (s, 1H, N*H*), 8.63 (d, *J* = 5.0 Hz, 1H, Ar*H*), 8.03 (d, *J* = 8.8 Hz, 2H, Ar*H*), 7.77–7.71 (m, 3H, Ar*H*), 7.70–7.62 (m, 3H, Ar*H*), 7.59–7.52 (m, 1H, Ar*H*), 7.50 (t, *J* = 7.5 Hz, 2H, Ar*H*), 7.44 (d, *J* = 7.3 Hz, 1H, Ar*H*), 7.12 (d, *J* = 5.0 Hz, 1H, Ar*H*), 5.93 (s, 1H, C*H*); ^13^C-NMR (101 MHz, acetone-*d*_6_) δ 163.78, 160.45 (d, *J*_C-F_ = 249.1 Hz), 160.00, 159.81, 144.40, 144.32 (d, *J*_C-F_ = 8.0 Hz), 138.68, 132.83, 130.90 (d, *J*_C-F_= 3.4 Hz), 129.09, 128.42, 126.98, 123.51 (d, *J*_C-F_ = 3.1 Hz), 120.41 (d, *J*_C-F_ = 14.6 Hz), 118.81, 118.74, 117.28, 114.25 (d, *J*_C-F_ = 22.1 Hz), 111.23, 104.24, 38.20 (d, *J*_C-F_= 2.7 Hz); HRMS calculated for C_25_H_16_FN_5_ [M − H]^−^: 404.1317, found: 404.1308. HPLC analysis: t_R_ = 13.7 min; peak area, 97.1%.

(±)-4-((4-(cyano(3-fluoro-2′-methyl-[1,1′-biphenyl]-4-yl)methyl)pyrimidin-2-yl)amino)benzonitrile (**10d**). Yield 54%; white solid; mp 188.7–189.4 °C; ^1^H-NMR (400 MHz, acetone-*d*_6_) δ 9.42 (s, 1H, N*H*), 8.64 (d, *J* = 5.0 Hz, 1H, Ar*H*), 8.06 (d, *J* = 8.4 Hz, 2H, Ar*H*), 7.73 (s, 1H, Ar*H*), 7.66 (d, *J* = 8.7 Hz, 2H, Ar*H*), 7.37–7.23 (m, 6H, Ar*H*), 7.13 (d, *J* = 4.9 Hz, 1H, Ar*H*), 5.94 (s, 1H, C*H*), 2.27 (s, 3H, C*H_3_*); ^13^C-NMR (101 MHz, acetone-*d*_6_) δ 164.78, 160.98, 160.81, 160.71(d, *J*_C-F_ = 249.5Hz), 146.28 (d, *J*_C-F_ = 8.1 Hz), 145.37, 140.67, 135.97, 133.80, 131.52, 131.15 (d, *J*_C-F_= 3.3 Hz), 130.41, 129.04, 127.01, 127.00 (d, *J*_C-F_ = 3.3 Hz), 121.06 (d, *J*_C-F_ = 14.3 Hz), 119.82, 119.74, 118.25, 117.61 (d, *J*_C-F_ = 21.3 Hz), 112.21, 105.26, 39.19 (d, *J*_C-F_ = 2.8 Hz), 20.55; HRMS calculated for C_26_H_18_FN_5_ [M − H]^−^: 418.1473, found: 418.1459. HPLC analysis: t_R_ = 14.3 min; peak area, 97.8%.

(±)-4-((4-(cyano(3-fluoro-3′-methyl-[1,1′-biphenyl]-4-yl)methyl)pyrimidin-2-yl)amino)benzonitrile (**10e**). Yield 46.5%; white solid; mp 186.2-187.6 °C; ^1^H-NMR (400 MHz, acetone-*d*_6_) δ 9.42 (s, 1H, N*H*), 8.63 (d, *J* = 5.0 Hz, 1H, Ar*H*), 8.02 (d, *J* = 8.5 Hz, 2H, Ar*H*), 7.73 (t, *J* = 7.9 Hz, 1H, Ar*H*), 7.65 (t, *J* = 8.2 Hz, 3H, Ar*H*), 7.58–7.50 (m, 3H, Ar*H*), 7.38 (t, *J* = 7.6 Hz, 1H, Ar*H*), 7.25 (d, *J* = 7.5 Hz, 1H, Ar*H*), 7.12 (d, *J* = 5.0 Hz, 1H, Ar*H*), 5.93 (s, 1H, C*H*), 2.41 (s, 3H, C*H_3_*); ^13^C-NMR (101 MHz, acetone-*d*_6_) δ 163.81, 160.44 (d, *J*_C-F_ = 248.8Hz), 159.98, 159.81, 144.54, 144.43 (d, *J*_C-F_ = 5.8 Hz), 138.70, 132.82, 130.83 (d, *J*_C-F_= 3.4 Hz), 129.09, 128.99, 127.67, 124.07, 123.49 (d, *J*_C-F_ = 3.2 Hz), 120.29 (d, *J*_C-F_ = 14.7 Hz), 118.80, 118.73, 117.27, 114.22 (d, *J*_C-F_ = 22.0 Hz), 111.23, 104.25, 38.18 (d, *J*_C-F_ = 2.7 Hz), 20.56; HRMS calculated for C_26_H_18_FN_5_ [M − H]^−^: 418.1473, found: 418.1475. HPLC analysis: t_R_ = 14.5 min; peak area, 97.5%.

(±)-4-((4-(cyano(3-fluoro-4′-methyl-[1,1′-biphenyl]-4-yl)methyl)pyrimidin-2-yl)amino)benzonitrile (**10f**). Yield 54%; white solid; mp 210.5–211.1 °C; ^1^H-NMR (400 MHz, acetone-*d*_6_) δ 9.41 (s, 1H, N*H*), 8.63 (d, *J* = 5.0 Hz, 1H, Ar*H*), 8.03 (d, *J* = 8.5 Hz, 2H, Ar*H*), 7.72 (t, *J* = 7.9 Hz, 1H, Ar*H*), 7.64 (t, *J* = 8.2 Hz, 5H, Ar*H*), 7.55 (d, *J* = 11.8 Hz, 1H, Ar*H*), 7.31 (d, *J* = 7.9 Hz, 2H, Ar*H*), 7.11 (d, *J* = 4.9 Hz, 1H, Ar*H*), 5.92 (s, 1H, C*H*), 2.38 (s, 3H, C*H_3_*); ^13^C-NMR (101 MHz, acetone-*d*_6_) δ 163.84, 160.46 (d, *J*_C-F_ = 248.7Hz), 159.98, 159.80, 144.41, 144.28 (d, *J*_C-F_ = 7.9 Hz), 138.32, 135.78, 132.83, 130.83 (d, *J*_C-F_ = 3.4 Hz), 129.73, 126.81, 123.22 (d, *J*_C-F_= 3.1 Hz), 120.05 (d, *J*_C-F_= 14.7 Hz), 118.81, 118.73, 117.29, 113.93 (d, *J*_C-F_ = 22.0 Hz), 104.24, 38.17 (d, J_C-F_ = 2.8 Hz), 20.21; HRMS calculated for C_26_H_18_FN_5_ [M − H]^−^: 418.1473, found: 418.1472. HPLC analysis: t_R_ = 14.6 min; peak area, 98.3%.

(±)-4-((4-(cyano(3-fluoro-2′-methoxy-[1,1′-biphenyl]-4-yl)methyl)pyrimidin-2-yl)amino)benzonitrile (**10g**). Yield 44%; white solid; mp 184.2–184.7 °C; ^1^H-NMR (400 MHz, acetone-*d*_6_) δ 9.47 (s, 1H, N*H*), 8.66 (d, *J* = 5.0 Hz, 1H, Ar*H*), 8.04 (d, *J* = 8.8 Hz, 2H, Ar*H*), 7.75–7.64 (m, 3H, Ar*H*), 7.58–7.40 (m, 4H, Ar*H*), 7.21–7.14 (m, 2H, Ar*H*), 7.09 (t, *J* = 7.5 Hz, 1H, Ar*H*), 5.96 (s, 1H, C*H*), 3.88 (s, 3H, OC*H_3_*); ^13^C-NMR (101 MHz, acetone-*d*_6_) δ 163.86, 159.98, 159.81, 159.65(d, *J*_C-F_ = 247.7 Hz), 156.53, 144.41, 142.05 (d, *J*_C-F_ = 8.7 Hz), 132.84, 130.48, 129.90 (d, *J*_C-F_ = 3.4 Hz), 129.79, 128.02, 126.11 (d, *J*_C-F_ = 3.1 Hz), 120.93, 119.81 (d, *J*_C-F_ = 14.7 Hz), 118.78, 118.71, 117.33, 116.85 (d, *J*_C-F_ = 21.9 Hz), 111.65, 111.26, 104.20, 55.06, 38.22 (d, *J*_C-F_ = 2.7 Hz); HRMS calculated for C_26_H_18_FN_5_O [M − H]^−^: 434.1423, found: 434.1424. HPLC analysis: t_R_ = 13.6 min; peak area, 96.3%.

(±)-4-((4-(cyano(3-fluoro-3′-methoxy-[1,1′-biphenyl]-4-yl)methyl)pyrimidin-2-yl)amino)benzonitrile (**10h**). Yield 75%; white solid; mp 198.9–199.6 °C; ^1^H-NMR (400 MHz, acetone-*d*_6_) δ 9.45 (s, 1H, N*H*), 8.63 (d, *J* = 5.0 Hz, 1H, Ar*H*), 8.03 (d, *J* = 8.5 Hz, 2H, Ar*H*), 7.76–7.57 (m, 5H, Ar*H*), 7.41 (t, *J* = 7.9 Hz, 1H, Ar*H*), 7.32–7.25 (m, 2H, Ar*H*), 7.12 (d, *J* = 4.9 Hz, 1H, Ar*H*), 7.00 (d, *J* = 7.8 Hz, 1H, Ar*H*), 5.94 (s, 1H, C*H*), 3.88 (s, 3H, OC*H_3_*); ^13^C-NMR (101 MHz, acetone-*d*_6_) δ 164.74, 161.37, 161.34 (d, *J*_C-F_ = 248.5 Hz), 160.94, 160.76, 145.34, 145.21 (d, *J*_C-F_ = 8.1 Hz), 141.10, 133.77, 131.76 (d, *J*_C-F_ = 3.4 Hz), 131.07, 124.55 (d, *J*_C-F_ = 3.1 Hz), 121.47 (d, *J*_C-F_ = 14.5 Hz), 120.16, 119.74, 119.67, 118.19, 115.32 (d, *J*_C-F_ = 22.1 Hz), 115.01, 113.38, 112.18, 105.20, 55.74, 39.13 (d, *J*_C-F_ = 2.7 Hz); HRMS calculated for C_26_H_18_FN_5_O [M − H]^−^: 434.1423, found: 434.1420. HPLC analysis: t_R_ = 13.7 min; peak area, 98.4%.

(±)-4-((4-(cyano(3-fluoro-4′-methoxy-[1,1′-biphenyl]-4-yl)methyl)pyrimidin-2-yl)amino)benzonitrile (**10i**). Yield 84%; white solid; mp 204.9–206.1 °C; ^1^H-NMR (400 MHz, acetone-*d*_6_) δ 9.40 (s, 1H, N*H*), 8.62 (d, *J* = 5.0 Hz, 1H, Ar*H*), 8.04 (d, *J* = 8.7 Hz, 2H, Ar*H*), 7.72–7.61 (m, 6H, Ar*H*), 7.53 (d, *J* = 11.8 Hz, 1H, Ar*H*), 7.10 (d, *J* = 5.0 Hz, 1H, Ar*H*), 7.05 (d, *J* = 8.8 Hz, 2H, Ar*H*), 5.90 (s, 1H, C*H*), 3.86 (s, 3H, OC*H_3_*); ^13^C-NMR (101 MHz, acetone-*d*_6_) δ 163.89, 160.47(d, *J*_C-F_ = 248.5 Hz), 160.26, 159.96, 159.80, 144.41, 144.02 (d, *J*_C-F_ = 8.2 Hz), 132.83, 130.89, 130.77 (d, *J*_C-F_ = 3.5 Hz), 128.12, 122.91 (d, *J*_C-F_ = 3.0 Hz), 119.55 (d, *J*_C-F_ = 14.7 Hz), 118.81, 118.74, 117.31, 114.47, 113.60 (d, *J*_C-F_ = 22.0 Hz), 111.21, 104.25, 54.82, 38.16 (d, *J*_C-F_ = 2.7 Hz); HRMS calculated for C_26_H_18_FN_5_O [M − H]^−^: 434.1423, found: 434.1418. HPLC analysis: t_R_ = 13.6 min; peak area, 97.7%.

(±)-4-((4-(cyano(2′,3-difluoro-[1,1′-biphenyl]-4-yl)methyl)pyrimidin-2-yl)amino)benzonitrile (**10j**). Yield 63%; white solid; mp 192.0–194.4 °C; ^1^H-NMR (400 MHz, acetone-*d*_6_) δ 9.43 (s, 1H, N*H*), 8.64 (d, *J* = 4.9 Hz, 1H, Ar*H*), 8.03 (d, *J* = 8.4 Hz, 2H, Ar*H*), 7.77 (t, *J* = 7.9 Hz, 1H, Ar*H*), 7.69–7.57 (m, 4H, Ar*H*), 7.55–7.44 (m, 2H, Ar*H*), 7.36–7.24 (m, 2H, Ar*H*), 7.14 (d, *J* = 4.9 Hz, 1H, Ar*H*), 5.96 (s, 1H,C*H*); ^13^C-NMR (101 MHz, acetone-*d*_6_) δ 163.67, 160.04, 159.97 (d, *J*_C-F_ = 249.0Hz), 159.83, 159.60 (d, *J*_C-F_ = 248.2 Hz), 144.38, 138.88 (d, *J*_C-F_ = 8.7 Hz), 132.83, 130.76 (d, *J*_C-F_ = 2.9 Hz), 130.60 (d, *J*_C-F_ = 3.3 Hz), 130.45 (d, *J*_C-F_ = 8.5 Hz), 126.72 (d, *J*_C-F_= 14.8 Hz), 125.72 (t, *J*_C-F_ = 3.2 Hz), 125.00 (d, *J*_C-F_ = 3.7 Hz), 121.03 (d, *J*_C-F_= 14.4 Hz), 118.82, 118.75, 117.19, 116.55 (d, *J*_C-F_ = 3.4 Hz), 116.21 (d, *J*_C-F_ = 22.6 Hz), 111.27, 104.28, 38.23 (d, *J*_C-F_ = 2.7 Hz); HRMS calculated for C_25_H_15_F_2_N_5_ [M − H]^−^: 422.1223, found: 422.1217. HPLC analysis: t_R_ = 14.0 min; peak area, 97.5%.

(±)-4-((4-(cyano(3,3′-difluoro-[1,1′-biphenyl]-4-yl)methyl)pyrimidin-2-yl)amino)benzonitrile (**10k**). Yield 65%; pale yellow solid; mp 193.2-194.0 °C; ^1^H-NMR (400 MHz, acetone-*d*_6_) δ 9.42 (s, 1H, N*H*), 8.63 (d, *J* = 5.0 Hz, 1H, Ar*H*), 8.03 (d, *J* = 8.5 Hz, 2H, ArH), 7.79–7.70 (m, 2H, ArH), 7.68–7.61 (m, 3H, ArH), 7.60–7.50 (m, 3H, ArH), 7.20 (t, *J* = 7.7 Hz, 1H, ArH), 7.12 (d, *J* = 5.0 Hz, 1H, ArH), 5.95 (s, 1H, CH); ^13^C-NMR (101 MHz, acetone-d_6_) δ163.68, 163.07(d, *J*_C-F_ = 283.6 Hz), 160.63(d, *J*_C-F_ = 287.5 Hz), 160.02, 159.82, 144.38, 142.82 (d, *J*_C-F_ = 5.7 Hz), 141.07 (d, *J*_C-F_ = 7.5 Hz), 132.83, 131.00 (d, *J*_C-F_ = 2.6 Hz), 130.97 (d, *J*_C-F_ = 8.7 Hz), 123.64 (d, *J*_C-F_ = 3.2 Hz), 122.97 (d, *J*_C-F_ = 2.8 Hz), 121.14 (d, *J*_C-F_ = 14.6 Hz), 118.82, 118.75, 117.19, 115.05 (d, *J*_C-F_ = 21.3 Hz),114.49 (d, *J*_C-F_ = 22.4 Hz), 113.77 (d, *J*_C-F_ = 22.7 Hz), 111.24, 104.29, 38.20 (d, *J*_C-F_ = 2.7 Hz); HRMS calculated for C_25_H_15_F_2_N_5_ [M + H]^+^: 424.1368, found: 424.1368. HPLC analysis: t_R_ = 13.2 min; peak area, 98.2%.

(±)-4-((4-(cyano(3,4′-difluoro-[1,1′-biphenyl]-4-yl)methyl)pyrimidin-2-yl)amino)benzonitrile (**10l**). Yield 63%; white solid; mp 204.2–205.0 °C; ^1^H-NMR (400 MHz, acetone-*d*_6_) δ 9.43 (s, 1H, N*H*), 8.63 (d, *J* = 5.0 Hz, 1H, Ar*H*), 8.02 (t, *J* = 12.3 Hz, 2H, Ar*H*), 7.83–7.72 (m, 3H, Ar*H*), 7.66 (t, *J* = 6.9 Hz, 3H, Ar*H*), 7.58 (d, *J* = 12.8 Hz, 1H, Ar*H*), 7.35–7.23 (m, 2H, Ar*H*), 7.12 (d, *J* = 5.0 Hz, 1H, Ar*H*), 5.94 (s, 1H,C*H*); ^13^C-NMR (101 MHz, acetone-*d*_6_) δ163.75, 163.03 (d, *J*_C-F_ = 247.2 Hz), 160.43 (d, *J*_C-F_ = 248.9 Hz), 160.00, 159.81, 144.38, 143.21 (d, *J*_C-F_ = 8.2 Hz), 135.08, 132.82, 130.93 (d, *J*_C-F_ = 3.4 Hz), 129.06 (d, *J*_C-F_ = 8.3 Hz), 123.46 (d, *J*_C-F_ = 3.1 Hz), 120.47 (d, *J*_C-F_ = 14.6 Hz), 118.82, 118.75, 117.23, 115.82 (d, *J*_C-F_ = 21.8 Hz), 114.25 (d, *J*_C-F_ = 22.3 Hz), 111.23, 104.27, 38.17 (d, *J*_C-F_ = 2.6 Hz); HRMS calculated for C_25_H_15_F_2_N_5_ [M − H]^−^: 422.1223, found: 422.1215. HPLC analysis: t_R_ = 13.2 min; peak area, 98.5%.

(±)-4-((4-((2′-chloro-3-fluoro-[1,1′-biphenyl]-4-yl)(cyano)methyl)pyrimidin-2-yl)amino)benzonitrile (**10m**). Yield 55%; white solid; mp 195.2–196.3 °C; ^1^H-NMR (400 MHz, acetone-*d*_6_) δ 9.42 (s, 1H, N*H*), 8.65 (d, *J* = 5.0 Hz, 1H, Ar*H*), 8.05 (d, *J* = 8.6 Hz, 2H, Ar*H*), 7.76 (t, *J* = 7.9 Hz, 1H, Ar*H*), 7.67 (d, *J* = 8.7 Hz, 2H, Ar*H*), 7.61–7.54 (m, 1H, Ar*H*), 7.50–7.43(m, Ar*H*), 7.40 (d, *J* = 11.1 Hz, 1H, Ar*H*), 7.15 (d, *J* = 5.0 Hz, 1H, Ar*H*), 5.96 (s, 1H, C*H*); ^13^C-NMR (101 MHz, acetone-*d*_6_) δ 163.65, 160.07, 159.85, 159.61 (d, *J*_C-F_ = 249.4 Hz), 144.38, 142.43 (d, *J*_C-F_ = 8.5 Hz), 138.31, 138.29, 132.86, 131.75, 131.43, 130.24 (d, *J*_C-F_ = 3.3 Hz), 130.09, 129.79, 127.51, 126.32 (d, *J*_C-F_ = 3.4 Hz), 121.03 (d, *J*_C-F_ = 14.3 Hz), 118.78, 117.22, 117.05 (d, *J*_C-F_ = 22.2 Hz), 111.27, 104.28, 38.25 (d, *J*_C-F_ = 2.8 Hz); HRMS calculated for C_25_H_15_ClFN_5_ [M − H]^−^: 438.0927, found: 438.0915. HPLC analysis: t_R_ = 13.6 min; peak area, 96.2%.

(±)-4-((4-((3′-chloro-3-fluoro-[1,1′-biphenyl]-4-yl)(cyano)methyl)pyrimidin-2-yl)amino)benzonitrile (**10n**). Yield 59%; white solid; mp 199.6–200.3 °C; ^1^H-NMR (400 MHz, acetone-*d*_6_) δ 9.42 (s, 1H, N*H*), 8.64 (d, *J* = 5.0 Hz, 1H, Ar*H*), 8.04 (d, *J* = 8.7 Hz, 2H, Ar*H*), 7.84–7.61 (m, 7H, Ar*H*), 7.57–7.38 (m, 2H, Ar*H*), 7.13 (d, *J* = 5.0 Hz, 1H, Ar*H*), 5.95 (s, 1H, C*H*); ^13^C-NMR (101 MHz, acetone-*d*_6_) δ 163.68, 160.44(d, *J*_C-F_ = 249.7 Hz), 160.03, 159.83, 144.38, 142.69 (d, *J*_C-F_ = 8.2 Hz), 140.77, 134.56, 132.83, 131.03 (d, *J*_C-F_= 3.4 Hz), 130.73, 128.29, 126.95, 125.58, 123.69 (d, *J*_C-F_ = 3.2 Hz), 121.20 (d, *J*_C-F_= 14.4 Hz), 118.82, 118.75, 117.18, 114.54 (d, *J*_C-F_ = 22.5 Hz), 111.25, 104.30, 38.20 (d, *J*_C-F_ = 2.6 Hz); HRMS calculated for C_25_H_15_ClFN_5_ [M − H]^−^: 438.0927, found: 438.0925. HPLC analysis: t_R_ = 13.6 min; peak area, 96.8%.

(±)-4-((4-((4′-chloro-3-fluoro-[1,1′-biphenyl]-4-yl)(cyano)methyl)pyrimidin-2-yl)amino)benzonitrile (**10o**). Yield 57%; white solid; mp 218.2–219.1 °C; ^1^H-NMR (400 MHz, acetone-*d*_6_) δ 9.41 (s, 1H, N*H*), 8.63 (d, *J* = 5.0 Hz, 1H, Ar*H*), 8.03 (d, *J* = 8.7 Hz, 2H, Ar*H*), 7.76 (t, *J* = 7.8 Hz, 3H, Ar*H*), 7.71–7.58 (m, 4H, Ar*H*), 7.53 (d, *J* = 8.5 Hz, 2H, Ar*H*), 7.12 (d, *J* = 4.9 Hz, 1H, Ar*H*), 5.94 (s, 1H, C*H*); ^13^C-NMR (101 MHz, acetone-*d*_6_) δ 163.70, 160.45(d, *J*_C-F_= 249.3 Hz), 160.03, 159.81, 144.39, 142.91 (d, *J*_C-F_ = 8.2 Hz), 137.42, 134.05, 132.83, 131.03 (d, *J*_C-F_ = 3.4 Hz), 129.12, 128.68, 123.48 (d, *J*_C-F_ = 3.2 Hz), 120.86 (d, *J*_C-F_ = 14.6 Hz), 118.94, 118.82, 117.21, 114.29 (d, *J*_C-F_ = 22.3 Hz), 111.23, 104.25, 38.19 (d, *J*_C-F_ = 2.6 Hz); HRMS calculated for C_25_H_15_ClFN_5_ [M − H]^−^: 438.0927, found: 438.0917. HPLC analysis: t_R_ = 14.1 min; peak area, 98.8%.

(±)-4′-(cyano(2-((4-cyanophenyl)amino)pyrimidin-4-yl)methyl)-3′-fluoro-[1,1′-biphenyl]-4-carbonitrile (**10p**). Yield 48%; pale yellow solid; mp 230.9–231.7 °C; ^1^H-NMR (400 MHz, acetone-*d*_6_) δ 9.37 (s, 1H, N*H*), 8.63 (d, *J* = 5.0 Hz, 1H, Ar*H*), 8.01 (d, *J* = 8.7 Hz, 2H, Ar*H*), 7.98-7.90 (m, 4H, Ar*H*), 7.83–7.75 (m, 2H, Ar*H*), 7.69 (d, *J* = 11.4 Hz, 1H, Ar*H*), 7.63 (d, *J* = 8.8 Hz, 2H, Ar*H*), 7.13 (d, *J* = 5.0 Hz, 1H, Ar*H*), 5.95 (s, 1H, C*H*); ^13^C-NMR (101 MHz, acetone-*d*_6_) δ 163.57, 160.49 (d, *J*_C-F_= 249.7 Hz), 160.04, 159.84, 144.35, 143.01, 142.27 (d, *J*_C-F_ = 8.1 Hz), 132.81 (d, *J*_C-F_ = 4.1 Hz), 131.19 (d, *J*_C-F_ = 3.4 Hz), 127.96, 123.91 (d, *J*_C-F_ = 3.3 Hz), 121.88 (d, *J*_C-F_ = 14.6 Hz), 118.86, 118.79, 118.21, 117.08, 114.76 (d, *J*_C-F_ = 22.6 Hz), 112.00, 111.24, 104.36, 38.23 (d, *J*_C-F_ = 2.7 Hz); HRMS calculated for C_26_H_15_FN_6_ [M − H]^−^: 429.1264, found: 429.1269. HPLC analysis: t_R_ = 11.5 min; peak area, 97.0%.

### 3.3. Biological Assays

The in vitro anti-HIV assay and reverse transcriptase assay were performed as previously described [20].

### 3.4. Molecular Docking

Molecular docking of compounds **10l**, **10o**, and **10p** was performed with the Tripos molecular modeling software packages (Sybyl-X 2.0). The docking studies were performed using the crystal structure of HIV-1 RT (Protein Data Bank (PDB) code: 2ZD1) [22]. The molecular docking results were generated using PyMol (http://pymol.sourceforge.net/). HIV-1 RT was mutated and minimized using Schrödinger BioLuminate. The protein preparation followed our previous protocol [21,27].

## 4. Conclusions

In conclusion, we designed and synthesized 16 new biphenyl-DAPY compounds with a cyanomethyl linker based on a molecular hybridization strategy in order to extend chemical spaces of NNRTIs. Most of the compounds showed submicromolar activity against WT HIV-1 and mutant strains with a more favorable toxicity. The best compound **10p** exhibited EC_50_ values of 0.027, 0.17, 0.87, 0.90, and 1.21 µM against the WT, E138K, Y181C, K103N, and L100I variants, respectively. They also showed a good correlation with their enzymatic activity, indicating that they targeted RT in HIV-1-infected MT-4 cells. Molecular docking predicted the binding modes of these compounds with RT. These cyanomethyl-biphenyl-diarylpyrimidines could be potential lead templates for further optimization in the discovery of anti-HIV-1 drugs.

## Figures and Tables

**Table 1:** Anti-human immunodeficiency virus (HIV-1) activities and cytotoxicity of compounds **10a–p** in MT-4 cells ^a^. 3TC—lamivudine; NVP—nevirapine; ETR—etravirine; RPV—rilpivirine.

Compounds	R^1^	R^2^	EC_50_ (µM) ^b^	CC_50_ (µM) ^c^	SI (Ⅲ_B_) ^d^
			HIV-1 Ⅲ_B_		
**10a**	H	H	2.90 ± 1.89	30.84 ± 8.57	11
**10b**	2-F	H	4.96 ± 0.56	27.34 ± 5.85	6
**10c**	3-F	H	0.33 ± 0.07	23.80 ± 4.48	73
**10d**	3-F	2′-Me	0.43 ± 0.21	30.12 ± 1.94	70
**10e**	3-F	3′-Me	0.43 ± 0.07	26.91 ± 3.78	63
**10f**	3-F	4′-Me	0.43 ± 0.09	30.85 ± 2.76	72
**10g**	3-F	2′-OMe	0.61 ± 0.16	30.05 ± 2.31	49
**10h**	3-F	3′-OMe	1.50 ± 0.24	30.65 ± 2.09	20
**10i**	3-F	4′-OMe	0.51 ± 0.42	183.08 ± 52.24	361
**10j**	3-F	2′-F	0.21 ± 0.05	27.86 ± 3.56	131
**10k**	3-F	3′-F	0.56 ± 0.19	30.68 ± 1.79	55
**10l**	3-F	4′-F	0.18 ± 0.16	25.61 ± 3.58	139
**10m**	3-F	2′-Cl	0.28 ± 0.04	22.52 ± 4.70	81
**10n**	3-F	3′-Cl	0.39 ± 0.10	27.56 ± 4.05	71
**10o**	3-F	4′-Cl	0.18 ± 0.14	27.54 ± 7.30	153
**10p**	3-F	4′-CN	0.027 ± 0.015	36.41 ± 10.57	1361
3TC			7.88 ± 3.15	>87.24	>11
NVP			0.20 ± 0.10	>15.02	>76
ETR			0.0045 ± 0.001	>4.60	>1012
RPV			0.0016 ± 0.0003	5.89 ± 0.31	3747

^a^ All data represent the mean values of at least three independent experiments. ^b^ EC_50_: effective concentration required to protect MT-4 cells against virus-induced cytopathicity by 50%. ^c^ CC_50_: cytotoxic concentration of the compound that reduces the normal uninfected MT-4 cell viability by 50%. ^d^ SI: selectivity index; ratio of CC_50_/EC_50_ (WT).

**Table 2:** The biological activity of the compounds **10a–p** on the mutant HIV-1 virus strains and HIV-1 reverse transcriptase (RT) enzyme.

Compounds	R1	R2	EC50 (µM)							IC50 (µM) a
			L100I	E138K	Y181C	K103N	Y188L	F227L + V106A	K103N + Y181C	
**10a**	H	H	>30.84	≥12.62	>30.84	>30.84	>30.84	>30.84	>30.84	ND b
**10b**	2-F	H	≥27.33	9.29 ± 1.37	>27.33	>27.33	>27.33	>27.33	>27.33	0.460 ± 0.162
**10c**	3-F	H	≥10.26	1.69 ± 0.17	6.37 ± 0.78	10.32 ± 0.85	>23.80	>23.8	>23.8	0.062 ± 0.024
**10d**	3-F	2′-Me	3.67 ± 0.54	2.5 ± 0.33	4.89 ± 1.07	8.14 ± 1.82	>30.11	>30.11	>30.11	0.074 ± 0.024
**10e**	3-F	3′-Me	9.14 ± 3.61	3.59 ± 1.03	>26.92	>26.92	>26.92	>26.92	>26.92	0.101 ± 0.059
**10f**	3-F	4′-Me	6.12 ± 1.62	2.55 ± 0.17	9.27 ± 1.14	8.08 ± 2.74	>30.85	>30.85	>30.85	0.093 ± 0.051
**10g**	3-F	2′-OMe	4.27 ± 0.46	3.66 ± 0.19	7.79 ± 1.67	9.78 ± 1.60	>30.04	>30.04	>30.04	0.233 ± 0.125
**10h**	3-F	3′-OMe	8.57 ± 1.26	6.16 ± 1.47	>30.66	>30.66	>30.66	>30.66	>30.66	0.219 ± 0.171
**10i**	3-F	4′-OMe	31.56 ± 7.35	5.51 ± 1.06	8.57 ± 1.21	26.97 ± 3.00	50.10 ± 6.52	52.45	>183.07	ND
**10j**	3-F	2′-F	6.06 ± 2.46	1.03 ± 0.17	3.4 ± 0.48	3.93 ± 0.65	>27.87	>27.87	>27.87	0.058 ± 0.032
**10k**	3-F	3′-F	9.29 ± 0.57	3.36 ± 0.4	9.15 ± 0.87	>30.68	>30.68	>30.68	>30.68	0.188 ± 0.172
**10l**	3-F	4′-F	6.90 ± 1.76	0.98 ± 0.13	5.1 ± 0.51	6.87 ± 1.17	>25.60	>25.6	>25.6	ND
**10m**	3-F	2′-Cl	5.61 ± 1.51	2.43 ± 0.5	4.53 ± 1.47	5.34 ± 0.93	>22.53	>22.53	>22.53	0.074 ± 0.024
**10n**	3-F	3′-Cl	8.31 ± 1.20	2.67 ± 0.21	9.19 ± 2.05	7.2 ± 0.89	>27.55	>11.21	>27.55	0.171 ± 0.132
**10o**	3-F	4′-Cl	5.28 ± 1.29	1.50 ± 0.28	7.49 ± 1.64	7.32 ± 0.87	>27.53	>27.53	>27.53	ND
**10p**	3-F	4′-CN	1.21 ± 0.21	0.17 ± 0.03	0.87 ± 0.23	0.9 ± 0.27	9.81 ± 1.30	>25.6	>36.4	0.059 ± 0.034
3TC			1.96 ± 0.29	5.06 ± 0.90	4.74 ± 1.31	3.98 ± 0.76	3.42 ± 0.95	2.69 ± 0.87	5.50 ± 1.31	ND
NVP			0.98 ± 0.39	0.20 ± 0.15	6.07 ± 1.55	4.70 ± 0.63	≥4.62	≥4.281	>15.02	0.332 ± 0.11
ETR			0.008 ± 0.002	0.008 ± 0.004	0.017 ± 0.003	0.003 ± 0.001	0.017 ± 0.006	0.014 ± 0.005	0.04 ± 0.01	0.009 ± 0.003
RPV			0.002 ± 0.001	0.003 ± 0.001	0.003 ± 0.001	0.001 ± 0.0002	0.03 ± 0.009	0.044 ± 0.012	0.009 ± 0.002	ND

^a^ IC_50_: inhibitory concentration of test compound required to inhibit wild-type (WT) HIV-1 RT polymerase activity by 50%. ^b^ ND: not determined.

## Supplementary Materials

The following are available online at https://www.mdpi.com/1420-3049/25/5/1050/s1: Figure S1: Molecular docking study for double mutants with **10p;** NMR and HRMS spectra for the target compounds **10a–p**.
