# Supplementary material for: Design of Biphenyl-Substituted Diarylpyrimidines with a Cyanomethyl Linker as HIV-1 NNRTIs via a Molecular Hybridization Strategy
Source: Molecules. 2020 Feb 26;25(5):1050. doi: 10.3390/molecules25051050 (PMC7179183; doi:10.3390/molecules25051050)
Supplement: Supplementary file 1 [file molecules-25-01050-s001.pdf]

Supporting Information for

Discovery of novel biphenyl-substituted diarylpyrimidines with a cyanomethyl linker as HIV-1 NNRTIs by a molecular hybridization strategy Design of Biphenyl-Substituted Diarylpyrimidines with a Cyanomethyl Linker as HIV-1 NNRTIs via a Molecular Hybridization Strategy

Yuan Lei [1,†], Sheng Han [2,3,†], Yang Yang [1], Christophe Pannecouque [4], Erik De Clercq [4], Chunlin Zhuang [2,3,*] and Fen-Er Chen [1,2,3,*]

1 Sichuan Research Center for Drug Precision Industrial Technology, West China School of Pharmacy, Sichuan University, Chengdu 610041, China; smile_leiyuan@163.com (Y.L.), y25571765@163.com(Y.Y.)

2 Engineering Center of Catalysis and Synthesis for Chiral Molecules, Department of Chemistry, Fudan University, Shanghai 200433, China; hansheng8114593@163.com

3 Shanghai Engineering Center of Industrial Asymmetric Catalysis for Chiral Drugs, Shanghai 200433, China

4 Rega Institute for Medical Research, KU Leuven, Herestraat 49, B-3000 Leuven, Belgium; christophe.pannecouque@kuleuven.be (C.P.); erik.declercq@kuleuven.be (E.D.C.)

* Correspondence: zclnathan@163.com (C.Z.), rfchen@fudan.edu.cn (F.-E.C.)

† These two authors contributed equally to this work

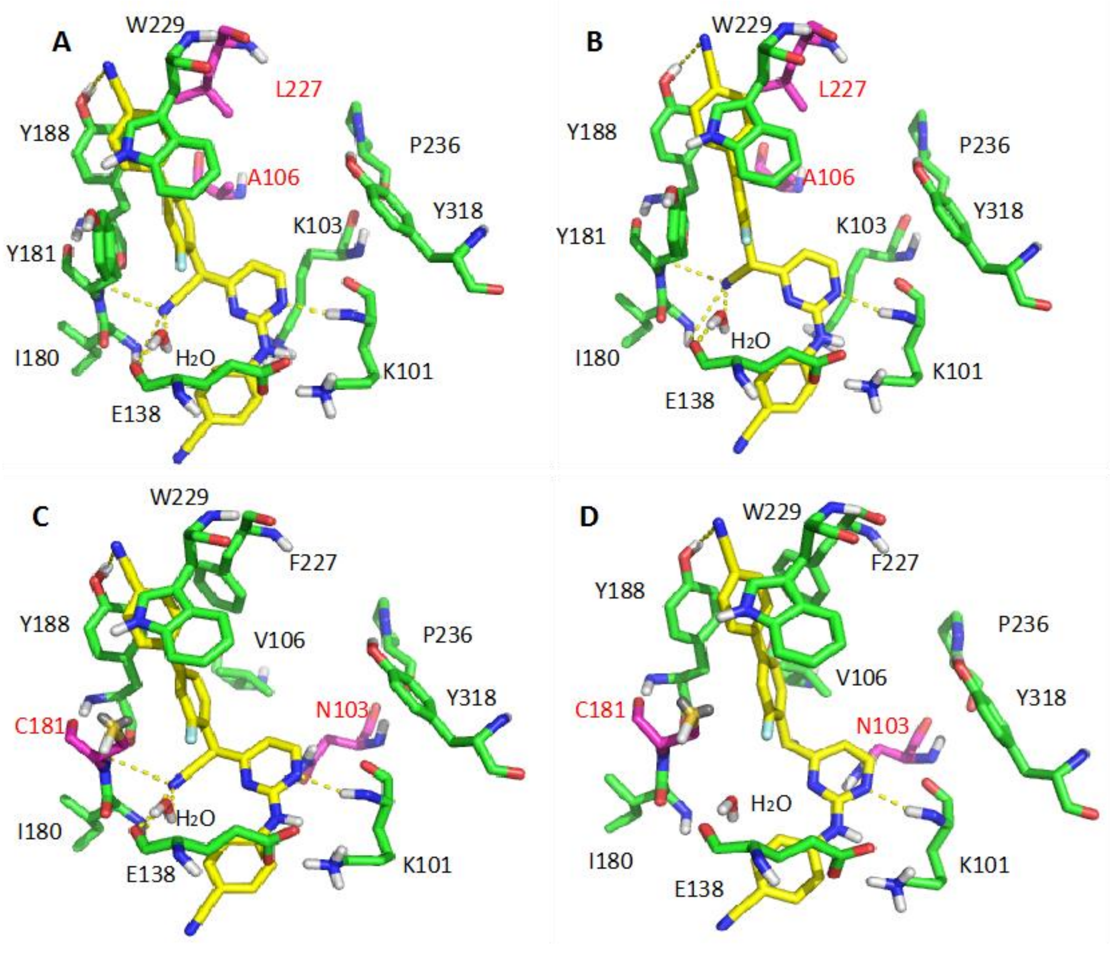

Figure S1 Predicted binding modes of **10p** with double mutant (F227L + V106A, K103N + Y181C) RT (PDB: 2ZD1). (A) F227L + V106A mutant RT with **10p**-*S*; (B) F227L + V106A mutant RT with **10p**-*R*; (C) K103N + Y181C mutant RT with **10p**-*S*; (D) K103N + Y181C mutant RT with **10p**-*R*.

^{1}H-NMR, ^{13}C-NMR and MS spectra of compounds

1. Spectra of compound 10a

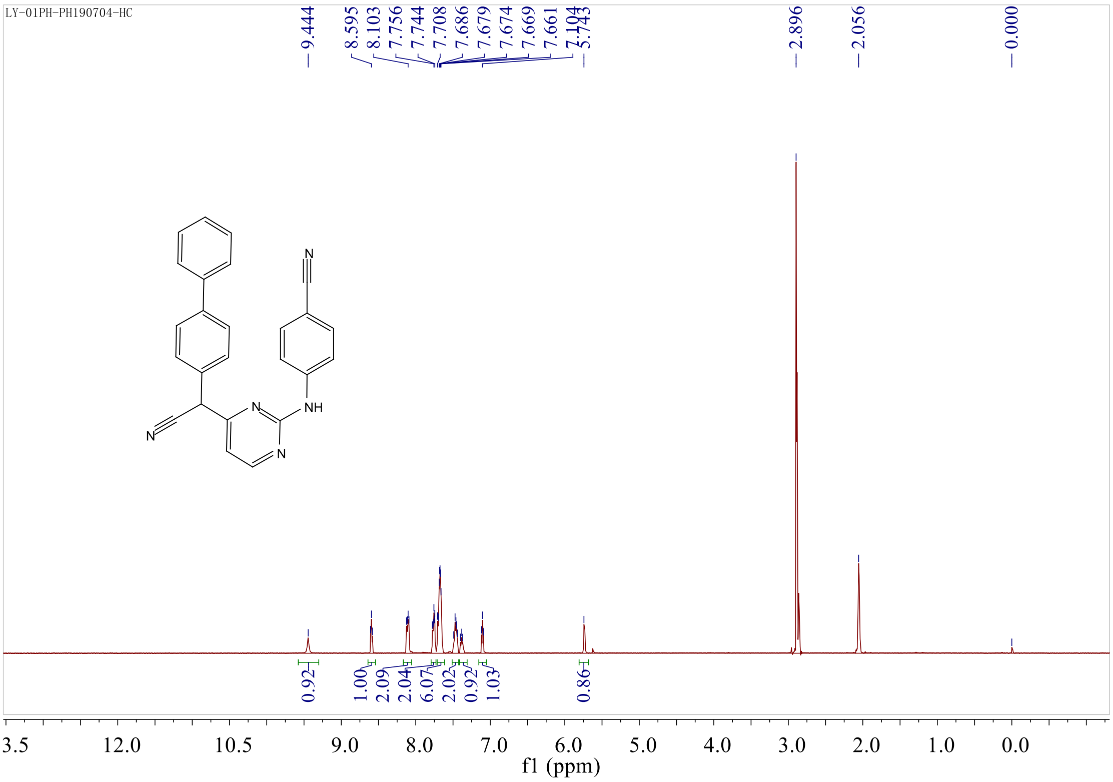

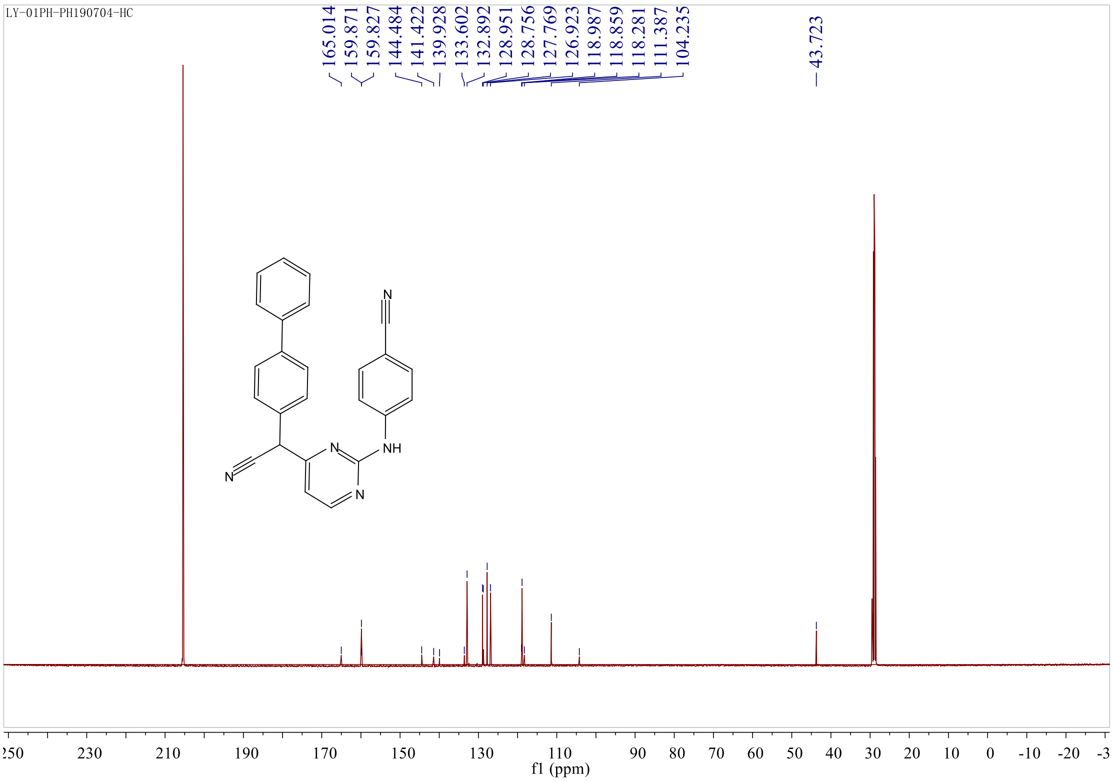

Display Report

Analysis Info

Analysis Name	D:\Data\data\2019\H-H_BB1_01_3080.d	Acquisition Date	1/7/2020 15:15:57 PM
Method	MS-Pos-10min.m	Operator	BDAL@DE
Sample Name	H-H	Instrument	compact 8255754.20127
Comment			

Acquisition Parameter

Source Type	ESI	Ion Polarity	Positive	Set Nebulizer	2.0 Bar
Focus	Active	Set Capillary	5000 V	Set Dry Heater	200 °C
Scan Begin	50 m/z	Set End Plate Offset	-500 V	Set Dry Gas	8.0 l/min
Scan End	1500 m/z	Set Charging Voltage	2000 V	Set Divert Valve	Waste
		Set Corona	0 nA	Set APCI Heater	0 °C

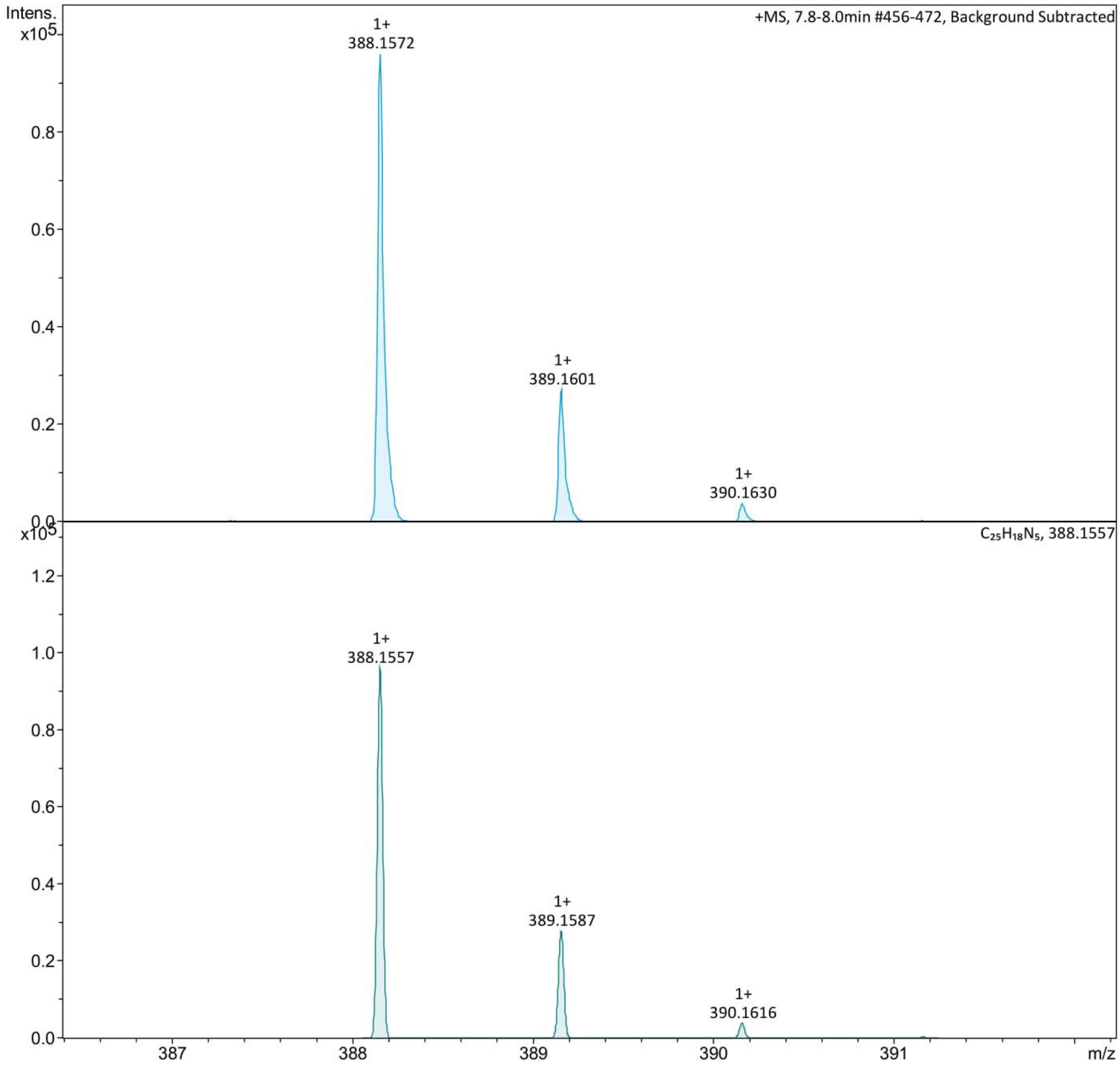

H-H_BB1_01_3080.d

Bruker Compass DataAnalysis 4.3 printed: 1/7/2020 16:13:25 PM by: BDAL@DE Page 1 of 1

2. Spectra of compound 10b

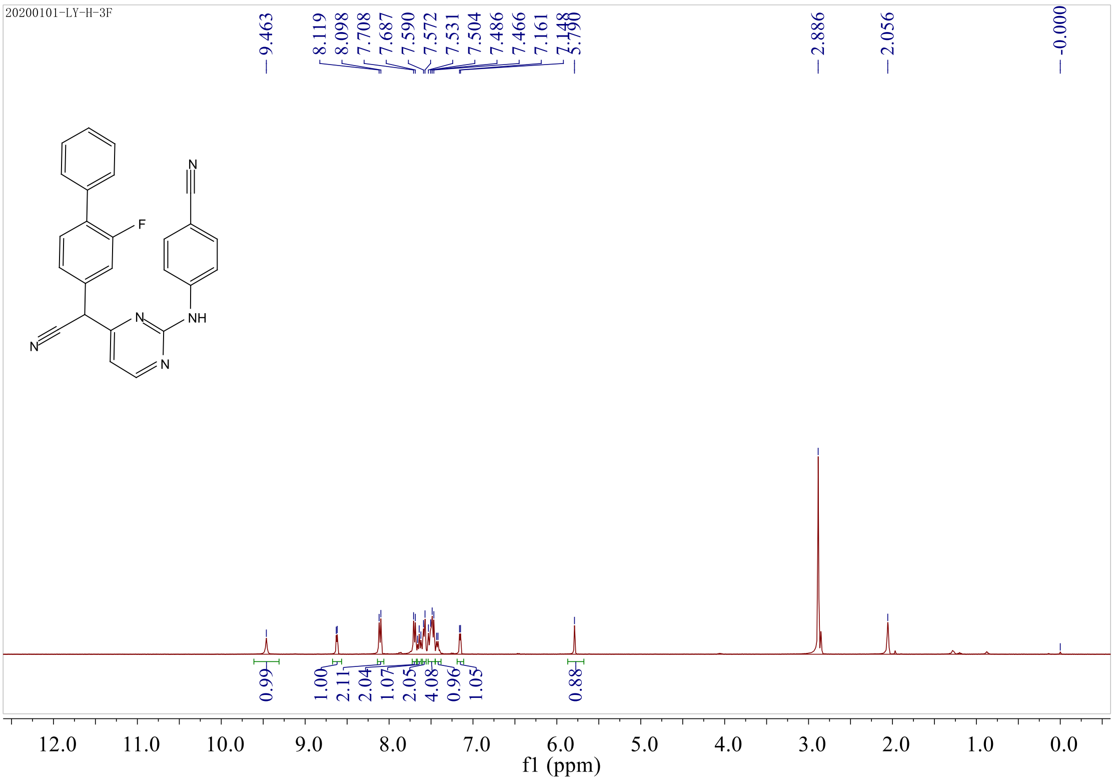

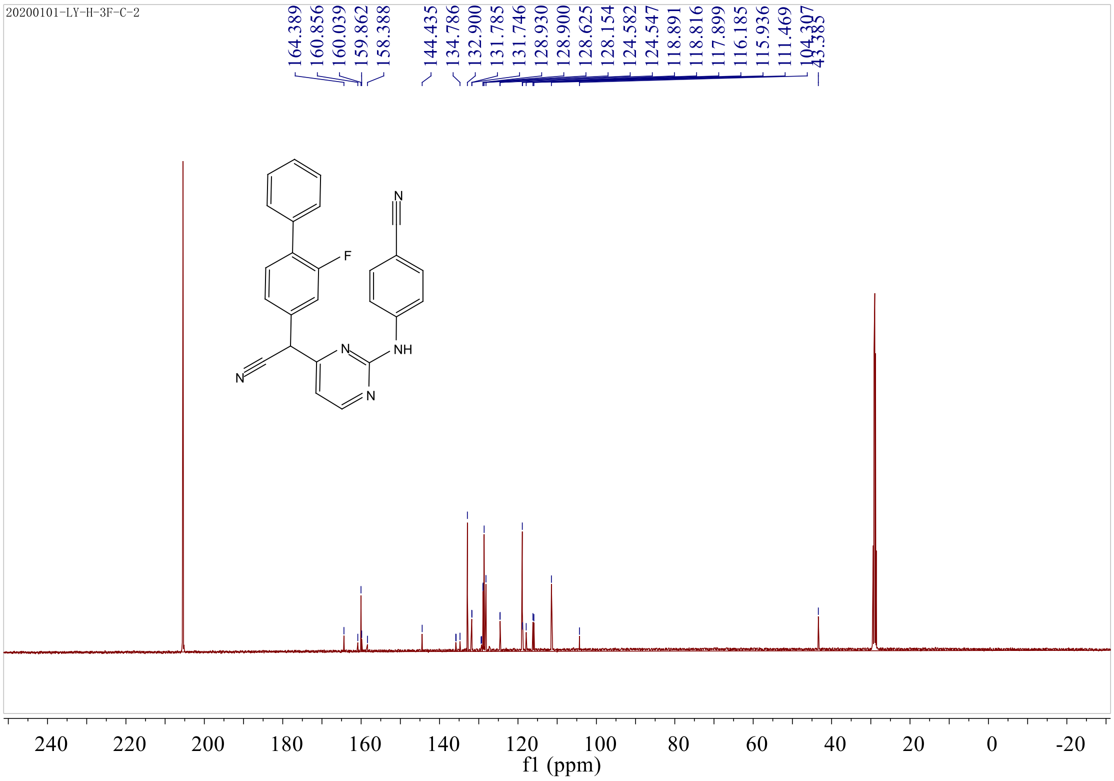

Display Report

Analysis Info

Analysis Name	D:\Data\data\2019\H-3F_BD5_01_2929.d	Acquisition Date	12/24/2019 11:08:34 AM
Method	MS-2MIN-NEG.m	Operator	BDAL@DE
Sample Name	H-3F	Instrument	compact 8255754.20127
Comment			

Acquisition Parameter

Source Type	ESI	Ion Polarity	Negative	Set Nebulizer	2.0 Bar
Focus	Active	Set Capillary	2800 V	Set Dry Heater	200 °C
Scan Begin	50 m/z	Set End Plate Offset	-500 V	Set Dry Gas	8.0 l/min
Scan End	1500 m/z	Set Charging Voltage	2000 V	Set Divert Valve	Waste
		Set Corona	0 nA	Set APCI Heater	0 °C

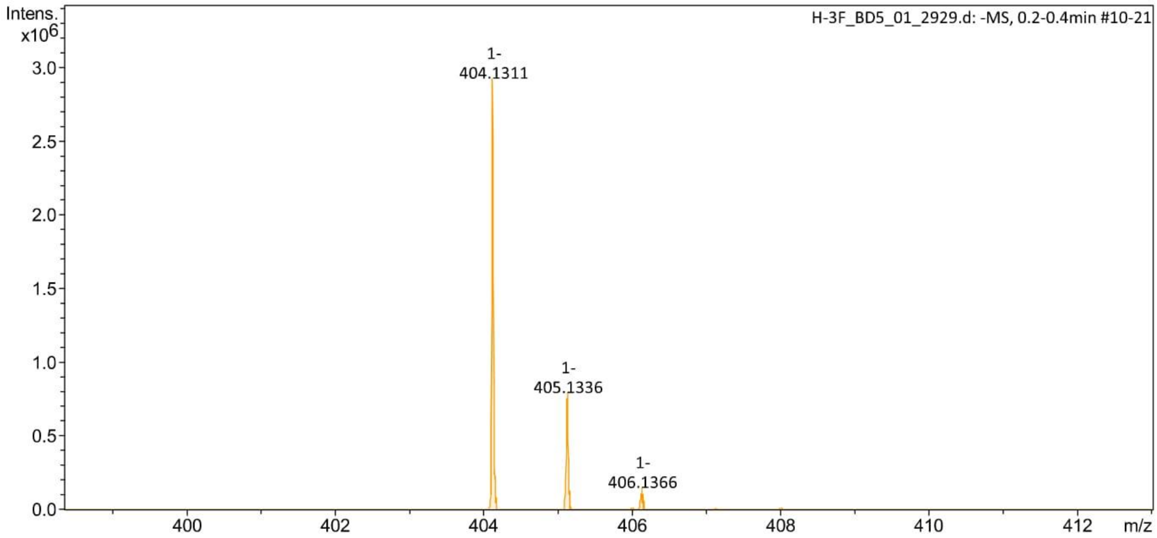

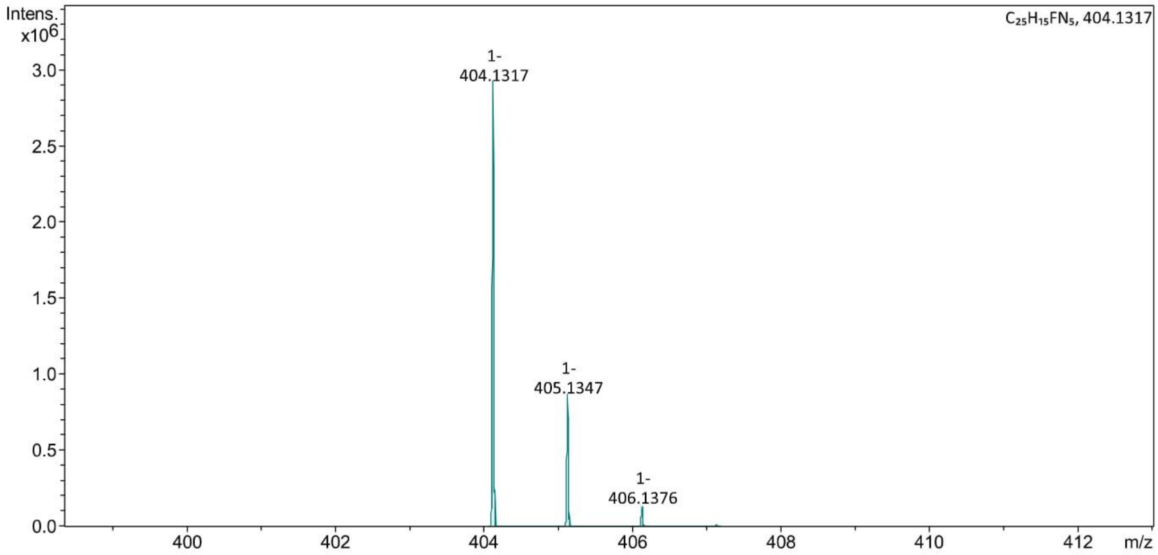

H-3F_BD5_01_2929.d

Bruker Compass DataAnalysis 4.3 printed: 12/24/2019 11:25:45 AM by: BDAL@DE Page 1 of 1

3. Spectra of compound 10c

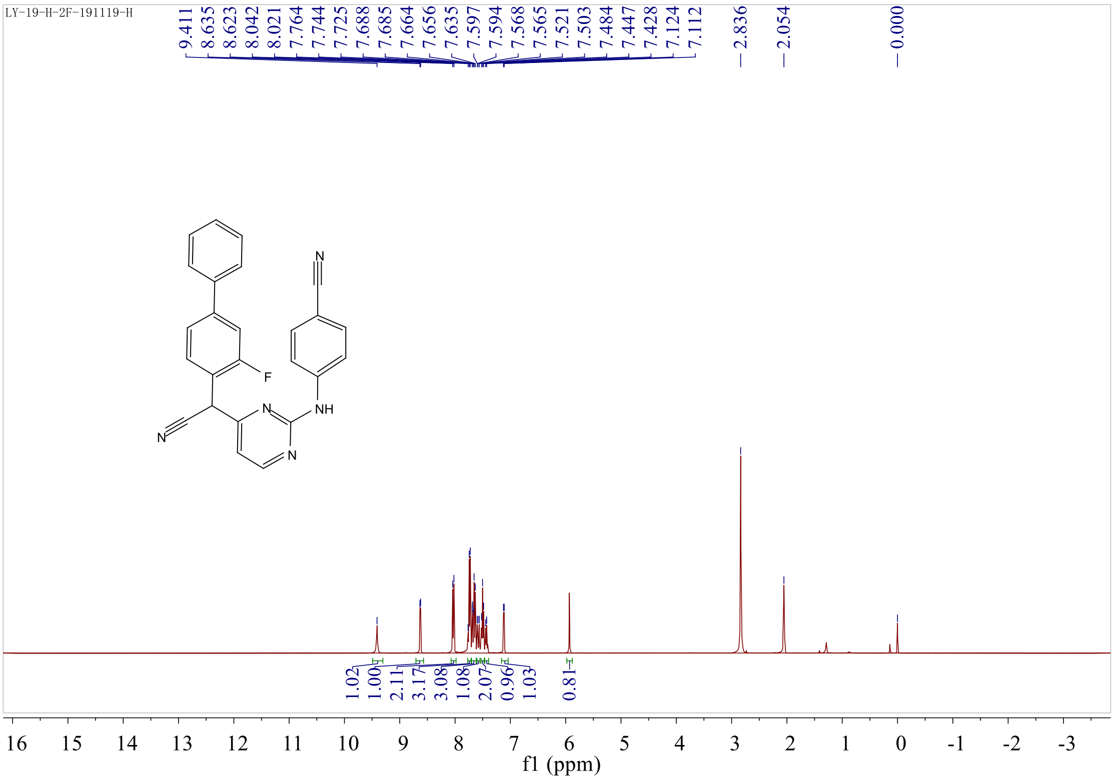

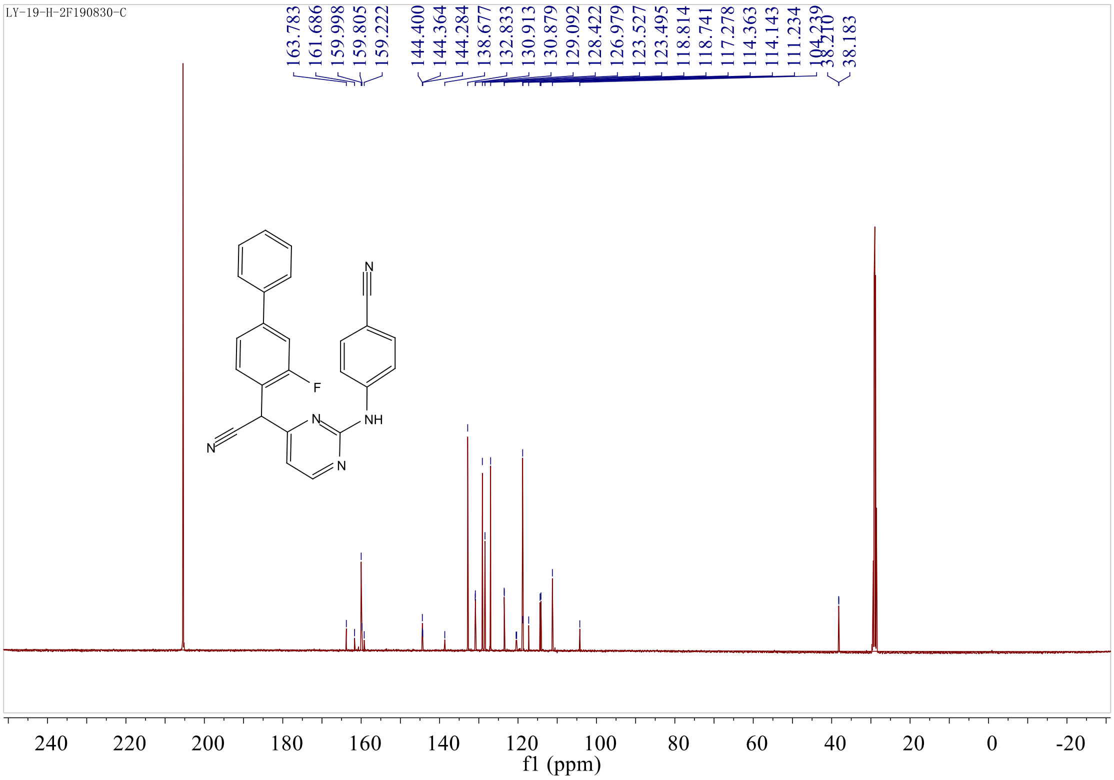

Display Report

Analysis Info

Analysis Name	D:\Data\data\2019\H-2F_BD6_01_2930.d	Acquisition Date	12/24/2019 11:11:19 AM
Method	MS-2MIN-NEG.m	Operator	BDAL@DE
Sample Name	H-2F	Instrument	compact 8255754.20127
Comment			

Acquisition Parameter

Source Type	ESI	Ion Polarity	Negative	Set Nebulizer	2.0 Bar
Focus	Active	Set Capillary	2800 V	Set Dry Heater	200 °C
Scan Begin	50 m/z	Set End Plate Offset	-500 V	Set Dry Gas	8.0 l/min
Scan End	1500 m/z	Set Charging Voltage	2000 V	Set Divert Valve	Waste
		Set Corona	0 nA	Set APCI Heater	0 °C

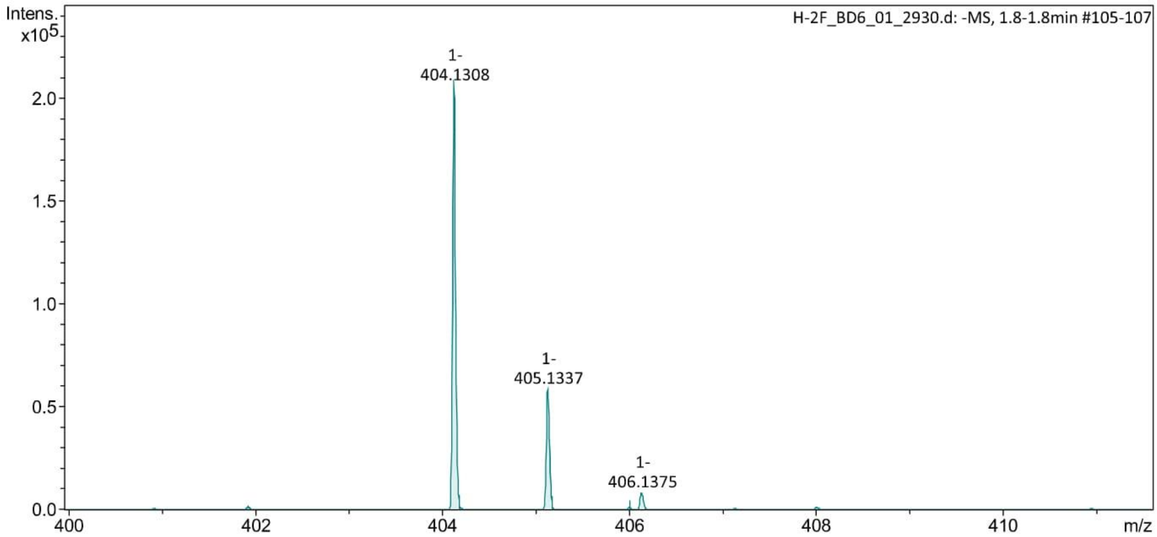

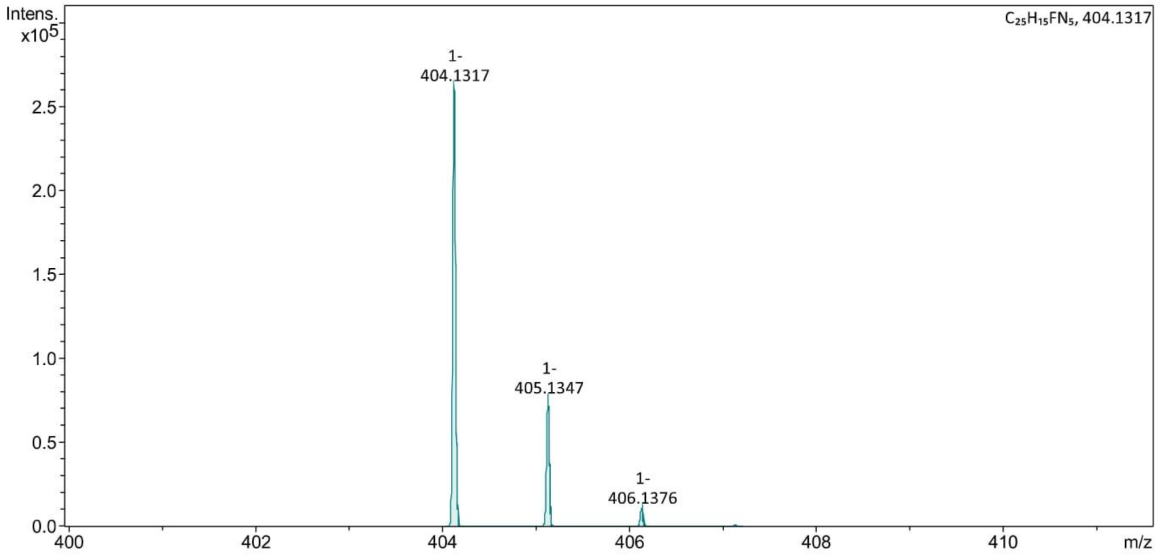

H-2F_BD6_01_2930.d

Bruker Compass DataAnalysis 4.3 printed: 12/24/2019 11:28:17 AM by: BDAL@DE Page 1 of 1

4. Spectra of compound 10d

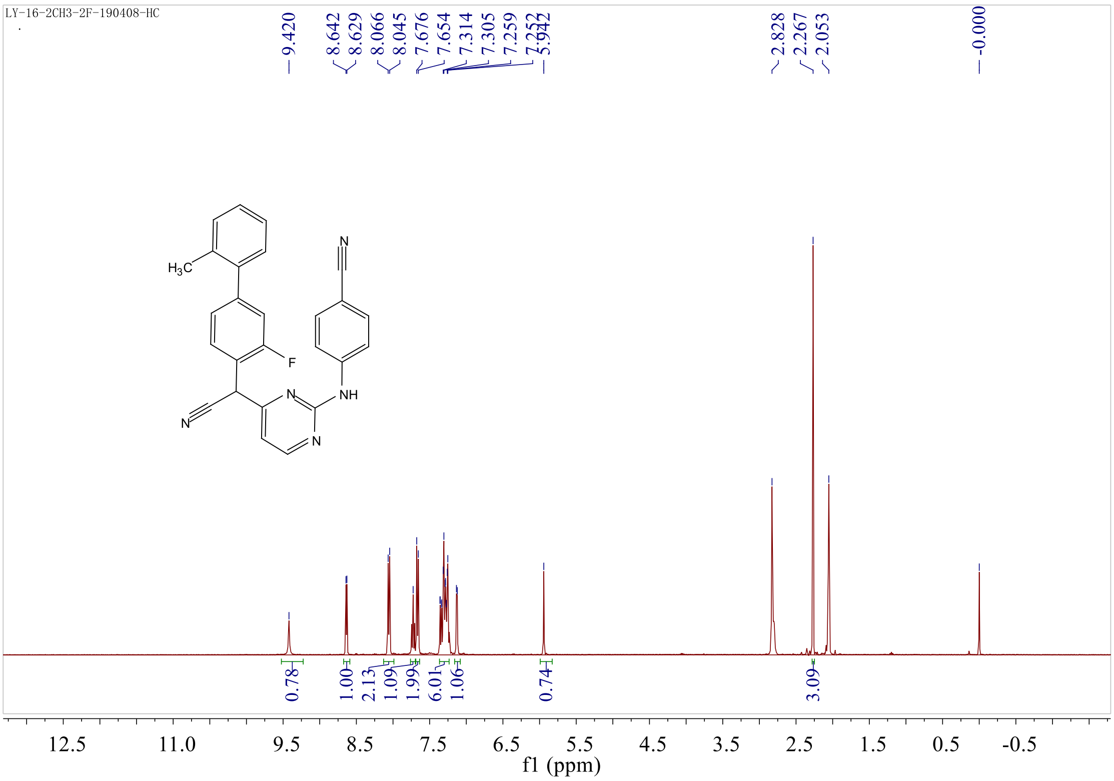

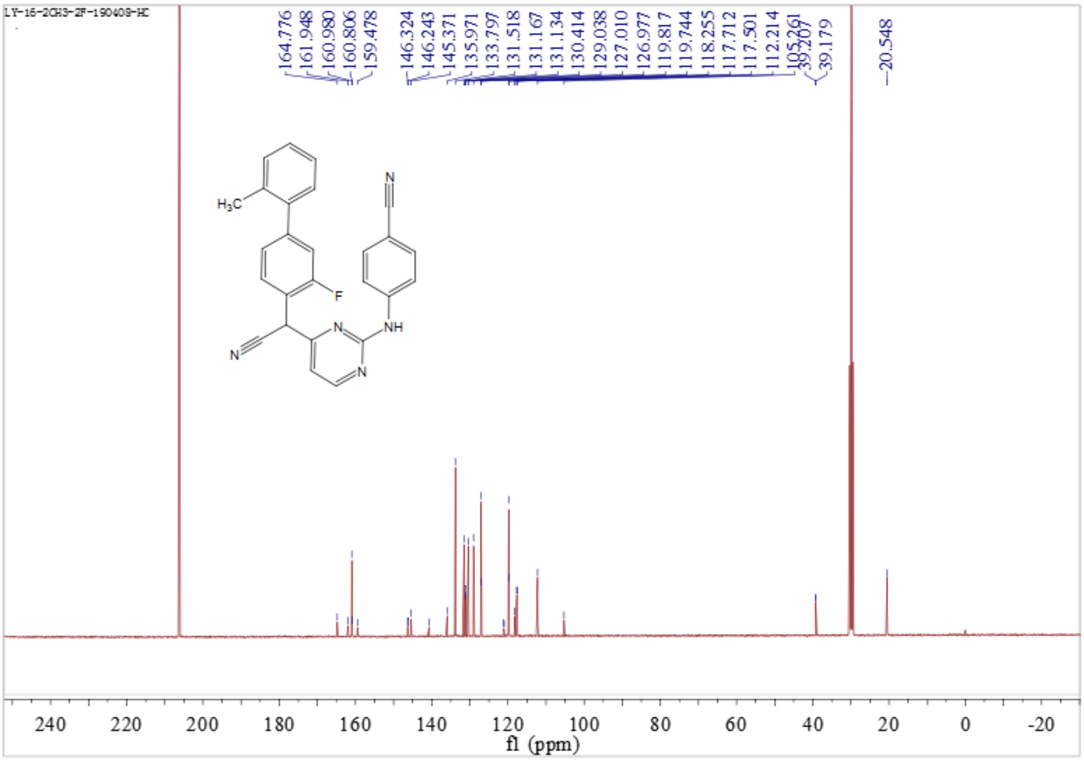

Display Report

Analysis Info

Analysis Name	D:\Data\data\2019\2CH3-2F_BD1_01_2925.d	Acquisition Date	12/24/2019 10:57:31 AM
Method	MS-2MIN-NEG.m	Operator	BDAL@DE
Sample Name	2CH3-2F	Instrument	compact 8255754.20127
Comment			

Acquisition Parameter

Source Type	ESI	Ion Polarity	Negative	Set Nebulizer	2.0 Bar
Focus	Active	Set Capillary	2800 V	Set Dry Heater	200 °C
Scan Begin	50 m/z	Set End Plate Offset	-500 V	Set Dry Gas	8.0 l/min
Scan End	1500 m/z	Set Charging Voltage	2000 V	Set Divert Valve	Waste
		Set Corona	0 nA	Set APCI Heater	0 °C

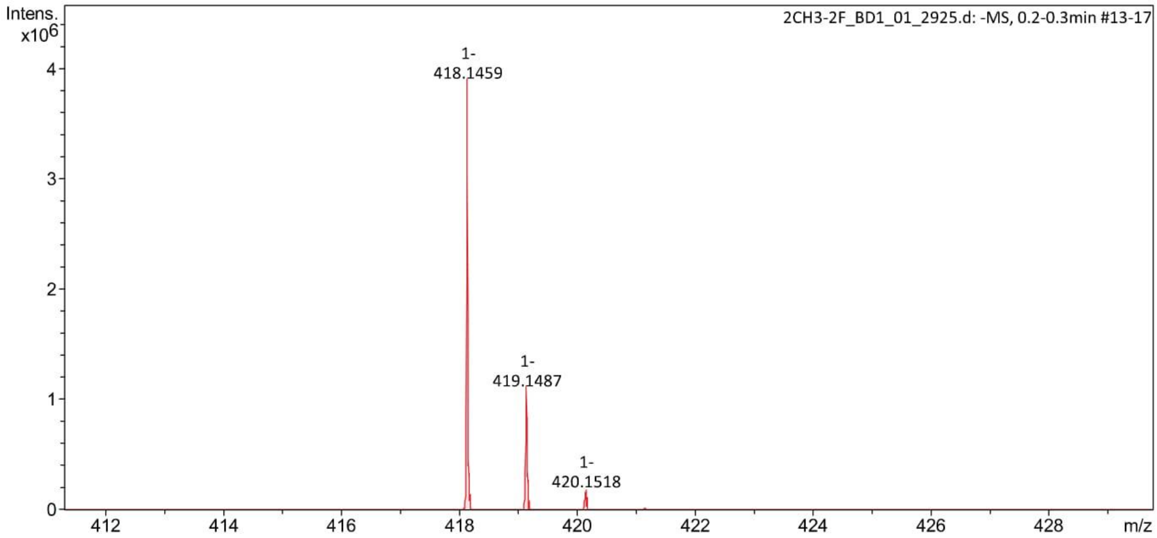

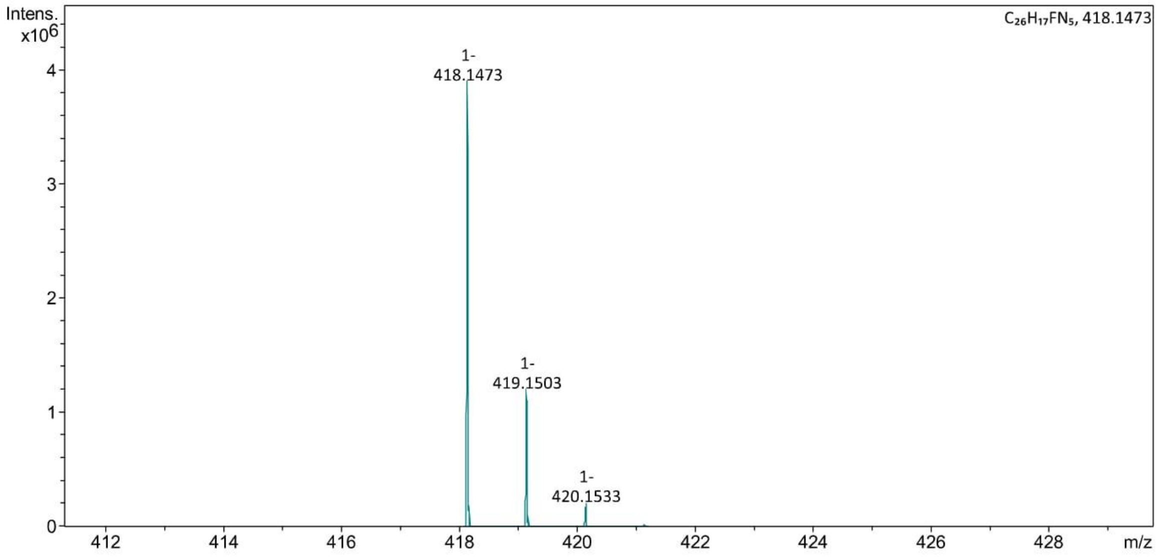

2CH3-2F_BD1_01_2925.d

Bruker Compass DataAnalysis 4.3 printed: 12/24/2019 11:18:13 AM by: BDAL@DE Page 1 of 1

5. Spectra of compound 10e

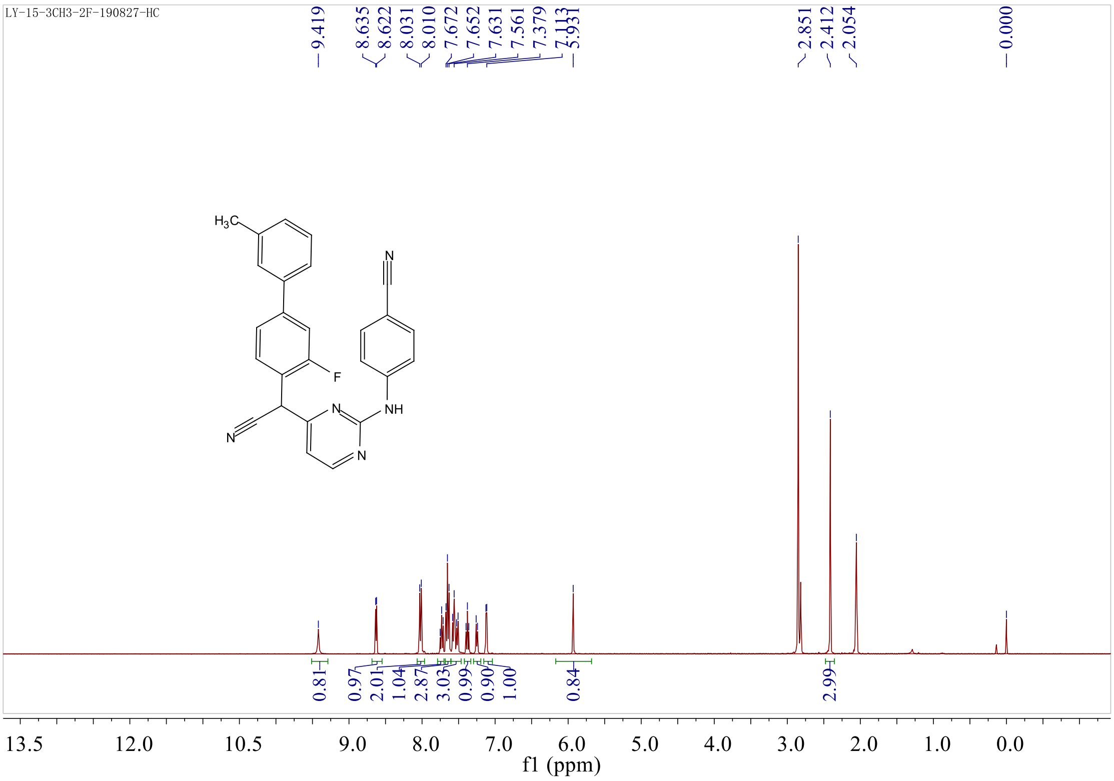

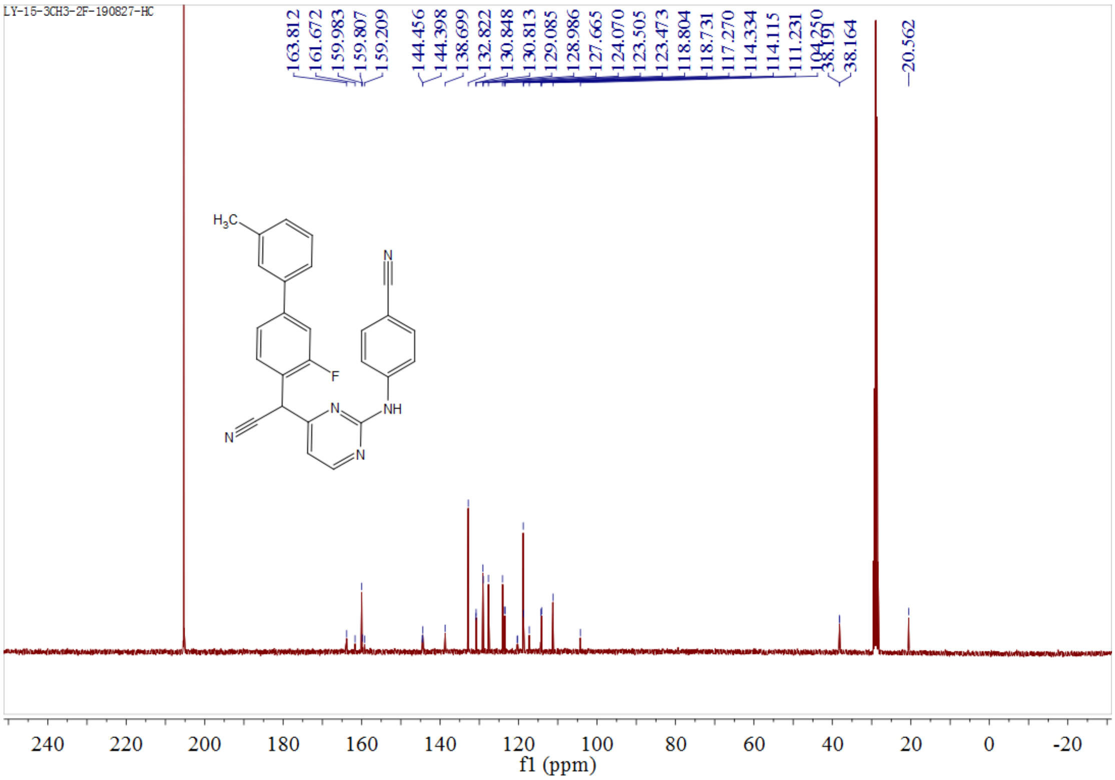

Display Report

Analysis Info

Analysis Name	D:\Data\data\2019\3CH32F_RC2_01_2873.d	Acquisition Date	12/18/2019 17:39:00 PM
Method	MS-2MIN-NEG.m	Operator	BDAL@DE
Sample Name	3CH32F	Instrument	compact 8255754.20127
Comment			

Acquisition Parameter

Source Type	ESI	Ion Polarity	Negative	Set Nebulizer	2.0 Bar
Focus	Active	Set Capillary	2800 V	Set Dry Heater	200 °C
Scan Begin	50 m/z	Set End Plate Offset	-500 V	Set Dry Gas	8.0 l/min
Scan End	1500 m/z	Set Charging Voltage	2000 V	Set Divert Valve	Waste
		Set Corona	0 nA	Set APCI Heater	0 °C

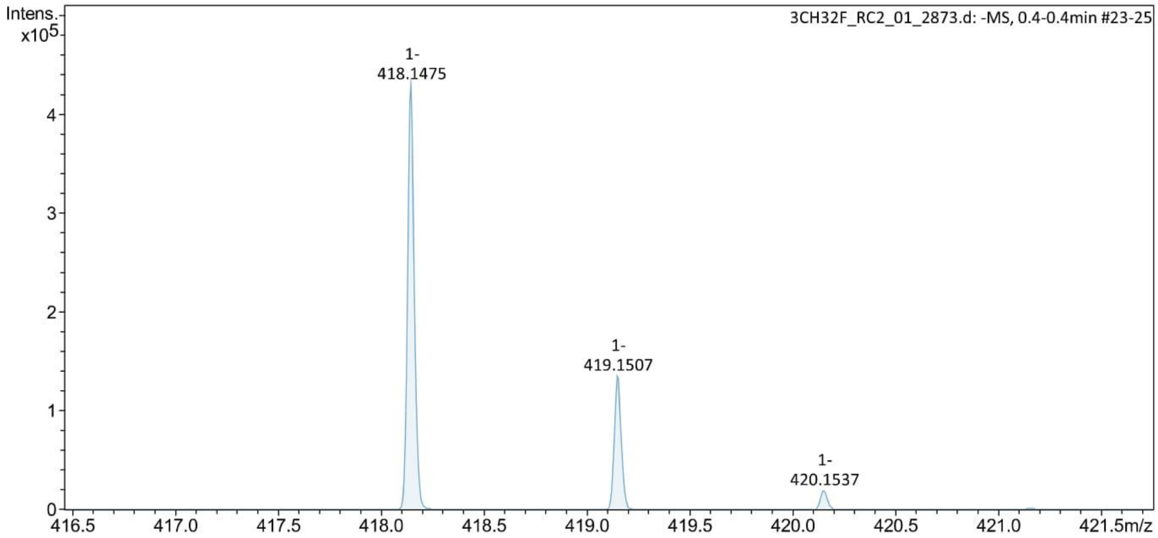

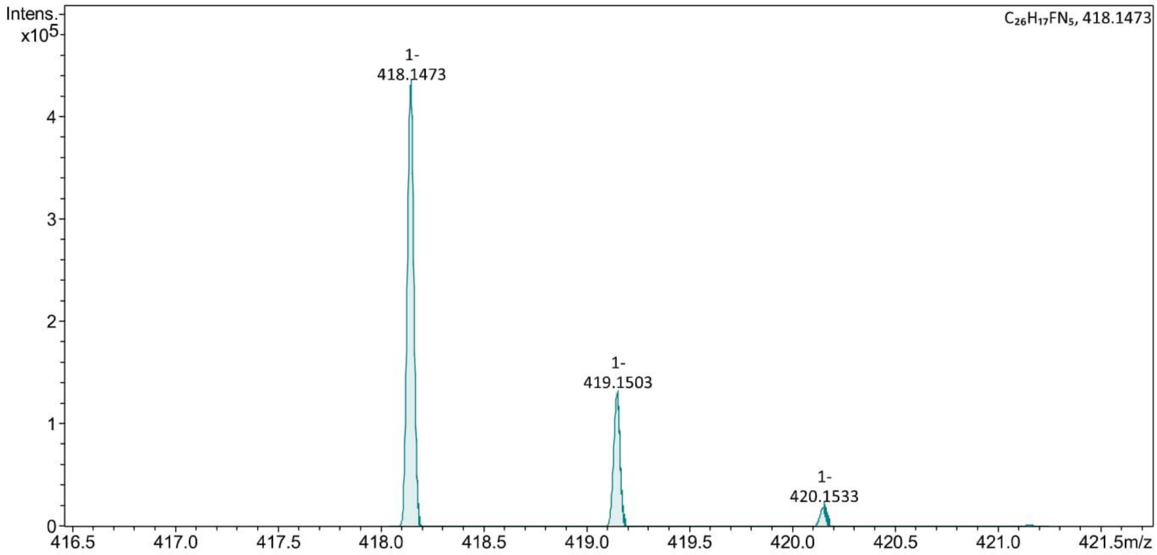

3CH32F_RC2_01_2873.d

Bruker Compass DataAnalysis 4.3 printed: 12/19/2019 13:41:53 PM by: BDAL@DE Page 1 of 1

6. Spectra of compound 10 f

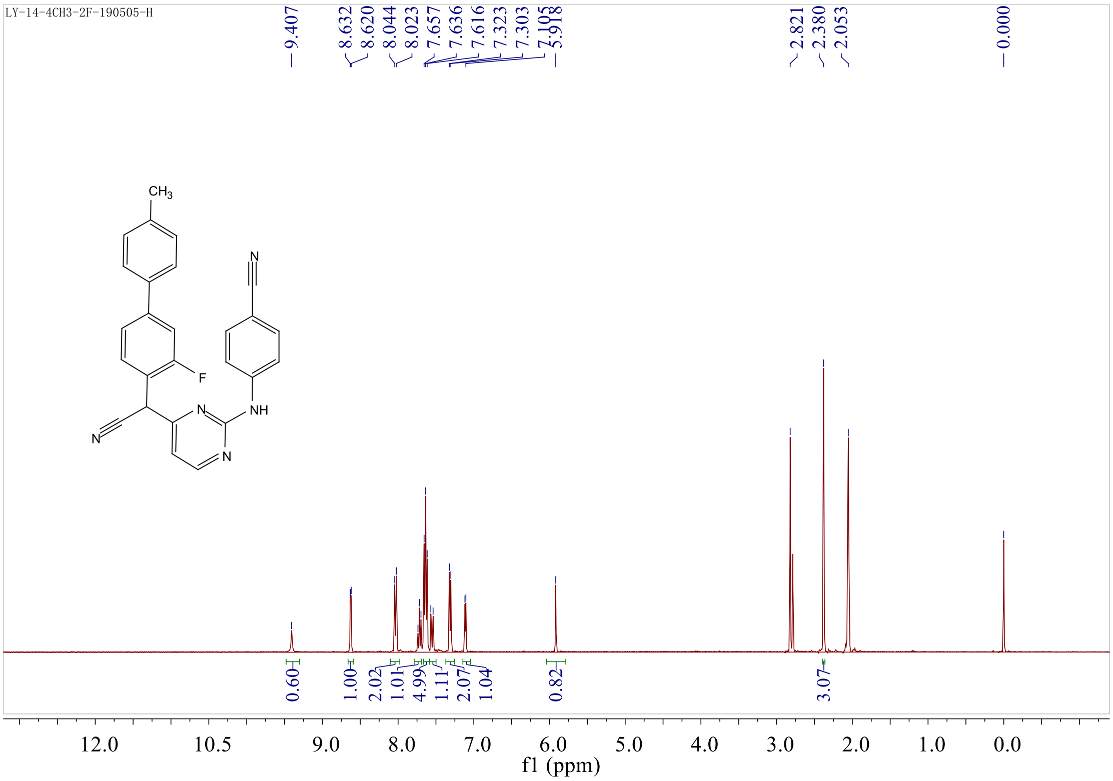

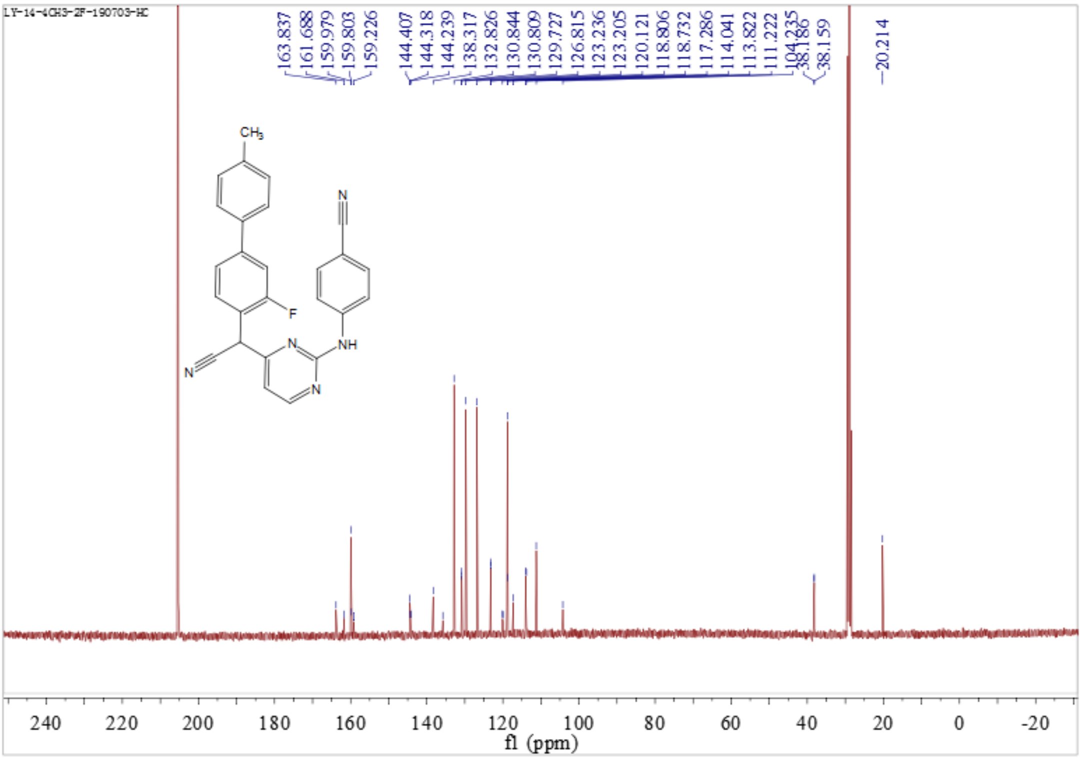

Display Report

Analysis Info

Analysis Name	D:\Data\data\2019\4CH3-2F_RD3_01_2864.d	Acquisition Date	12/18/2019 17:14:09 PM
Method	MS-2MIN-NEG.m	Operator	BDAL@DE
Sample Name	4CH3-2F	Instrument	compact 8255754.20127
Comment			

Acquisition Parameter

Source Type	ESI	Ion Polarity	Negative	Set Nebulizer	2.0 Bar
Focus	Active	Set Capillary	2800 V	Set Dry Heater	200 °C
Scan Begin	50 m/z	Set End Plate Offset	-500 V	Set Dry Gas	8.0 l/min
Scan End	1500 m/z	Set Charging Voltage	2000 V	Set Divert Valve	Waste
		Set Corona	0 nA	Set APCI Heater	0 °C

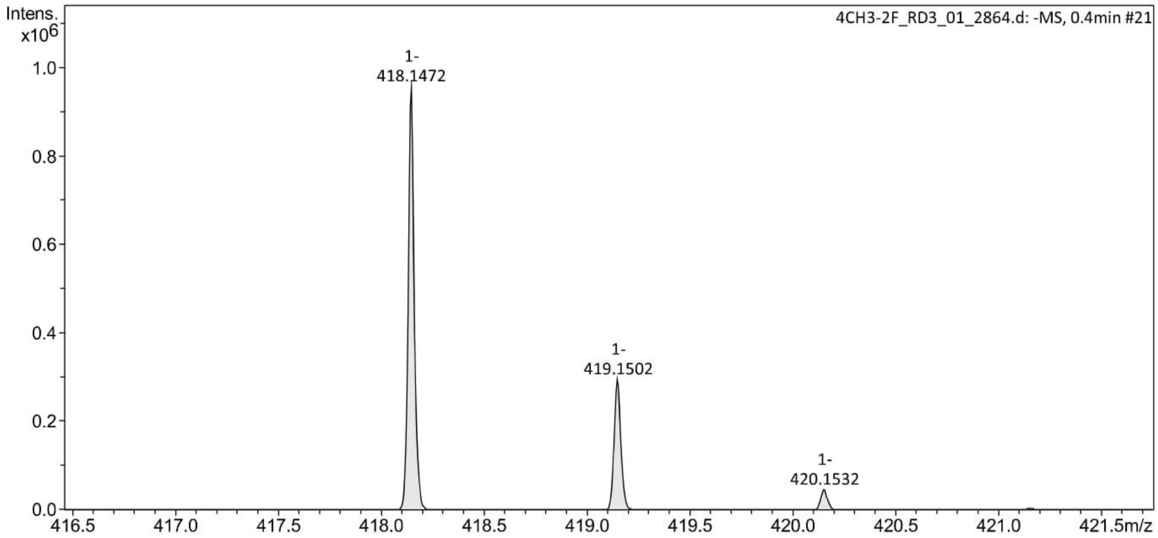

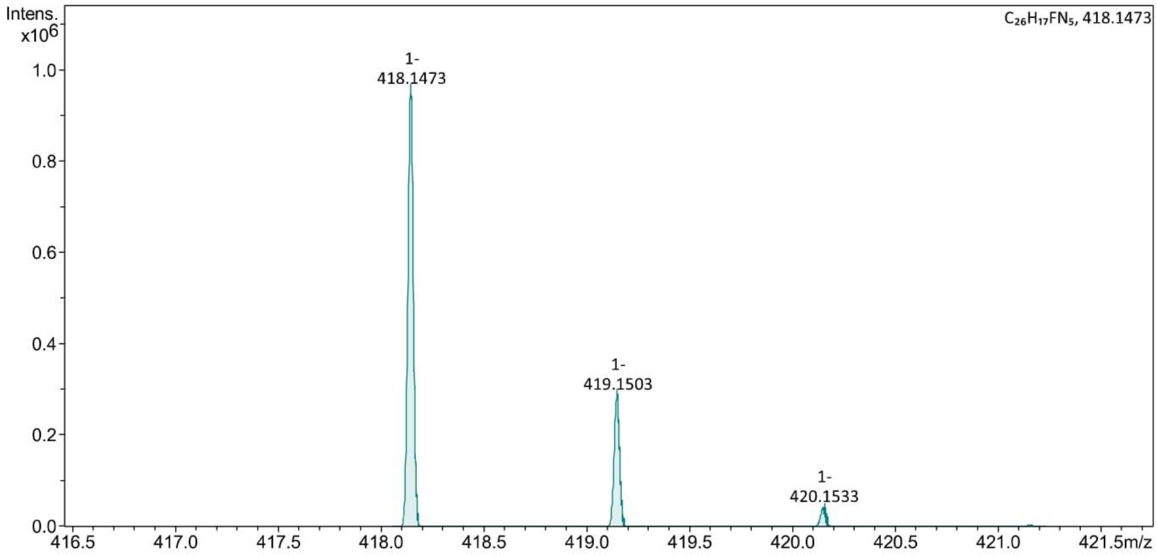

4CH3-2F_RD3_01_2864.d

Bruker Compass DataAnalysis 4.3 printed: 12/19/2019 13:39:46 PM by: BDAL@DE Page 1 of 1

7. Spectra of compound 10g

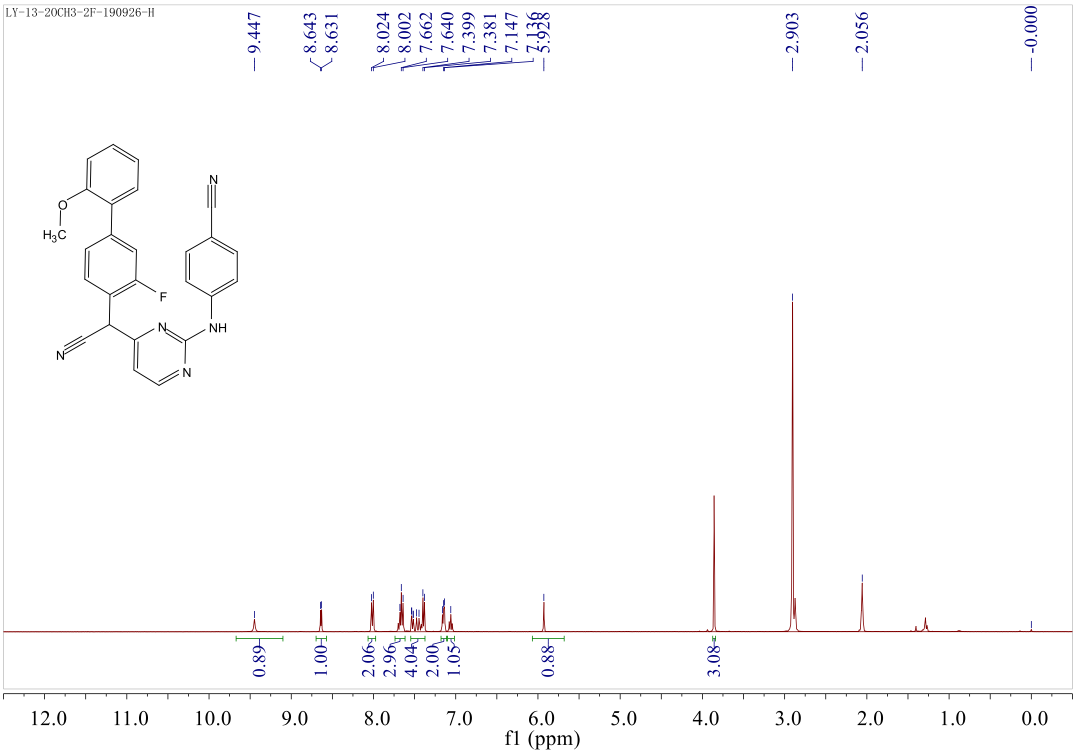

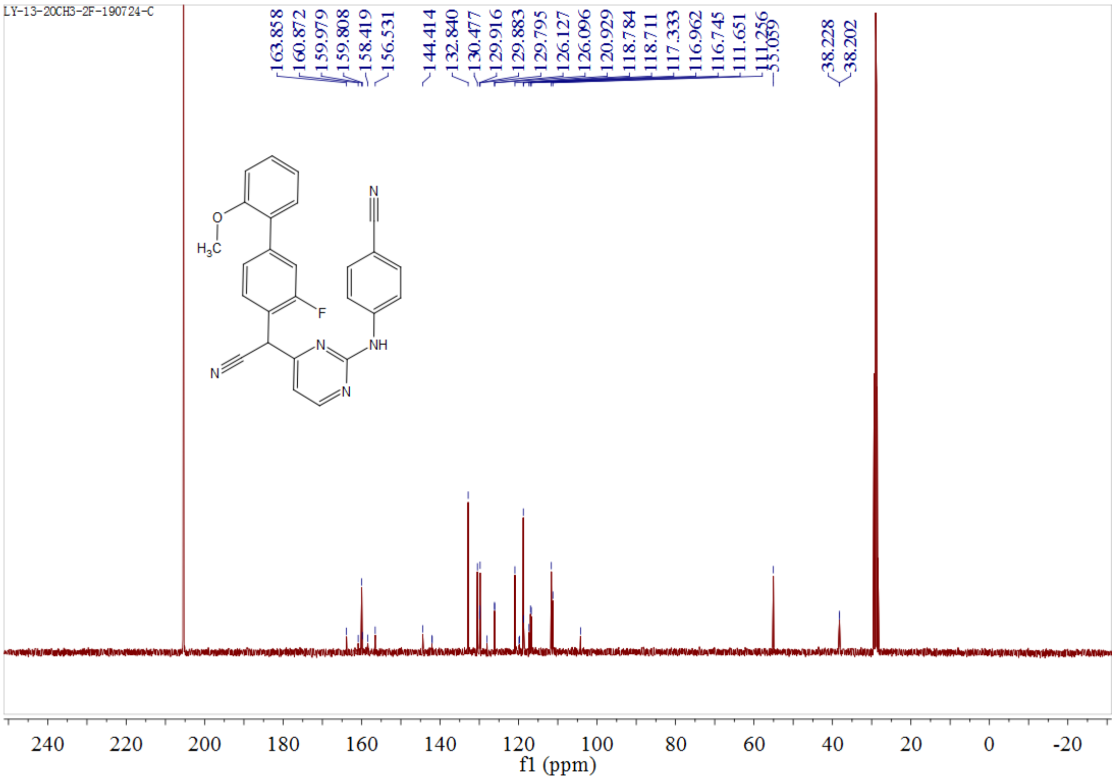

Display Report

Analysis Info

Analysis Name	D:\Data\data\2019\2OCH3-2F_RC1_01_2874.d	Acquisition Date	12/18/2019 17:41:46 PM
Method	MS-2MIN-NEG.m	Operator	BDAL@DE
Sample Name	2OCH3-2F	Instrument	compact 8255754.20127
Comment			

Acquisition Parameter

Source Type	ESI	Ion Polarity	Negative	Set Nebulizer	2.0 Bar
Focus	Active	Set Capillary	2800 V	Set Dry Heater	200 °C
Scan Begin	50 m/z	Set End Plate Offset	-500 V	Set Dry Gas	8.0 l/min
Scan End	1500 m/z	Set Charging Voltage	2000 V	Set Divert Valve	Waste
		Set Corona	0 nA	Set APCI Heater	0 °C

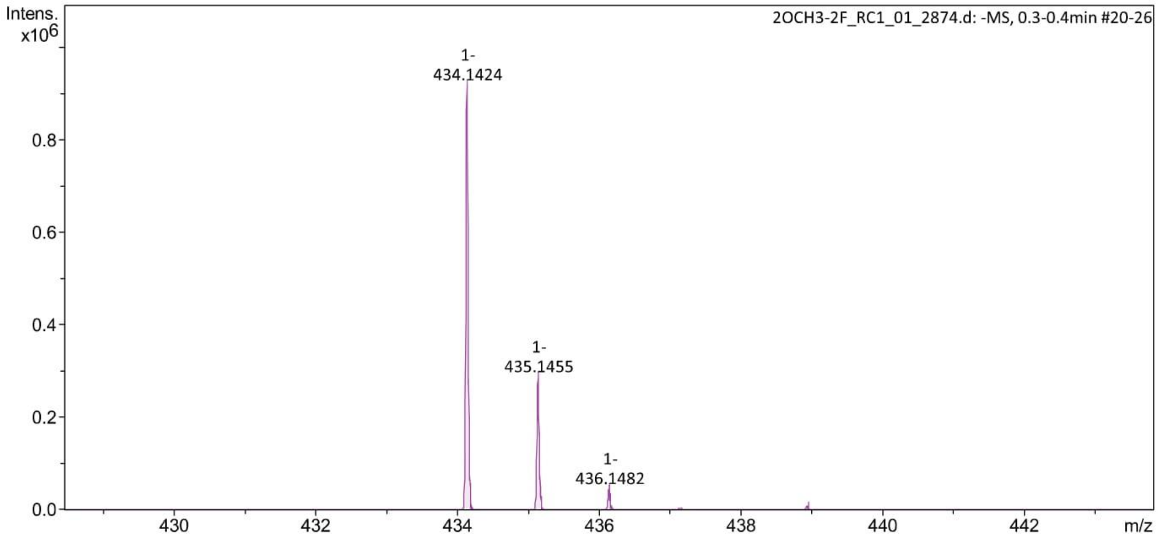

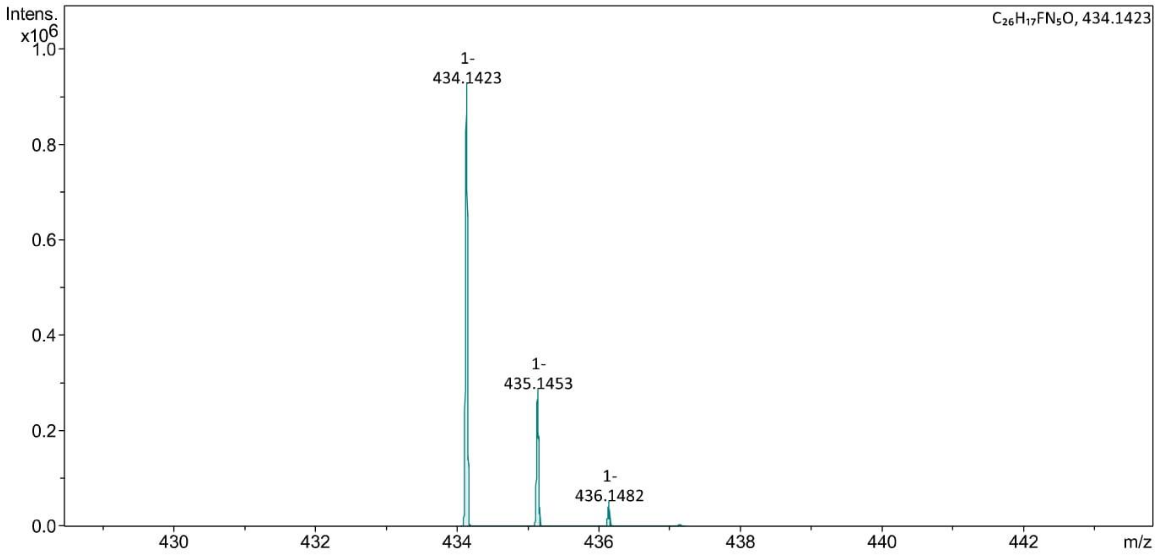

2OCH3-2F_RC1_01_2874.d

Bruker Compass DataAnalysis 4.3 printed: 12/19/2019 12:18:50 PM by: BDAL@DE Page 1 of 1

8. Spectra of compound 10h

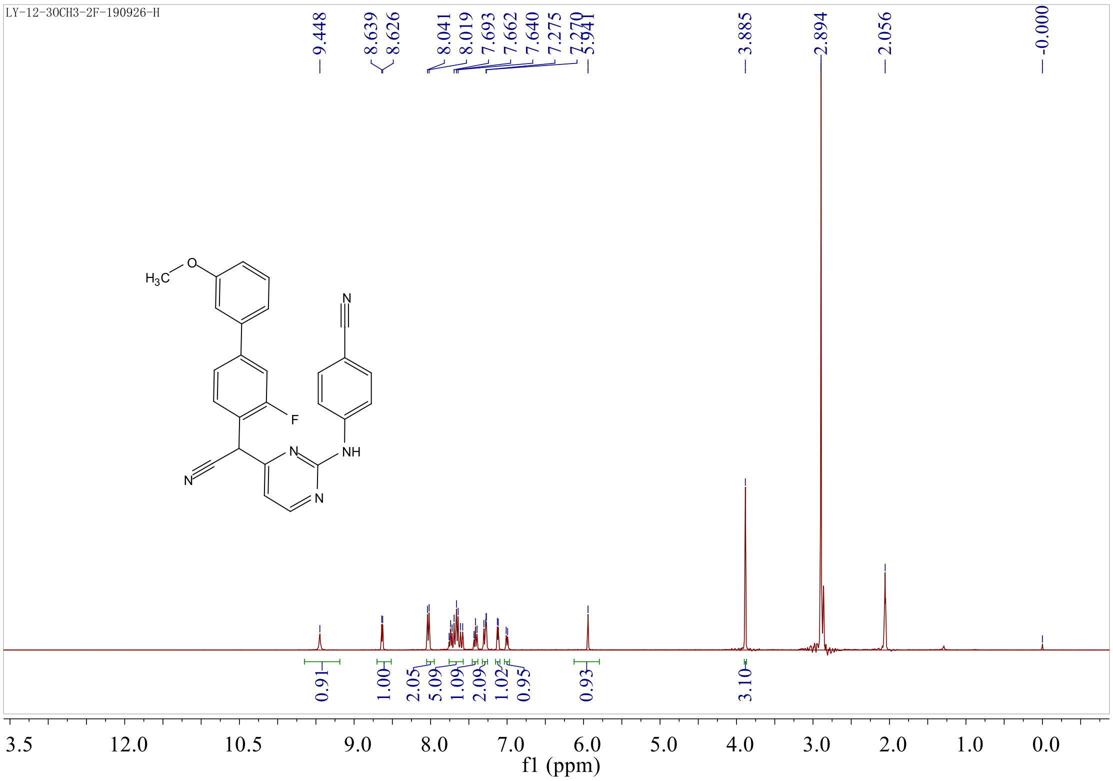

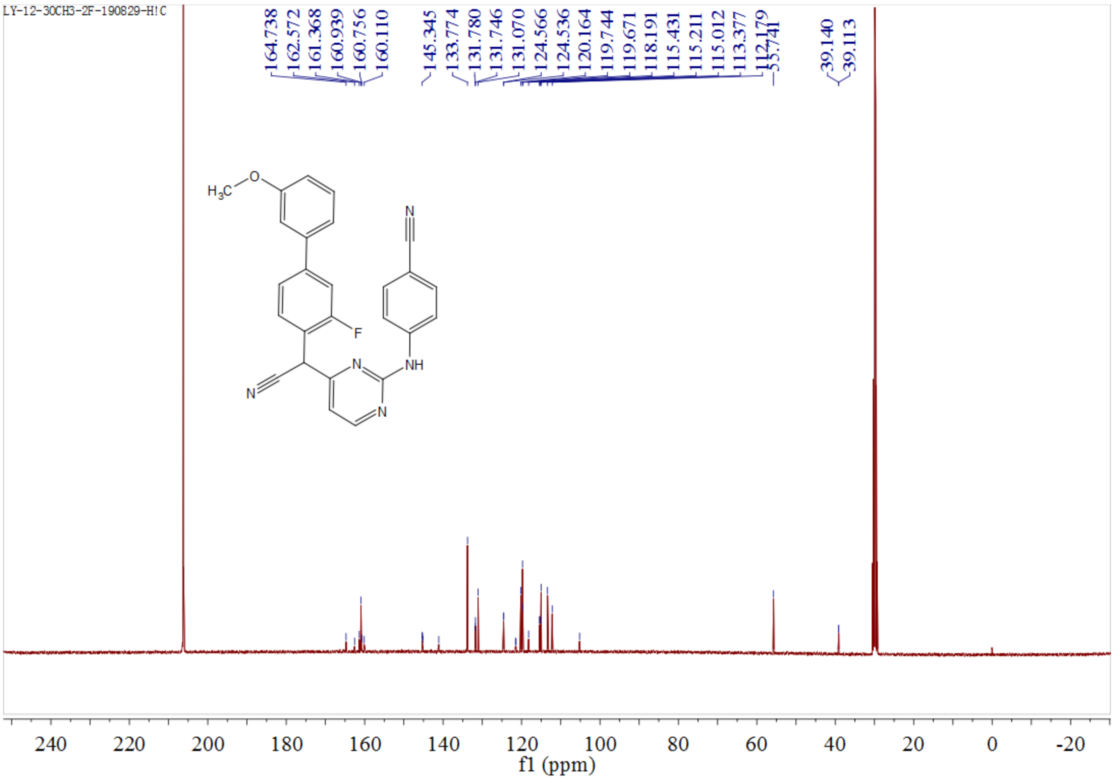

Display Report

Analysis Info

Analysis Name	D:\Data\data\2019\3OCH3-2F_RE8_01_2851.d	Acquisition Date	12/18/2019 16:38:14 PM
Method	MS-2MIN-NEG.m	Operator	BDAL@DE
Sample Name	3OCH3-2F	Instrument	compact 8255754.20127
Comment			

Acquisition Parameter

Source Type	ESI	Ion Polarity	Negative	Set Nebulizer	2.0 Bar
Focus	Active	Set Capillary	2800 V	Set Dry Heater	200 °C
Scan Begin	50 m/z	Set End Plate Offset	-500 V	Set Dry Gas	8.0 l/min
Scan End	1500 m/z	Set Charging Voltage	2000 V	Set Divert Valve	Waste
		Set Corona	0 nA	Set APCI Heater	0 °C

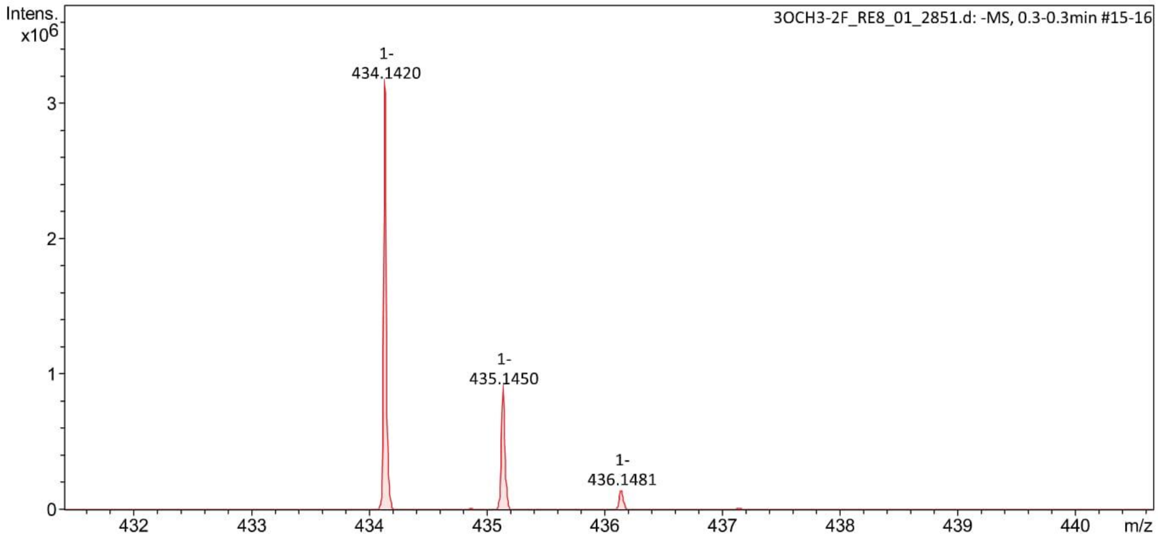

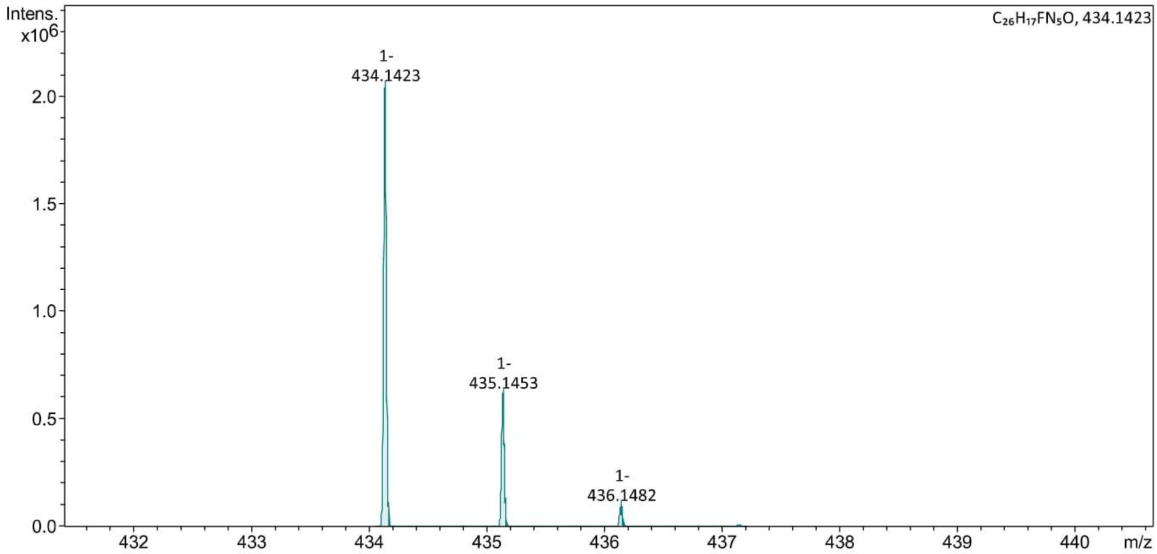

3OCH3-2F_RE8_01_2851.d

Bruker Compass DataAnalysis 4.3 printed: 12/19/2019 12:35:06 PM by: BDAL@DE Page 1 of 1

9. Spectra of compound 10i

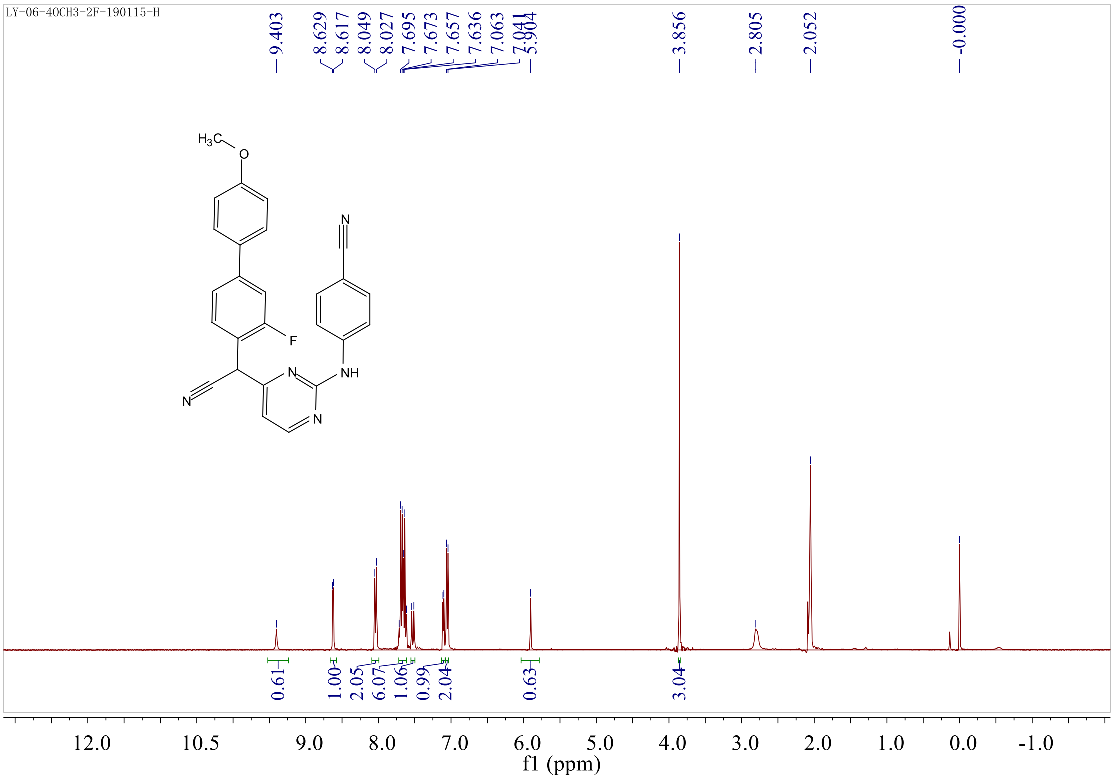

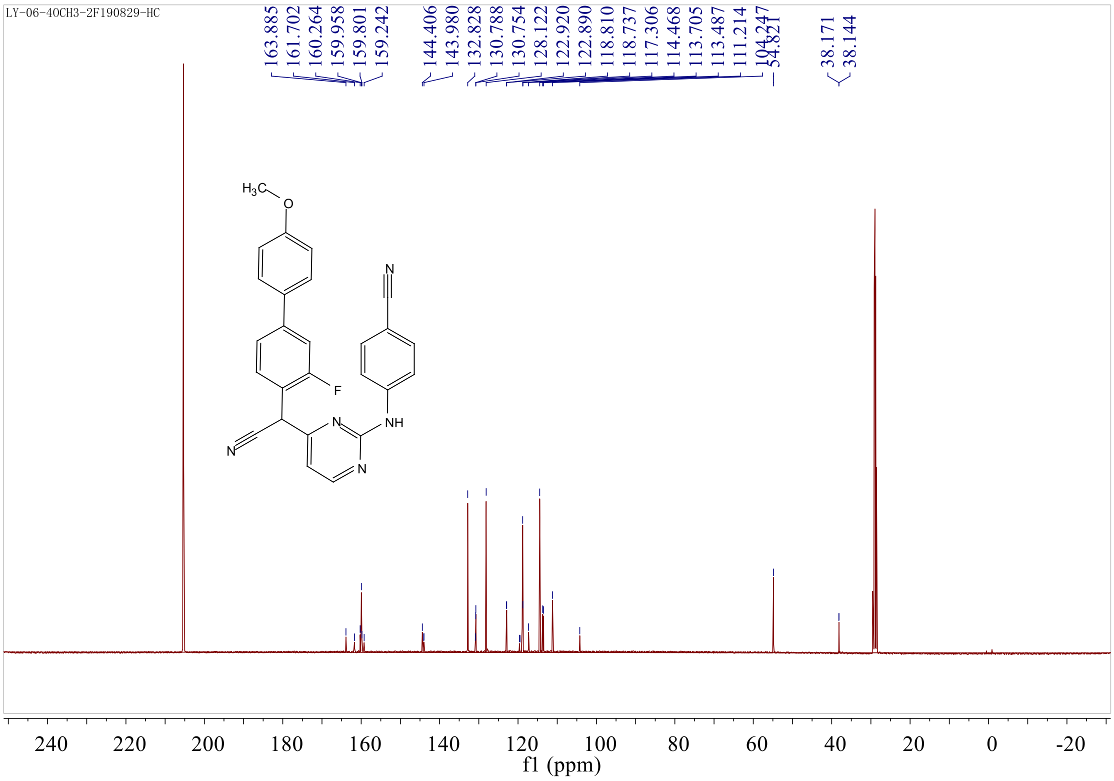

Display Report

Analysis Info

Analysis Name	D:\Data\data\2019\4OCH3-2F_GA3_01_2903.d	Acquisition Date	12/18/2019 19:02:40 PM	
Method	MS-2MIN-NEG.m	Operator	BDAL@DE	
Sample Name	4OCH3-2F	Instrument	compact	8255754.20127
Comment				

Acquisition Parameter

Source Type	ESI	Ion Polarity	Negative	Set Nebulizer	2.0 Bar
Focus	Active	Set Capillary	2800 V	Set Dry Heater	200 °C
Scan Begin	50 m/z	Set End Plate Offset	-500 V	Set Dry Gas	8.0 l/min
Scan End	1500 m/z	Set Charging Voltage	2000 V	Set Divert Valve	Waste
		Set Corona	0 nA	Set APCI Heater	0 °C

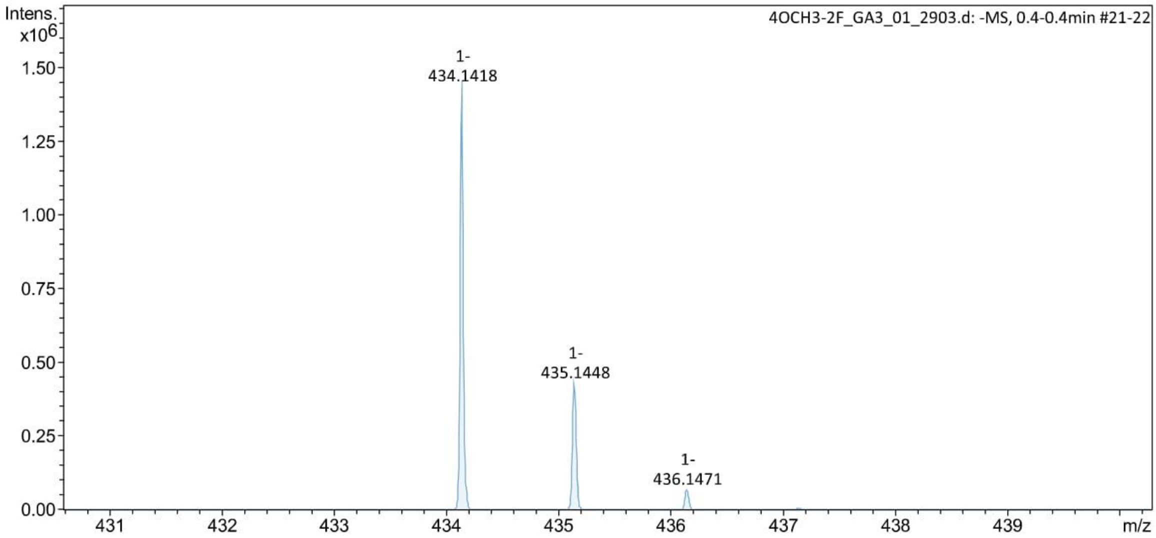

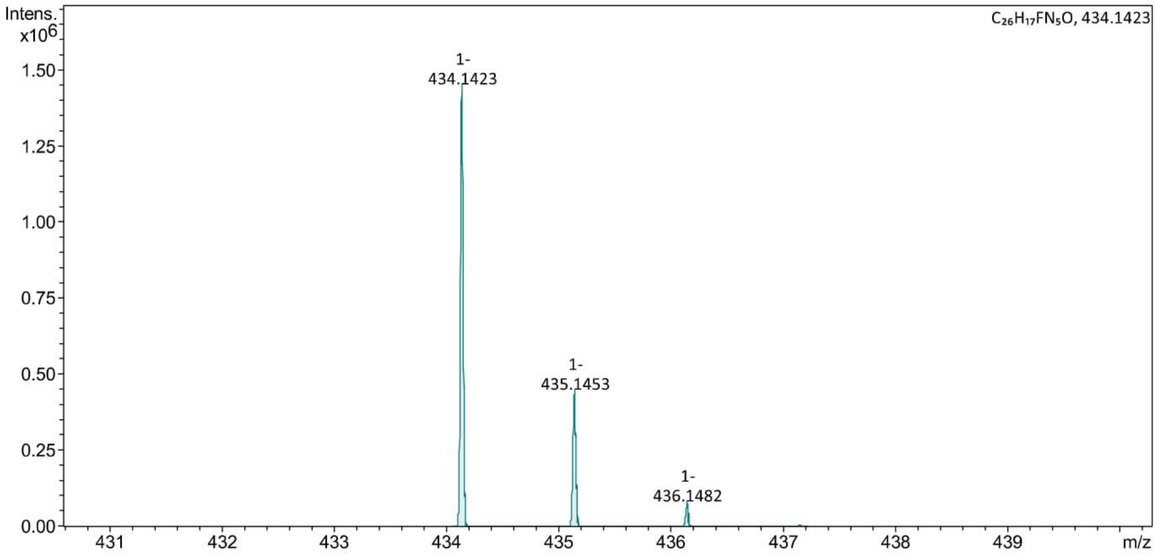

4OCH3-2F_GA3_01_2903.d

Bruker Compass DataAnalysis 4.3 printed: 12/19/2019 11:52:23 AM by: BDAL@DE Page 1 of 1

10. Spectra of compound 10j

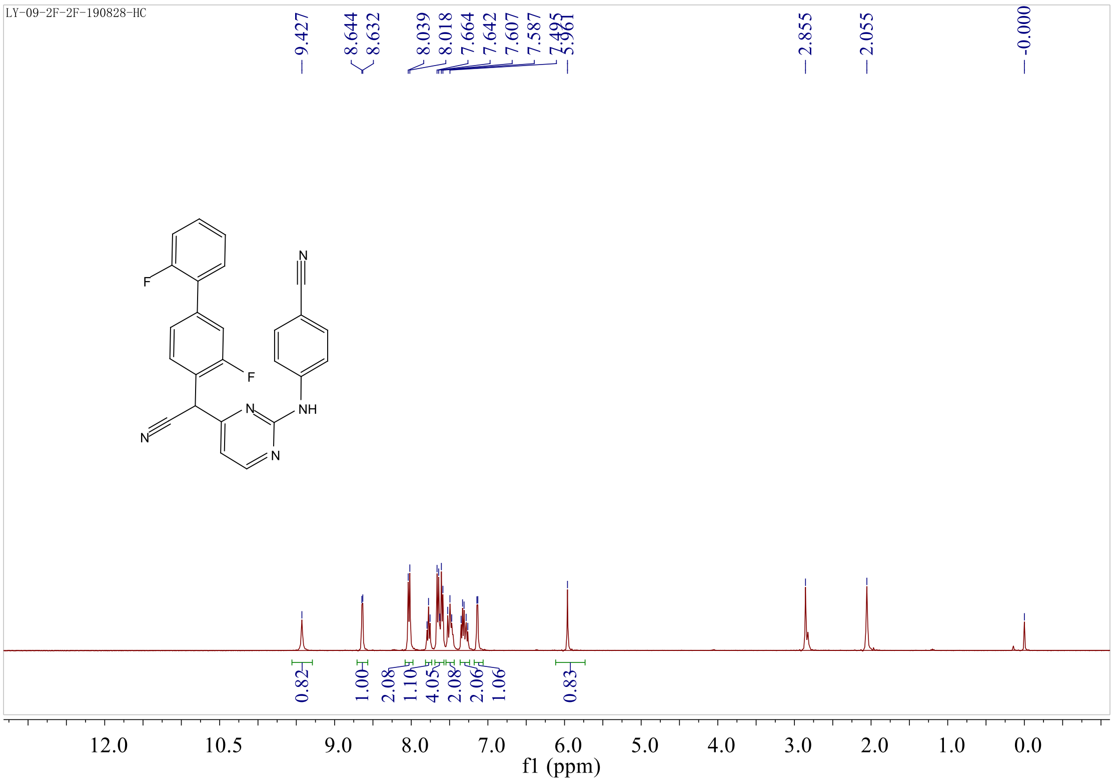

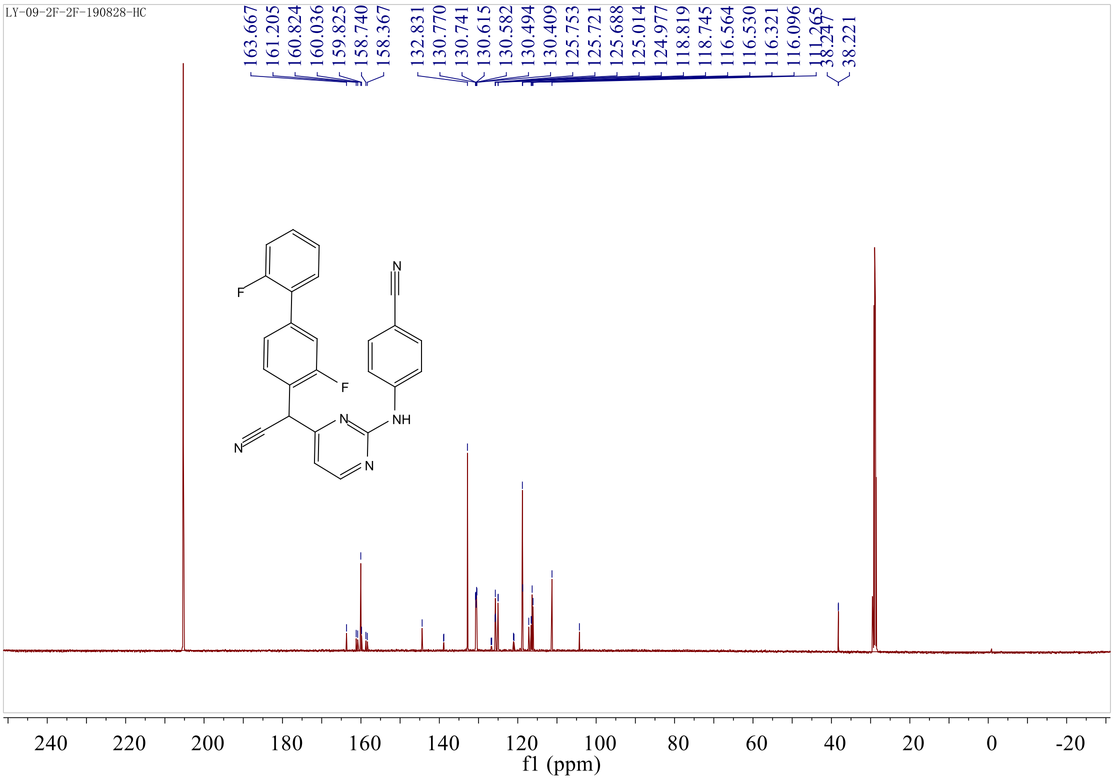

Display Report

Analysis Info

Analysis Name	D:\Data\data\2019\2F-2F_RD5_01_2862.d	Acquisition Date	12/18/2019 17:08:37 PM
Method	MS-2MIN-NEG.m	Operator	BDAL@DE
Sample Name	2F-2F	Instrument	compact 8255754.20127
Comment			

Acquisition Parameter

Source Type	ESI	Ion Polarity	Negative	Set Nebulizer	2.0 Bar
Focus	Active	Set Capillary	2800 V	Set Dry Heater	200 °C
Scan Begin	50 m/z	Set End Plate Offset	-500 V	Set Dry Gas	8.0 l/min
Scan End	1500 m/z	Set Charging Voltage	2000 V	Set Divert Valve	Waste
		Set Corona	0 nA	Set APCI Heater	0 °C

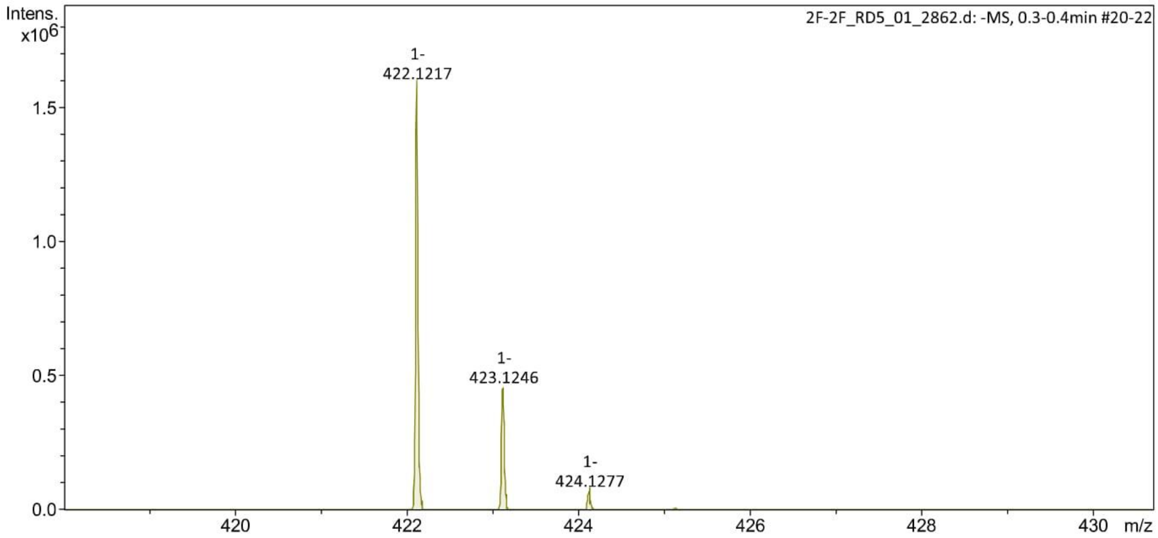

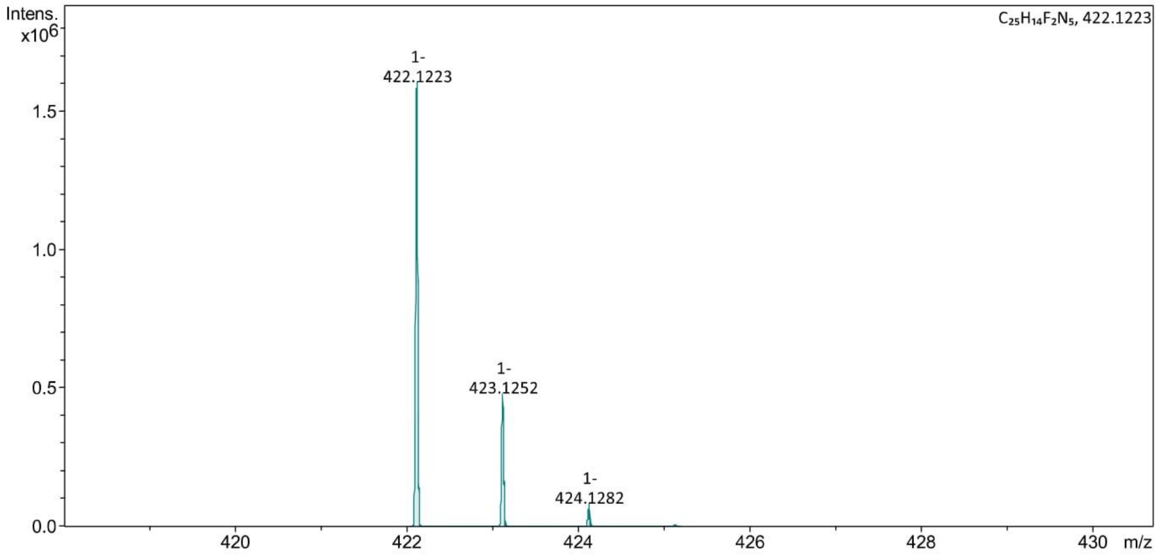

11. Spectra of compound 10k

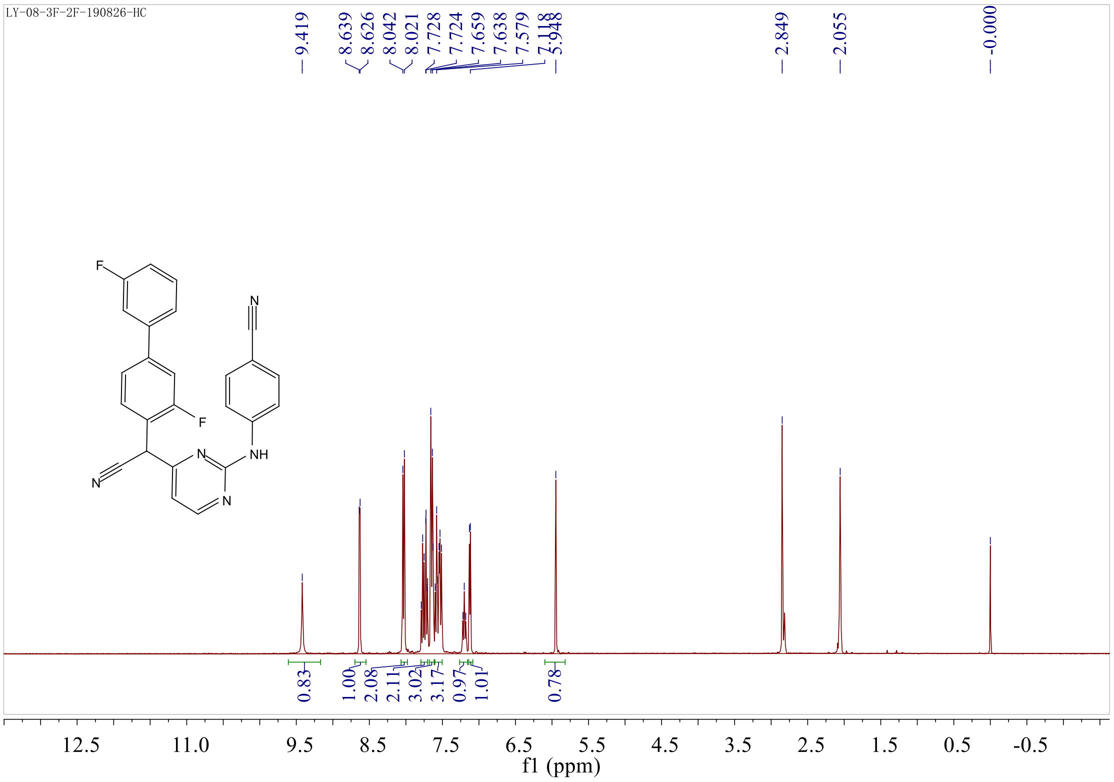

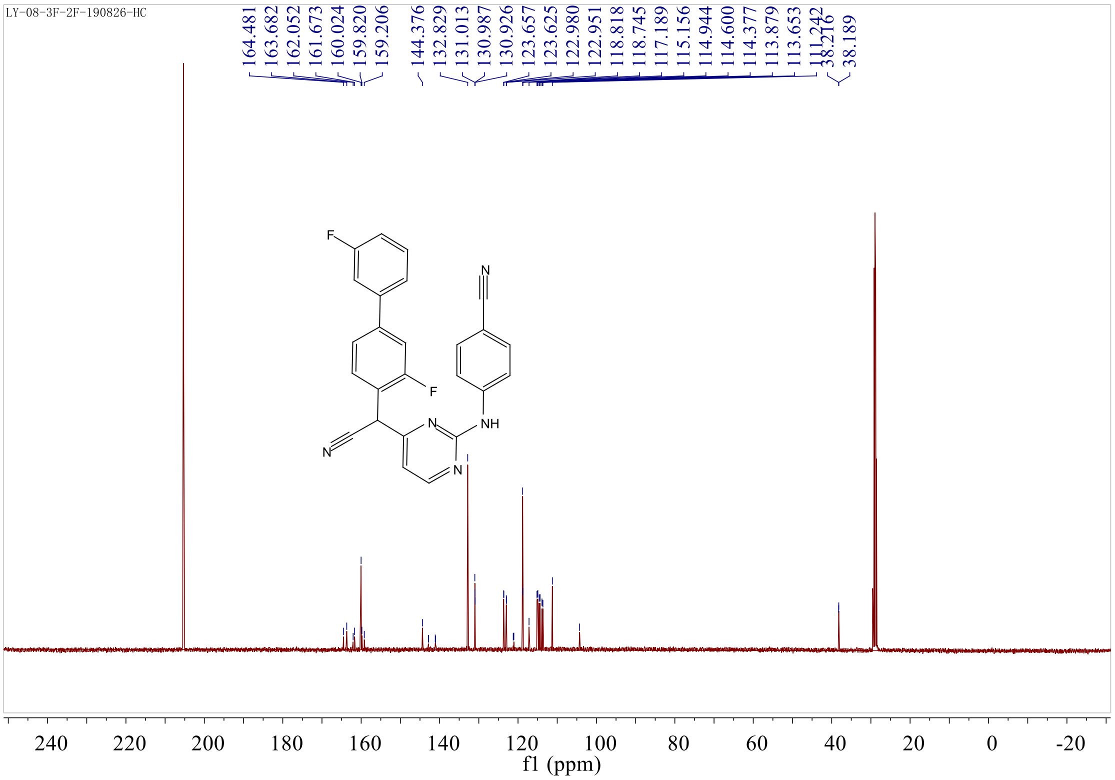

Display Report

Analysis Info

Analysis Name	D:\Data\data\2019\LY-3F-2F_RE6_01_2079.d	Acquisition Date	7/15/2019 16:34:05 PM
Method	MS-2MIN-POS-01.m	Operator	BDAL@DE
Sample Name	LY-3F-2F	Instrument	compact 8255754.20127
Comment			

Acquisition Parameter

Source Type	ESI	Ion Polarity	Positive	Set Nebulizer	2.0 Bar
Focus	Active	Set Capillary	4500 V	Set Dry Heater	200 °C
Scan Begin	50 m/z	Set End Plate Offset	-500 V	Set Dry Gas	8.0 l/min
Scan End	3000 m/z	Set Charging Voltage	2000 V	Set Divert Valve	Waste
		Set Corona	0 nA	Set APCI Heater	0 °C

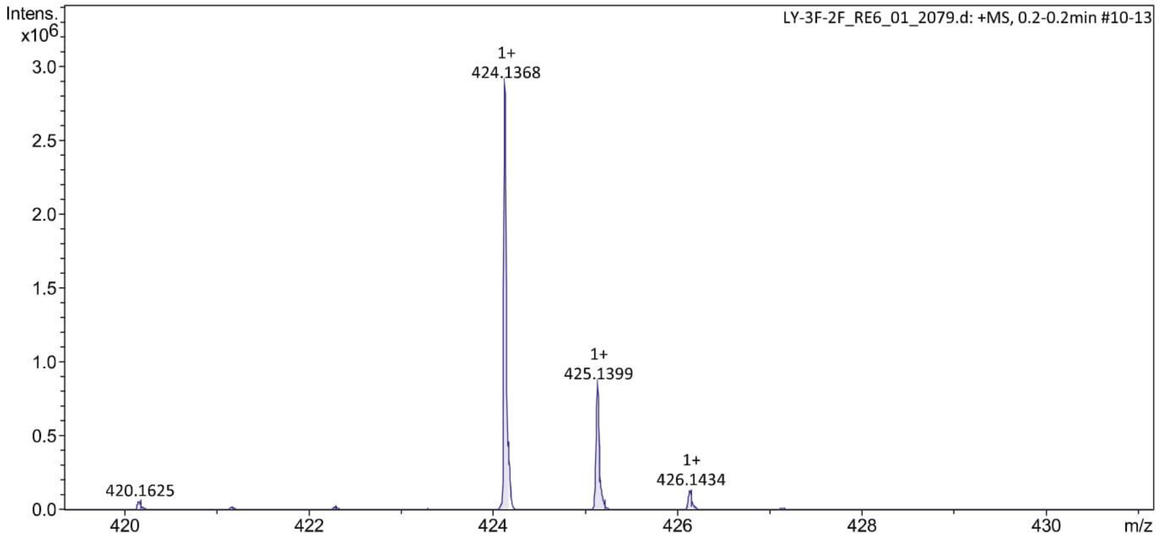

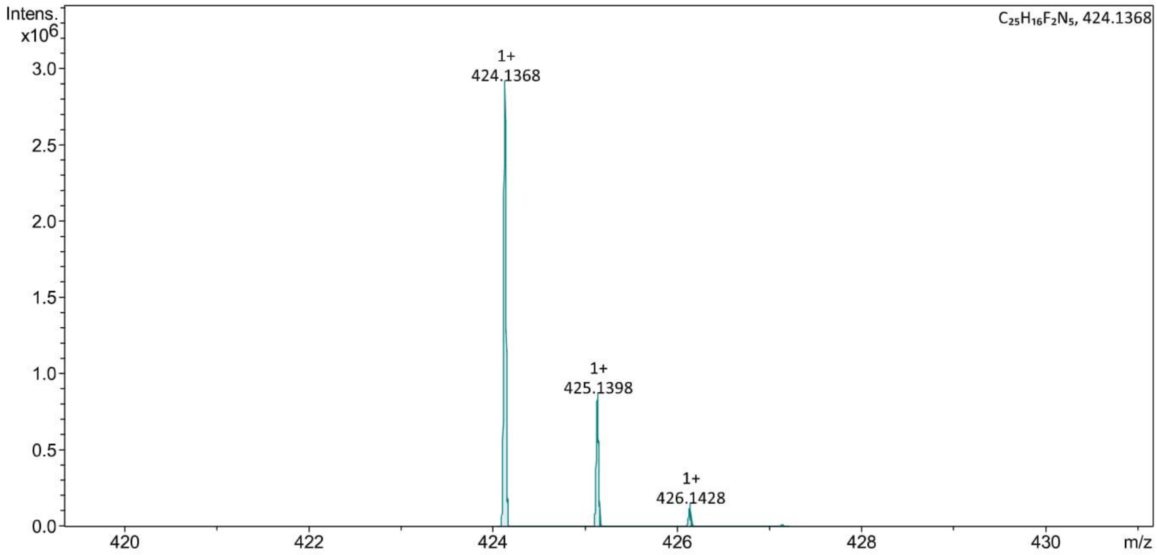

LY-3F-2F_RE6_01_2079.d

Bruker Compass DataAnalysis 4.3	printed: 7/15/2019 16:48:19 PM	by: BDAL@DE	Page 1 of 1

12. Spectra of compound 10k

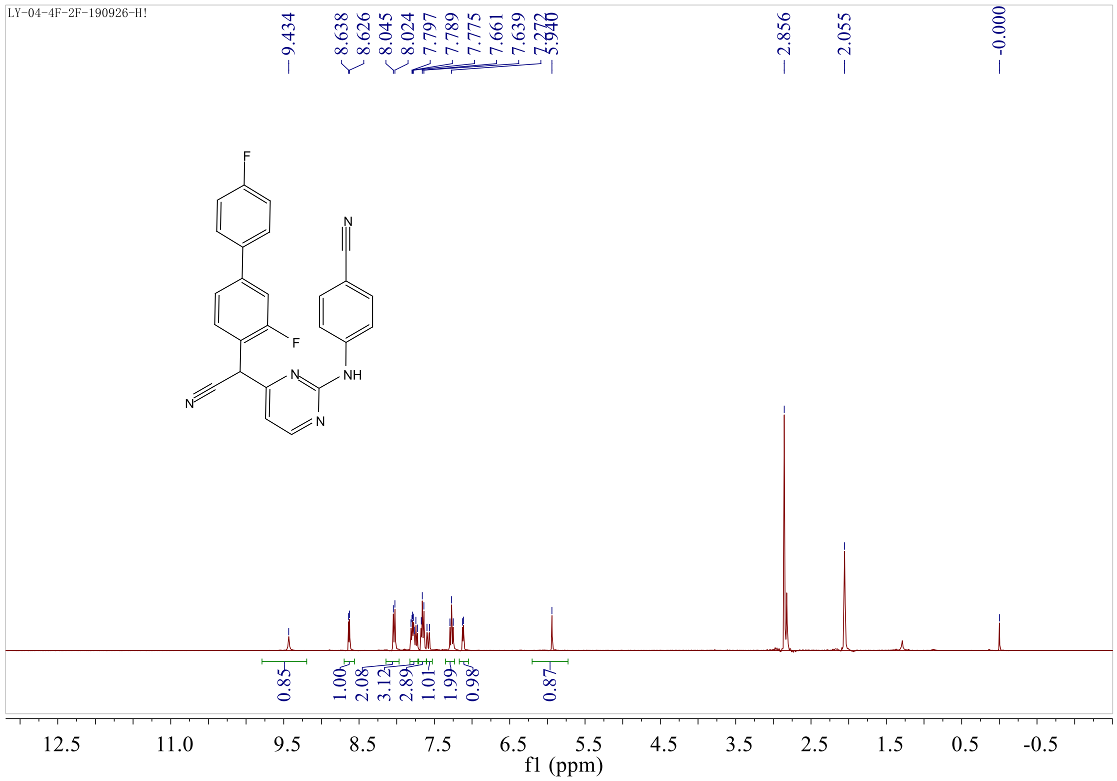

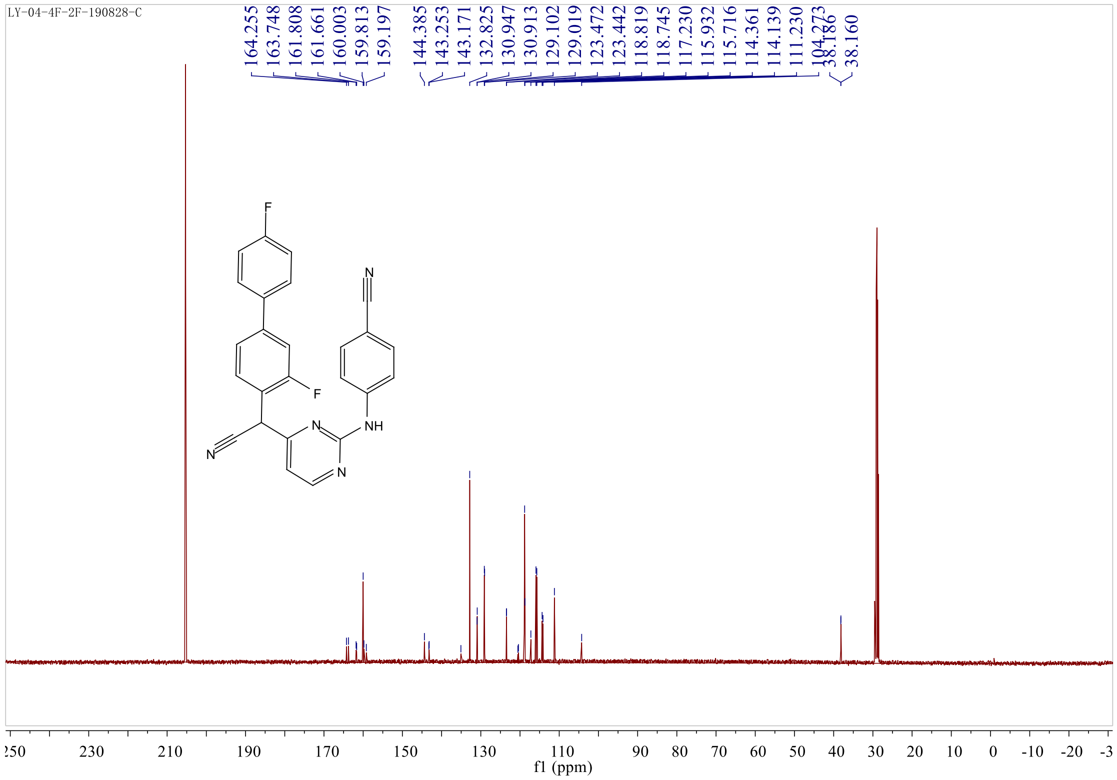

Display Report

Analysis Info

Analysis Name	D:\Data\data\2019\4F-2F_RD6_01_2861.d	Acquisition Date	12/18/2019 17:05:51 PM
Method	MS-2MIN-NEG.m	Operator	BDAL@DE
Sample Name	4F-2F	Instrument	compact 8255754.20127
Comment			

Acquisition Parameter

Source Type	ESI	Ion Polarity	Negative	Set Nebulizer	2.0 Bar
Focus	Active	Set Capillary	2800 V	Set Dry Heater	200 °C
Scan Begin	50 m/z	Set End Plate Offset	-500 V	Set Dry Gas	8.0 l/min
Scan End	1500 m/z	Set Charging Voltage	2000 V	Set Divert Valve	Waste
		Set Corona	0 nA	Set APCI Heater	0 °C

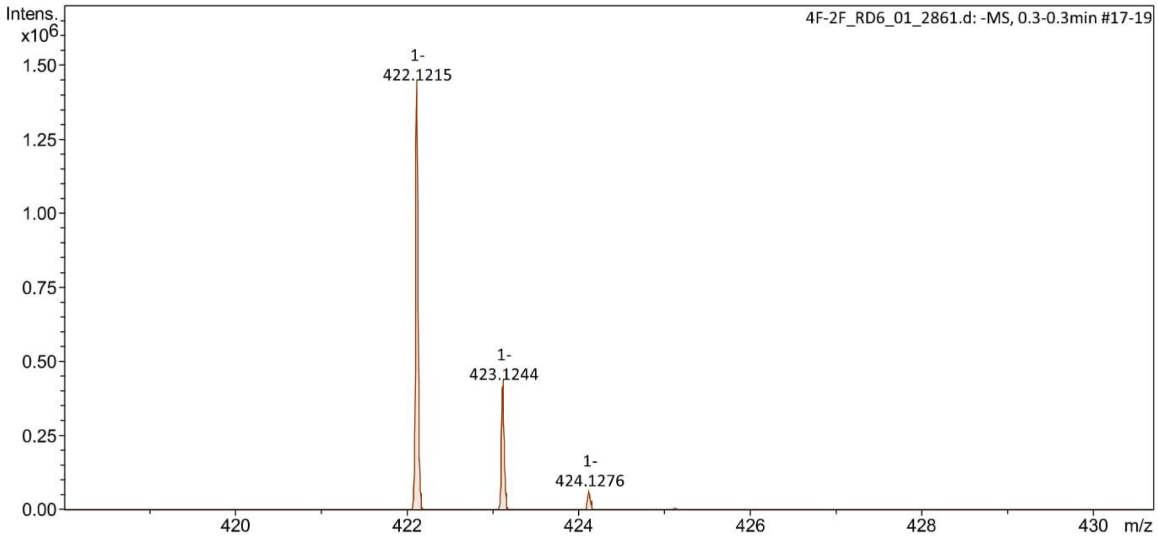

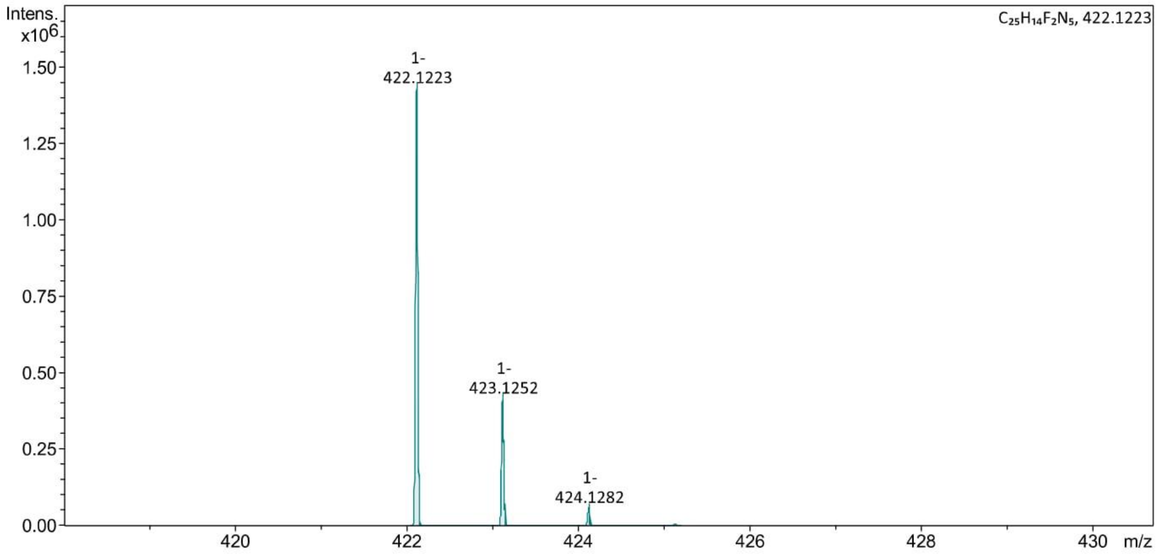

13. Spectra of compound 10m

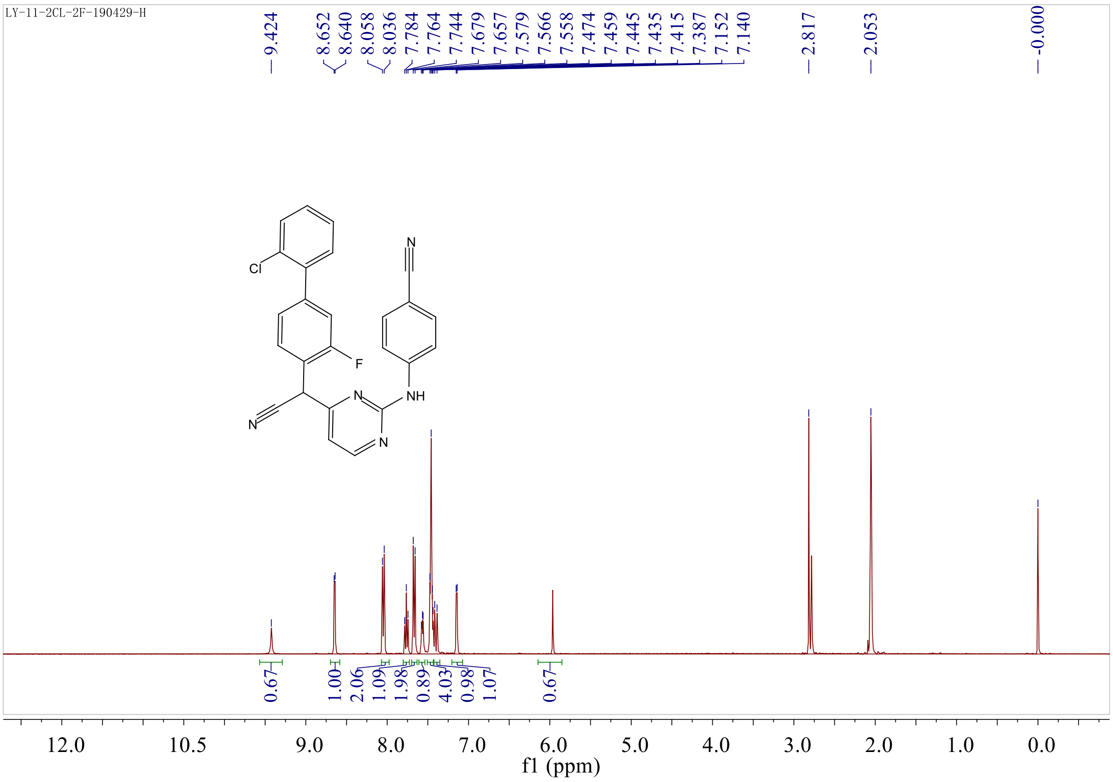

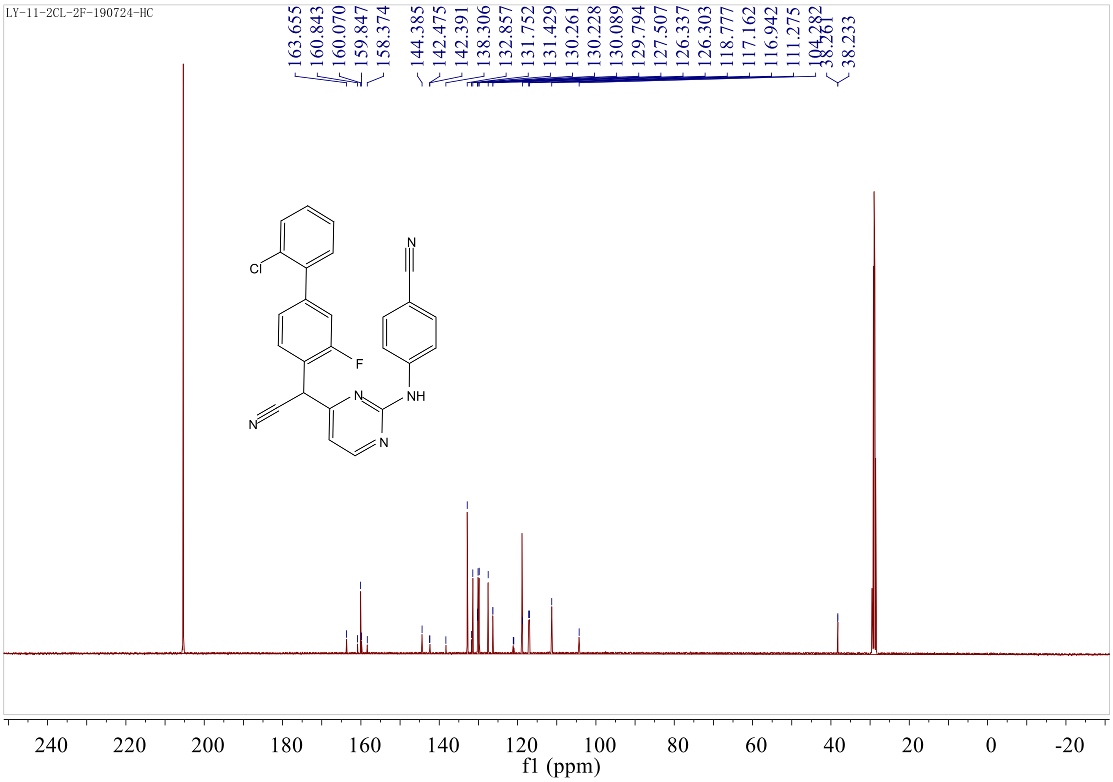

Display Report

Analysis Info

Analysis Name	D:\Data\data\2019\2CL-2F_RD8_01_2859.d	Acquisition Date	12/18/2019 17:00:20 PM
Method	MS-2MIN-NEG.m	Operator	BDAL@DE
Sample Name	2CL-2F	Instrument	compact 8255754.20127
Comment			

Acquisition Parameter

Source Type	ESI	Ion Polarity	Negative	Set Nebulizer	2.0 Bar
Focus	Active	Set Capillary	2800 V	Set Dry Heater	200 °C
Scan Begin	50 m/z	Set End Plate Offset	-500 V	Set Dry Gas	8.0 l/min
Scan End	1500 m/z	Set Charging Voltage	2000 V	Set Divert Valve	Waste
		Set Corona	0 nA	Set APCI Heater	0 °C

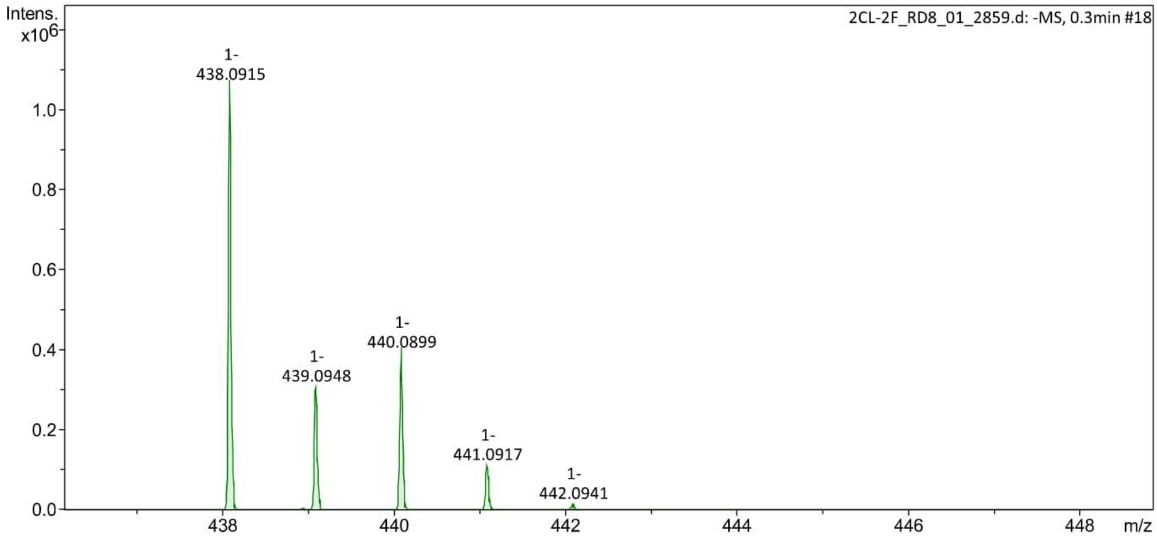

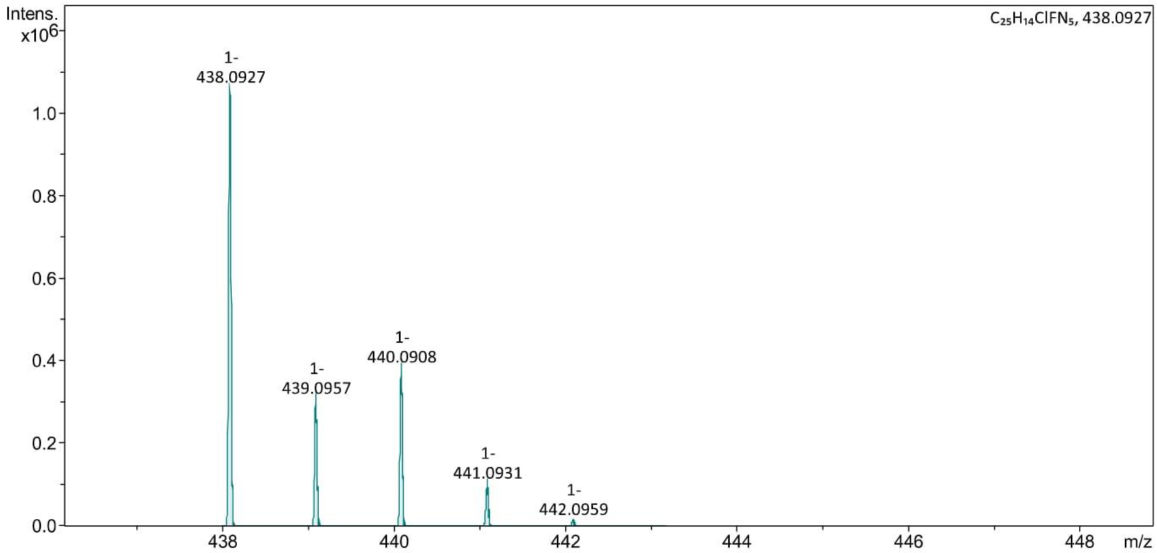

2CL-2F_RD8_01_2859.d

14. Spectra of compound 10n

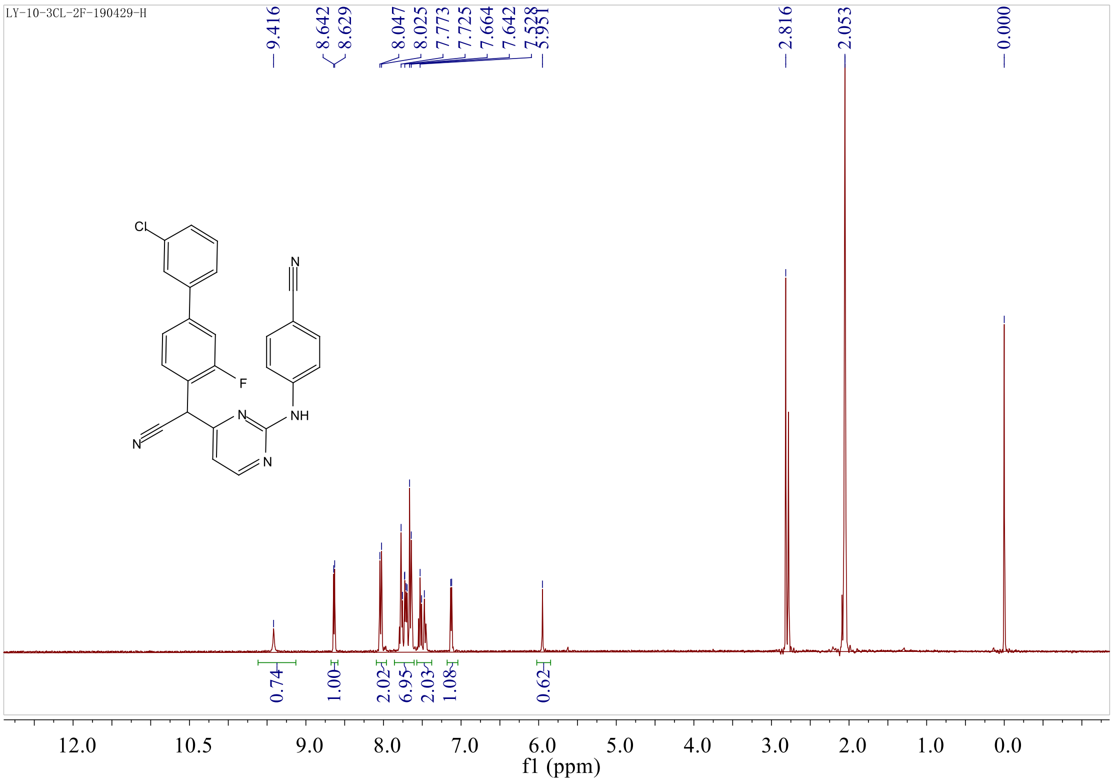

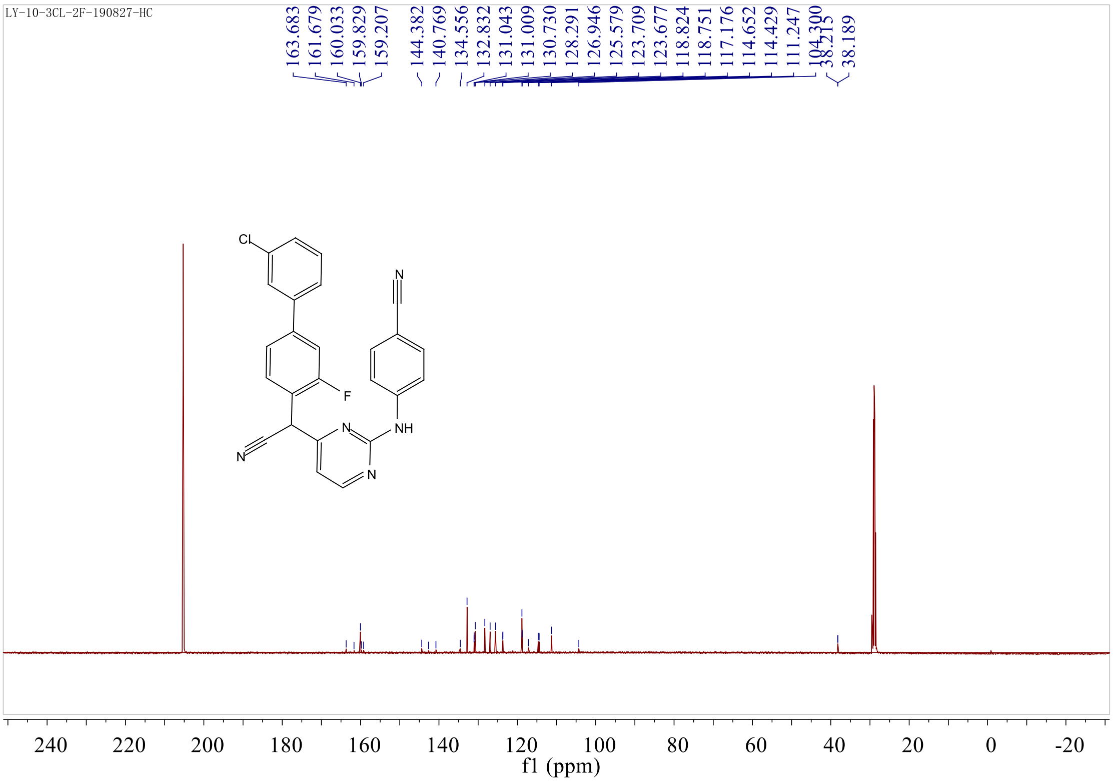

Display Report

Analysis Info

Analysis Name	D:\Data\data\2019\3CL-2F_RC6_01_2869.d	Acquisition Date	12/18/2019 17:27:57 PM
Method	MS-2MIN-NEG.m	Operator	BDAL@DE
Sample Name	3CL-2F	Instrument	compact 8255754.20127
Comment			

Acquisition Parameter

Source Type	ESI	Ion Polarity	Negative	Set Nebulizer	2.0 Bar
Focus	Active	Set Capillary	2800 V	Set Dry Heater	200 °C
Scan Begin	50 m/z	Set End Plate Offset	-500 V	Set Dry Gas	8.0 l/min
Scan End	1500 m/z	Set Charging Voltage	2000 V	Set Divert Valve	Waste
		Set Corona	0 nA	Set APCI Heater	0 °C

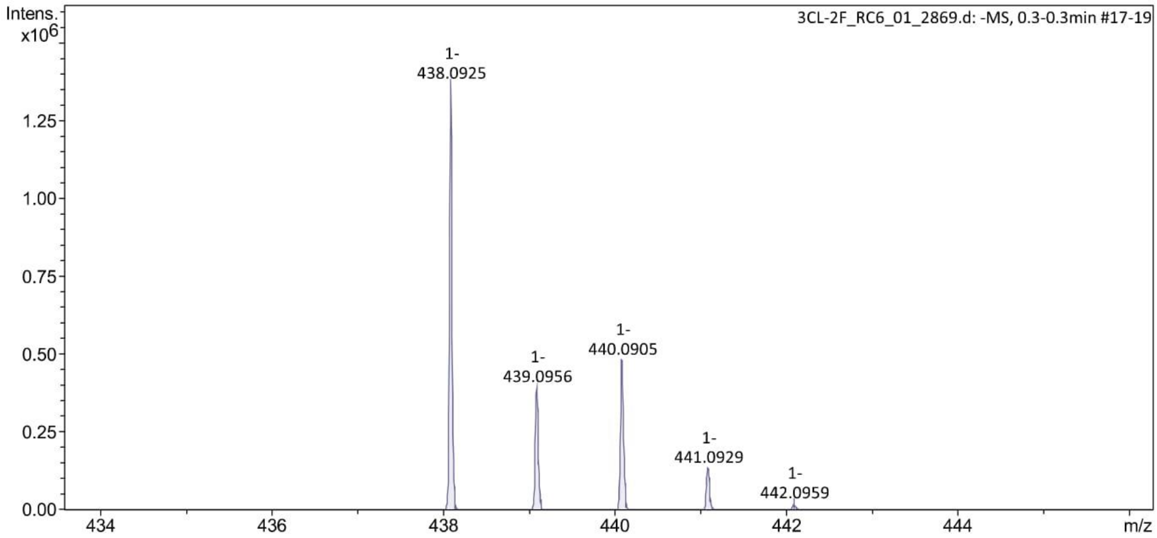

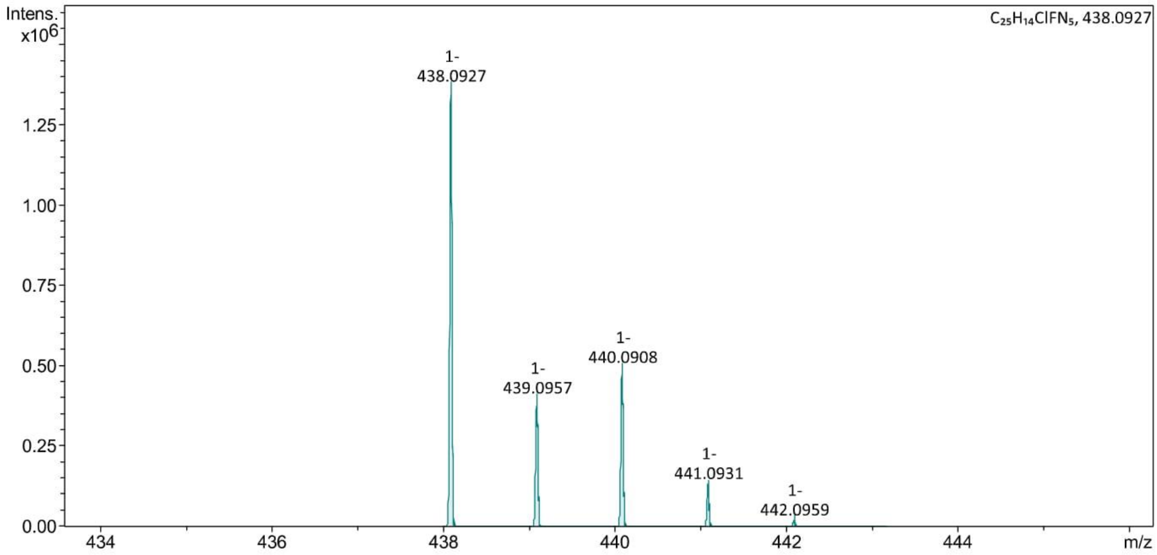

15. Spectra of compound 10o

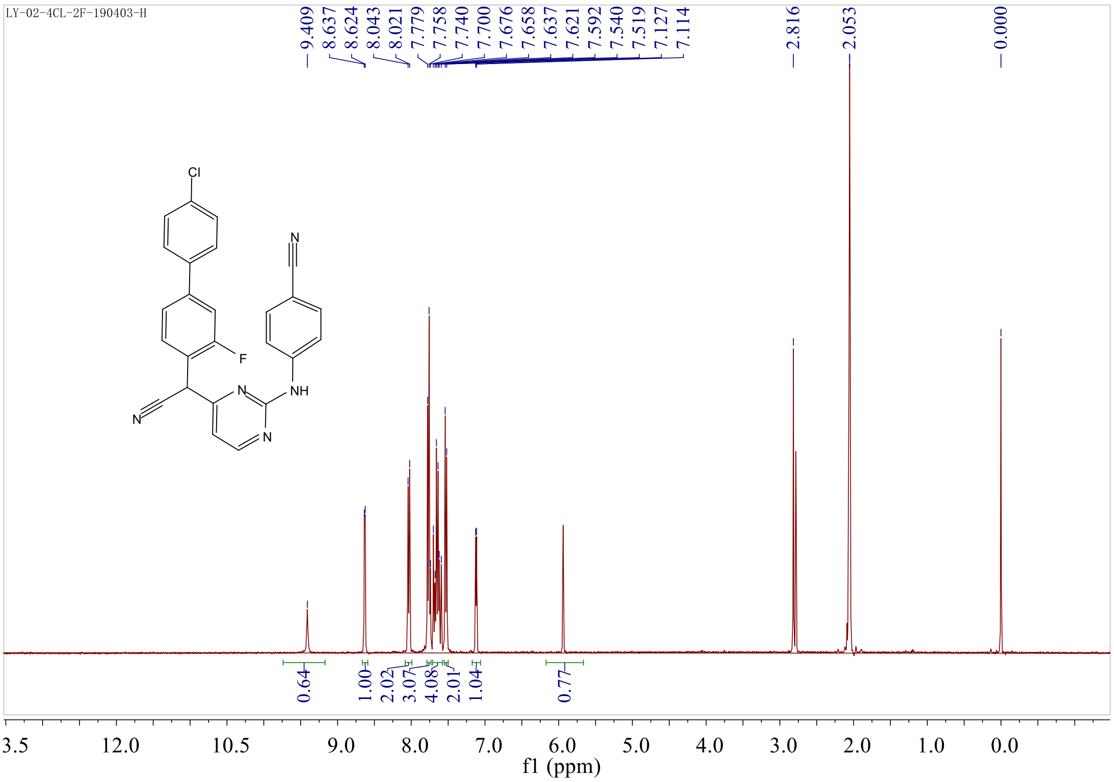

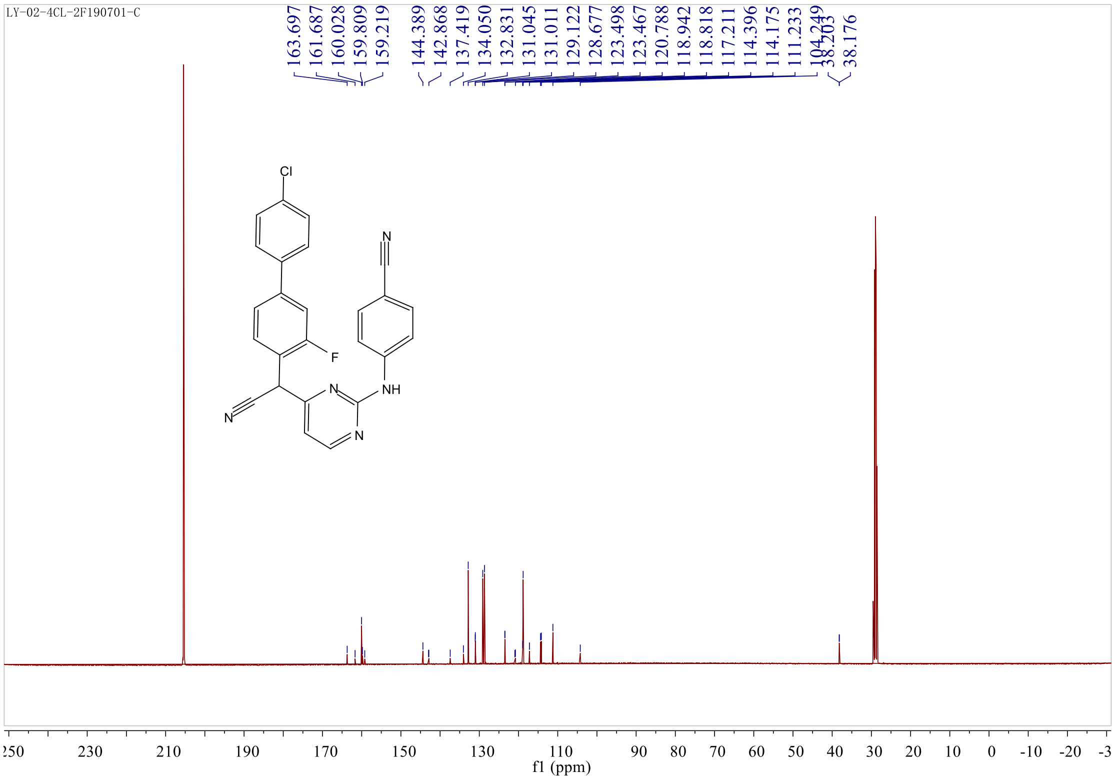

Display Report

Analysis Info

Analysis Name	D:\Data\data\2019\4CL-2F_RD7_01_2860.d	Acquisition Date	12/18/2019 17:03:06 PM
Method	MS-2MIN-NEG.m	Operator	BDAL@DE
Sample Name	4CL-2F	Instrument	compact 8255754.20127
Comment			

Acquisition Parameter

Source Type	ESI	Ion Polarity	Negative	Set Nebulizer	2.0 Bar
Focus	Active	Set Capillary	2800 V	Set Dry Heater	200 °C
Scan Begin	50 m/z	Set End Plate Offset	-500 V	Set Dry Gas	8.0 l/min
Scan End	1500 m/z	Set Charging Voltage	2000 V	Set Divert Valve	Waste
		Set Corona	0 nA	Set APCI Heater	0 °C

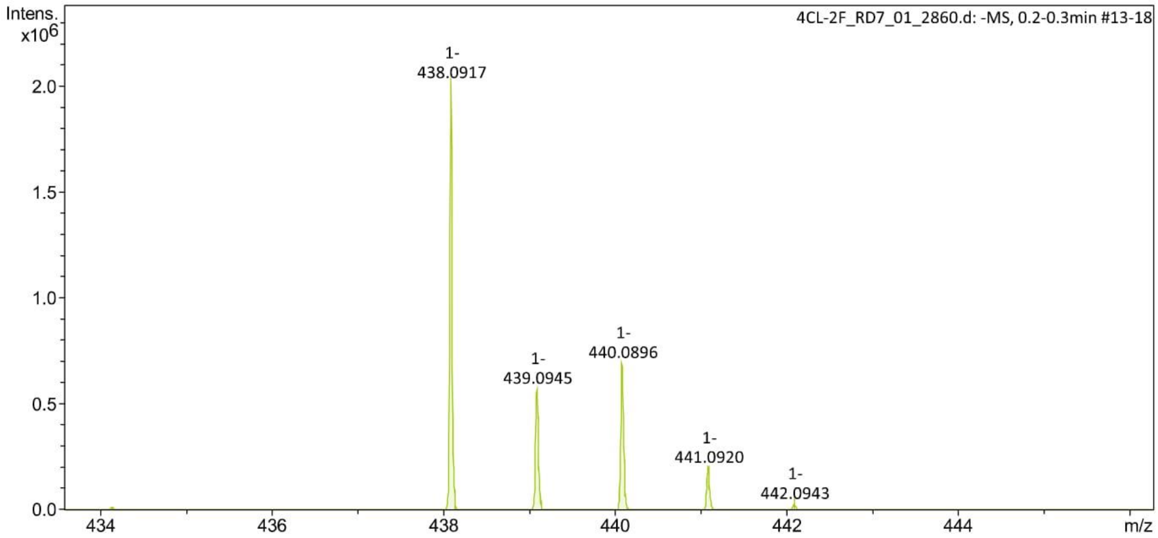

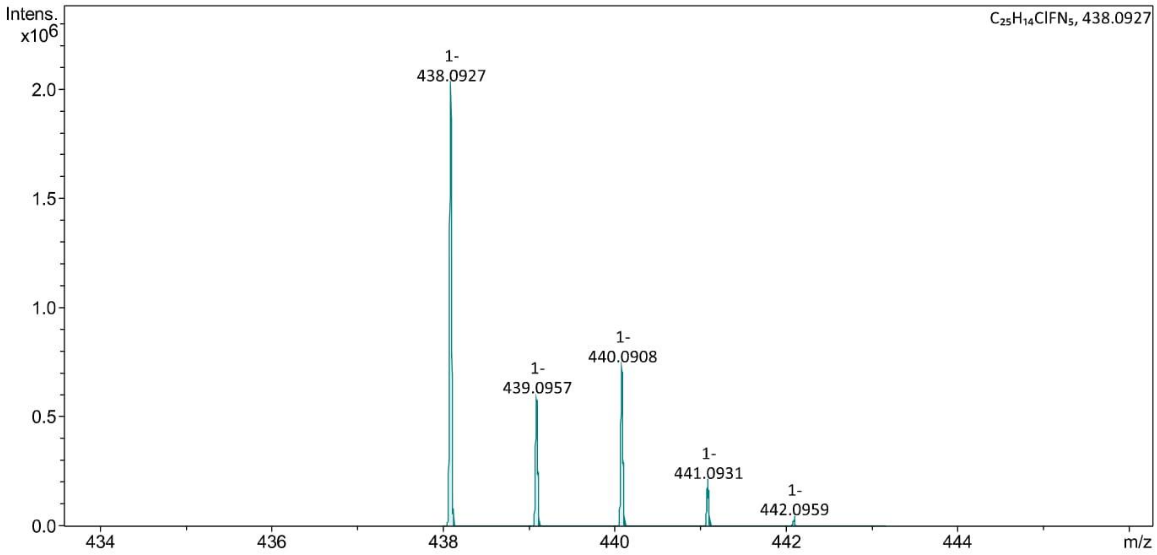

4CL-2F_RD7_01_2860.d

16. Spectra of compound 10p

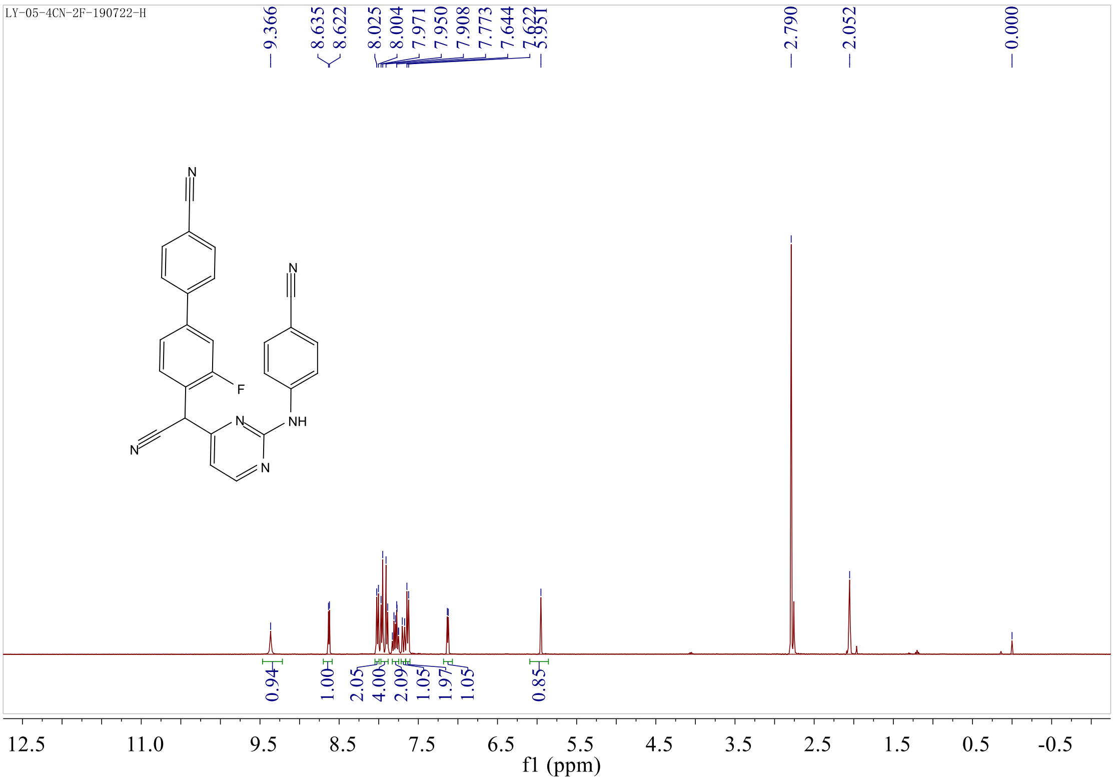

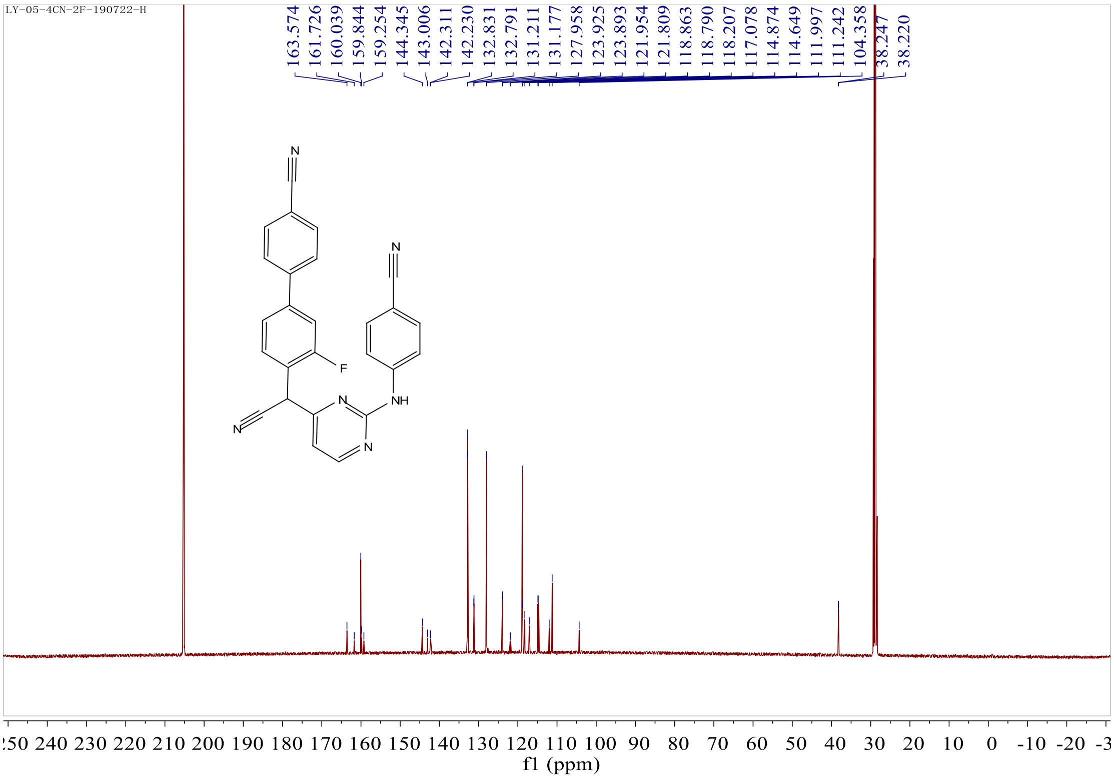

Display Report

Analysis Info

Analysis Name	D:\Data\data\2019\LY191212-4CN-2F_GD8_01_2850.d	Acquisition Date	12/18/2019 12:26:12 PM
Method	MS-2MIN-NEG.m	Operator	BDAL@DE
Sample Name	LY191212-4CN-2F	Instrument	compact 8255754.20127
Comment			

Acquisition Parameter

Source Type	ESI	Ion Polarity	Negative	Set Nebulizer	2.0 Bar
Focus	Active	Set Capillary	2800 V	Set Dry Heater	200 °C
Scan Begin	50 m/z	Set End Plate Offset	-500 V	Set Dry Gas	8.0 l/min
Scan End	1500 m/z	Set Charging Voltage	2000 V	Set Divert Valve	Waste
		Set Corona	0 nA	Set APCI Heater	0 °C

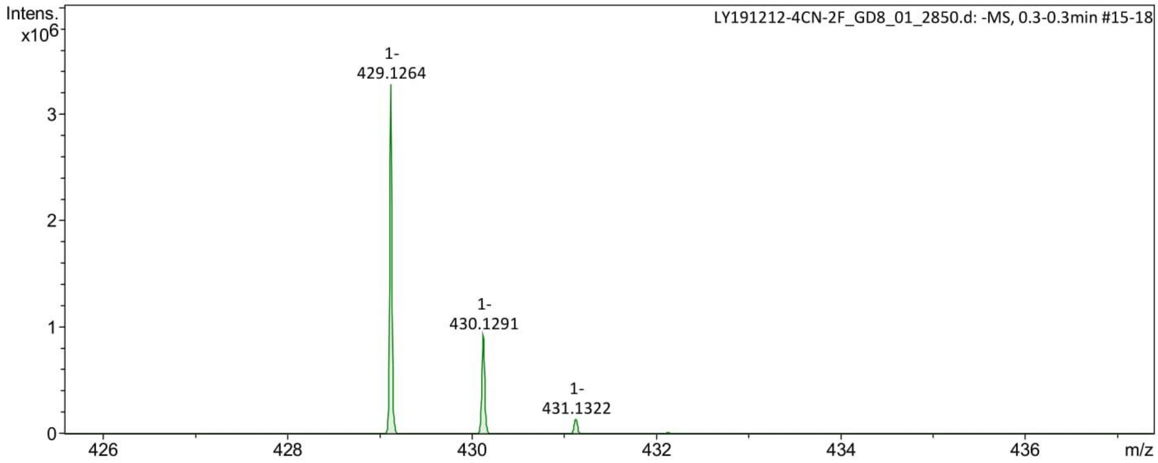

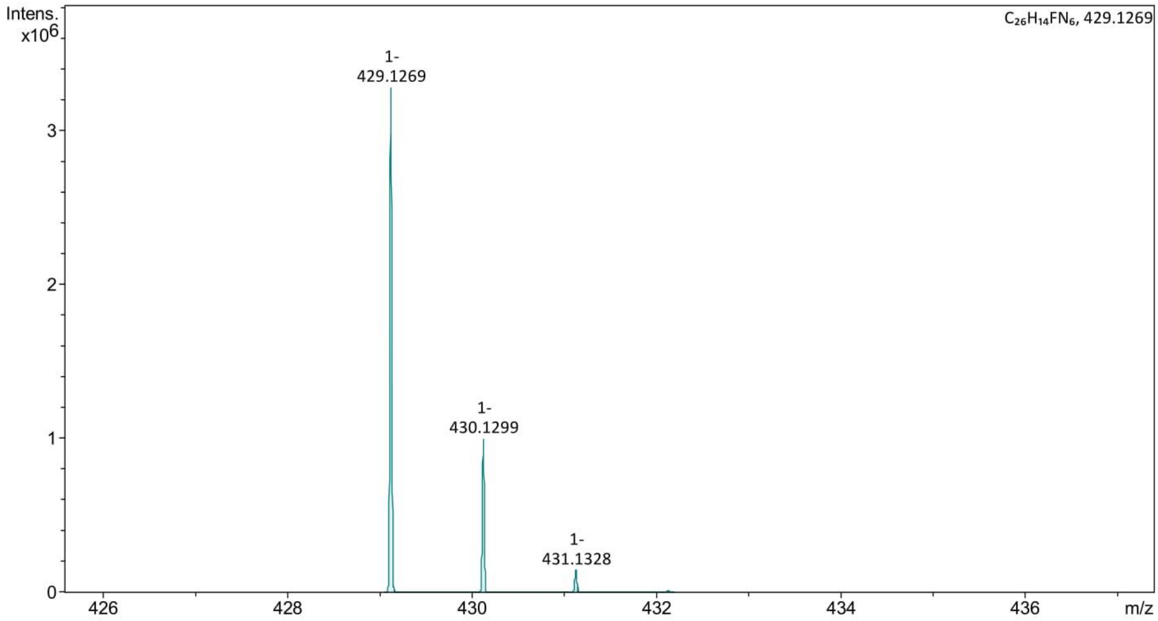

LY191212-4CN-2F_GD8_01_2850.d

Bruker Compass DataAnalysis 4.3 printed: 1/7/2020 11:20:30 AM by: BDAL@DE Page 1 of 1
